# Telemedicine-supported lifestyle intervention for glycemic control in patients with CHD and T2DM: multicenter, randomized controlled trial

**DOI:** 10.1038/s41591-025-03498-w

**Published:** 2025-02-07

**Authors:** Stephan Mueller, Sophia M. T. Dinges, Felix Gass, Isabel Fegers-Wustrow, Julian Treitschke, Pia von Korn, Alessandra Boscheri, Janosch Krotz, Felix Freigang, Clara Dubois, Ephraim B. Winzer, Axel Linke, Frank Edelmann, Anna Feuerstein, Oliver Wolfram, Kerstin Schäfer, Marlo Verket, Bernd Wolfarth, Marcus Dörr, Rolf Wachter, Björn Hackenberg, Sarah Rust, Thomas Nebling, Volker Amelung, Martin Halle

**Affiliations:** 1 Technical University of Munich, School of Medicine and Health, Department for Preventive Sports Medicine and Sports Cardiology, TUM University Hospital, Munich, Germany; 2 DZHK (German Centre for Cardiovascular Research), Partner Site Munich Heart Alliance, Munich, Germany; 3 KIZ—Kardiologie im Zentrum, Munich, Germany; 4 Institute for Applied Healthcare Research GmbH (inav), Berlin, Germany; 5 Department for Internal Medicine and Cardiology, Technische Universität Dresden, Heart Centre Dresden, University Hospital, Dresden, Germany; 6 Department of Cardiology, Angiology and Intensive Care Medicine, Campus Virchow Klinikum, Deutsches Herzzentrum der Charité, Berlin, Germany; 7 Charité-Universitätsmedizin Berlin, Corporate Member of Freie Universität Berlin and Humboldt-Universität zu Berlin, Berlin, Germany; 8 DZHK (German Centre for Cardiovascular Research), Partner Site Berlin, Berlin, Germany; 9 Department of Cardiology and Angiology, University Hospital Magdeburg, Magdeburg, Germany; 10 Department of Internal Medicine I, University Hospital Aachen, Aachen, Germany; 11 Department of Sports Medicine, Humboldt University and Charité University School of Medicine, Berlin, Germany; 12 Department of Internal Medicine B, University Medicine Greifswald, Greifswald, Germany; 13 DZHK (German Centre for Cardiovascular Research), Partner Site Greifswald, Greifswald, Germany; 14 Department of Cardiology, University Hospital Leipzig, Leipzig, Germany; 15 Department of Cardiology and Pneumology, University Medical Center Göttingen, Göttingen, Germany; 16 DZHK (German Centre for Cardiovascular Research), Partner Site Göttingen, Göttingen, Germany; 17 IDS Diagnostic Systems GmbH, Oberau, Germany; 18 Techniker Krankenkasse, Hamburg, Germany

**Keywords:** Preventive medicine, Lifestyle modification, Cardiovascular diseases, Metabolic disorders

## Abstract

Patients with coronary heart disease (CHD) and type 2 diabetes mellitus (T2DM) have a substantially increased risk for major cardiovascular events and mortality. Increasing physical activity and improving a healthy diet may effectively reduce cardiovascular risk factors; however, the effects are often transient. In a multicenter, 1:1 randomized controlled trial including 502 patients with combined CHD and T2DM (68 ± 8 years; 84% men), we assessed the effects of a home-based telemedicine-supported lifestyle intervention (exercise training, nutritional recommendations and health literacy training) with regular individualized feedback versus usual care. The study met its primary endpoint of reduced glycated hemoglobin after 6 months in favor of the lifestyle intervention group (mean between-group difference in the complete-case analysis (*n* = 197 and *n* = 193), −0.13% (95% confidence interval, −0.25 to −0.01), *P* = 0.04). When individualized feedback and health literacy training were discontinued after 6 months (while other telemedicine tools were maintained), no statistically significant between-group differences were observed at 12 months. At 12 months, 31 patients (6.2%) had a major adverse cardiovascular event (lifestyle intervention, *n* = 20 (8.0%); usual care, *n* = 11 (4.4%); *P* = 0.15), with the main reason being hospitalization for angina or revascularization (lifestyle intervention, *n* = 15; usual care, *n* = 8). There were five deaths (lifestyle intervention, *n* = 2; usual care, *n* = 3), none of which were categorized as related to the intervention. However, three events that resulted in hospitalization were categorized as potentially related to the intervention (decompensation of heart failure, vertebral disc prolapse and inguinal hernia). In conclusion, a home-based lifestyle intervention with telemedicine support showed modest effects in patients with CHD and T2DM. ClinicalTrials.gov registration: NCT03835923.

## Abstract (web summary)

A randomized controlled trial showed that a home-based, telemedicine-supported lifestyle intervention, which consisted of exercise training, nutrition recommendations and health literacy training, reported improved glycemic control after 6 months in patients with coronary heart disease and type 2 diabetes compared to usual care.

## Main

In 2021, 9.44 million deaths (95% confidence interval (CI), 8.82–9.96 million) and 185 million disability-adjusted life years worldwide (95% CI, 175–196 million) were attributed to coronary heart disease (CHD)^1^. Prognosis (including the risk for major adverse cardiac events (MACE), mortality and years of life lost) and quality of life (QoL) are substantially worse when CHD is also accompanied by type 2 diabetes mellitus (T2DM)^2,3^. Therefore, improving glycemic control is a major goal in patients with combined CHD and T2DM^4,5^.

Low physical activity and obesity are among the modifiable risk factors with the highest attributable burden on cardiovascular disease, and higher numbers of well-controlled risk factors lower the risk of long-term mortality in patients with CHD and concurrent T2DM^1,6^. In contrast, exercise training and diet have been shown to reduce the risk for hospitalization and cardiovascular mortality in patients with CHD^7,8^, as well as glycated hemoglobin (HbA1c), annual hospitalizations and healthcare costs in patients with T2DM^9–12^. Consequently, regular exercise training and achieving a body mass index (BMI) <25 kg m^−^^2^ are recommended in current CHD and T2DM guidelines^4,5,13^. However, despite evidence of physical activity interventions in patients with standalone conditions, only three trials to date have investigated the effects of exercise training compared to a control group in patients with both conditions. These trials did not show significant group differences in changes of HbA1c^14–16^.

As supervised lifestyle intervention programs are resource-intensive for caregivers and patients alike, and home-based interventions often reveal the disadvantage of low adherence rates, neither type of intervention seems to be sustainable in the long term. Telemedicine-supported home-based approaches have emerged as a potentially effective and cost-efficient alternative, also reaching patients who may not be able to take part in supervised on-site programs^17,18^. Additionally, smartphone applications enable the recording of data (for example, from exercise training), providing an objective measure of adherence that can also be used for individualized feedback. To further improve the effects of lifestyle interventions, it has also been recommended to individualize exercise recommendations and apply nutritional recommendations and health literacy education^19,20^.

Therefore, we initiated the randomized controlled ‘Lifestyle Intervention in CHD and T2DM (LeIKD) trial’ to assess the role and potential of a telemedicine-supported lifestyle intervention in older patients with combined CHD and T2DM. We hypothesized that 6 months of individualized, telemedicine-supported, home-based exercise training and nutritional counseling with regular feedback via telephone and e-mail, as well as health literacy education, will significantly reduce HbA1c compared to usual care and that these improvements can be maintained over an additional 6 months without regular feedback and health literacy education.

## Results

### Patient disposition

**Fig. 1:**
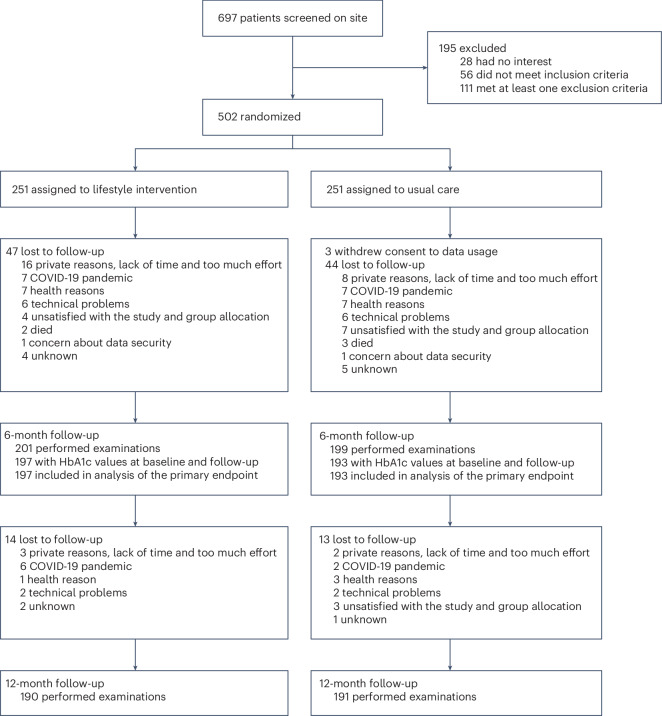
Patient flow from screening to 12-month follow-up. From 697 on-site screenings, 502 patients were randomized (1:1) to lifestyle intervention or usual care. Three patients withdrew their consent to data use and had all their data deleted. A total of 91 patients were lost to follow-up at 6 months and another 27 patients were lost to follow-up between 6 and 12 months. All 390 patients with available HbA1c measurements at baseline and 6 months were included in the analysis of the primary endpoint (change in HbA1c at 6 months).

Patient recruitment started on 12 February 2019 and was prematurely stopped on 27 March 2020 due to the rising incidence of COVID-19 in Germany. The last patient completed the study on 16 April 2021. From 697 on-site screenings, 502 patients were enrolled and randomly assigned to lifestyle intervention (*n* = 251) or usual care (*n* = 251). A total of 94 patients (47 lifestyle intervention and 47 usual care) were lost until the 6-month follow-up, of whom 3 (all usual care) withdrew their consent to data usage. An additional 27 patients (14 lifestyle intervention and 13 usual care) were lost until the 12-month follow-up (Fig. 1). Due to intermediate study site closures during the COVID-19 pandemic, the first follow-up was performed at a median (interquartile range (IQR)) of 6.3 (6.0–7.4) months after randomization, and the second follow-up at 6.2 (6.0–6.9) months after the first follow-up or 12.9 (12.1–14.6) months after randomization. Demographic and clinical characteristics at baseline (16% female; mean age = 68 years (s.d., 8 years); 98% white; mean HbA1c = 6.9% (s.d., 0.9%)) are shown in Table 1. Differences between patients who continued until the 6-month follow-up and those who were lost to follow-up are shown in Supplementary Table 1.

**Table 1:** Baseline demographics

Parameters	Lifestyle intervention group (*n* = 251)	Usual care (*n* = 248)
Sex		
Male	208 (82.9%)	210 (84.7%)
Female	43 (17.1%)	38 (15.3%)
Age at inclusion (year)	68 (8)	68 (7)
Body mass index (kg m^−^^2^)	30.2 (5.0)	30.0 (4.6)
Resting heart rate (beats per min)	68 (11)	71 (12)
Blood pressure (mm Hg)		
Systolic	137 (17)	138 (18)
Diastolic	78 (10)	80 (10)
CHD and T2DM		
Duration of CHD (year)	9.3 (8.2)	8.2 (6.2)
CHD classification		
no relevant stenosis (<50%)	46 (18.3%)	41 (16.5%)
1 vessel disease	53 (21.1%)	62 (25.0%)
2 vessel disease	47 (18.7%)	37 (14.9%)
3 vessel disease	64 (25.5%)	74 (29.8%)
Left main coronary disease	5 (2.0%)	4 (1.6%)
Unknown	36 (14.3%)	29 (11.7%)
CCS score		
Grade 0—no angina	208 (82.9%)	196 (79.0%)
Grade I—angina only with strenuous physical activity	34 (13.5%)	39 (15.7%)
Grade II—slight limitation of ordinary activity	7 (2.8%)	12 (4.8%)
Grade III–IV—marked limitation of ordinary physical activity or inability to carry on any physical activity without discomfort	1 (0.4%)	1 (0.4%)
Previous myocardial infarction	91 (36.3%)	80 (32.3%)
Coronary revascularization	139 (55.4%)	135 (54.4%)
Coronary artery bypass graft	38 (15.1%)	44 (17.7%)
Duration of T2DM (year)	12.2 (8.9)	12.3 (7.9)
HbA1c		
Percentage	6.8 (0.9)	6.9 (1.0)
mmol mol^−1^	51 (9.8)	52 (10.9)
<7.0% (<53 mmol mol^−1^)	165 (66%)	152 (61%)
7.0–7.9% (53–63 mmol mol^−1^)	57 (23%)	65 (26%)
≥8.0% (≥64 mmol mol^−1^)	26 (10%)	31 (13%)
Number of oral antidiabetic agents		
0	42 (16.7%)	42 (16.9%)
1	97 (38.6%)	108 (43.5%)
2	82 (32.7%)	75 (30.2%)
≥3	30 (12.0%)	22 (8.9%)
Patients treated with insulin	73 (29.1%)	64 (25.8%)
Other cardiovascular risk factors and diseases		
Hypertension	233 (92.8%)	228 (91.9%)
Hyperlipidemia	216 (86.1%)	213 (85.9%)
Smoking		
No (never smoked)	102 (40.6%)	83 (33.5%)
Ex-smoker	124 (49.4%)	135 (54.4%)
Current	25 (10.0%)	30 (12.1%)
Heart failure		
HFpEF (LVEF ≥ 50%)	35 (13.9%)	33 (13.3%)
HFmrEF (LVEF 41–49%)	14 (5.6%)	16 (6.5%)
HFrEF (LVEF ≤ 40%)	10 (4.6%)	7 (2.8%)
Atrial fibrillation		
Paroxysmal	33 (13.1%)	31 (12.5%)
Persistent	8 (3.2%)	9 (3.6%)
Permanent	4 (1.6%)	11 (4.4%)
Exercise capacity		
Peak oxygen consumption		
ml kg^−1^ min^−1^	18.7 (4.8)	18.6 (4.4)
Percentage of predicted norm values^a^	82.2 (17.4)	81.6 (13.6)
Level of education		
Low	8 (3.4%)	4 (1.7%)
Medium	112 (47.5%)	106 (45.7%)
High	116 (49.2%)	122 (52.6%)
Technical competencies		
Having your own mobile device		
Yes	205 (81.7%)	195 (78.6%)
No	33 (13.1%)	32 (12.9%)
Usage of mobile applications		
Daily	153 (61.0%)	141 (56.9%)
Weekly	21 (8.4%)	21 (8.5%)
Less than weekly/never	64 (25.5%)	65 (26.2%)
Handling of technical devices		
Rather easy	47 (18.7%)	30 (12.1%)
Rather difficult	173 (68.9%)	167 (67.3%)
Unknown	15 (6.0%)	29 (8.5%)

Data are mean (s.d.) or *n* (%). The level of education was based on the International Standard Classification of Education 2011 according to the highest self-reported graduation and/or professional training.

^a^Based on the normative data from the Study of Health in Pomerania (SHIP) study^36^.

CCS, Canadian Cardiovascular Society; HFpEF, heart failure with preserved ejection fraction; HFmrEF, heart failure with mildly reduced ejection fraction; HFrEF, heart failure with reduced ejection fraction; LVEF, left ventricular ejection fraction.

### Primary outcome

**Extended Data Fig. 1:**
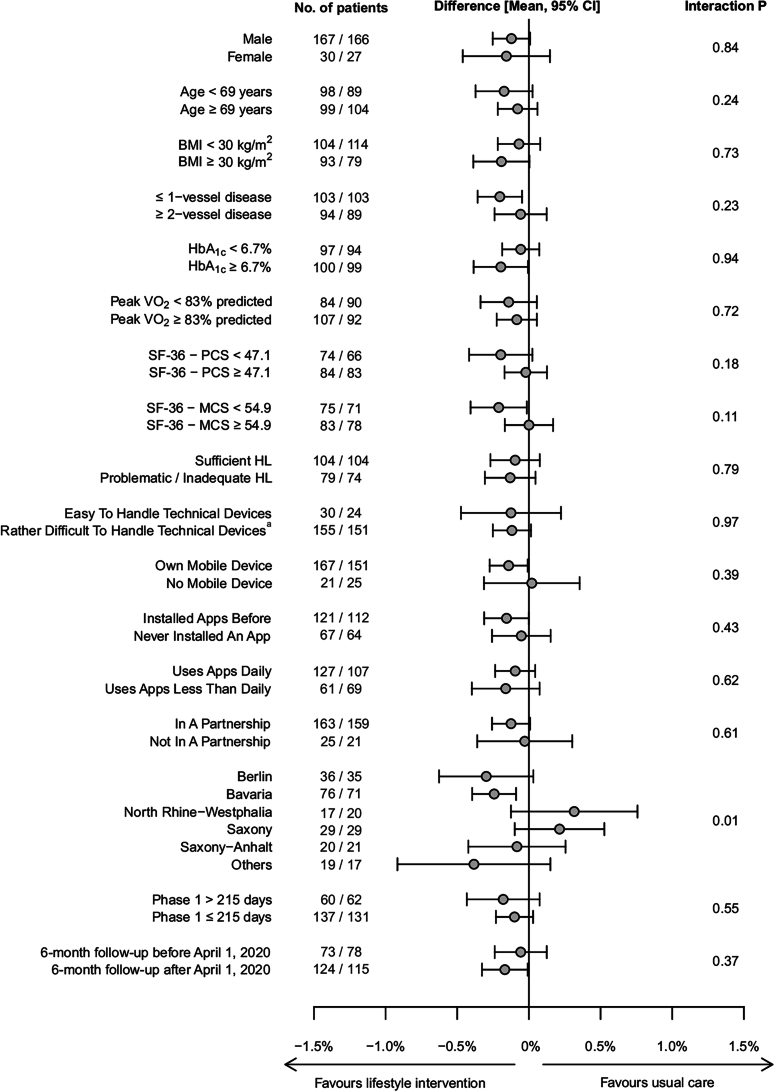
Results of the subgroup analysis for the primary endpoint (change in HbA1c after 6 months). Subgroup analyses were performed by fitting linear regression models with tests for interaction between these variables and study groups to the data. All tests were two-sided, and p values were not adjusted for multiple comparisons. Number of patients refers to ‘n (lifestyle intervention)/n (usual care)’ for each subgroup. FU, follow-up; HbA1c, glycated hemoglobin; HL, health literacy; MCS, mental component score; PCS, physical component score; VO_2_, oxygen consumption. ^a^Combination of the two answers ‘difficult’ (*n* = 270) and ‘unknown’ (*n* = 36).

At 6 months, the mean change in HbA1c modestly differed between lifestyle intervention and usual care (difference in mean changes, −0.13% (95% CI, −0.25 to −0.01%), *P* = 0.04; Table 2). Subgroup analysis did not reveal any significant interactions between the study group and the selected characteristics except for the federal state of the study sites (Extended Data Fig. 1).

**Table 2:** Results of the primary and secondary endpoints after 6 and 12 months

Parameters	Mean (s.d.; *n*)				Difference between lifestyle intervention and usual care	
	Lifestyle intervention		Usual care			
	Visit	Change from baseline	Visit	Change from baseline	Mean (95% CI; *n*)	*P* value
HbA1c (%)						
Baseline	6.79 (0.89; 248)	–	6.92 (0.97; 248)	–	–	–
6 months	6.68 (0.85; 200)	−0.13 (0.61; 197)	6.78 (0.78; 193)	0.00 (0.59; 193)	−0.13 (−0.25 to −0.01; 390)	0.04
12 months	6.84 (0.86; 180)	0.03 (0.72; 178)	6.85 (0.84; 174)	0.04 (0.67; 174)	−0.01 (−0.16 to 0.14; 352)	0.90
Body weight (kg)						
Baseline	92.2 (17.4; 251)	–	91.4 (15.9; 248)	–	–	–
6 months	90.0 (17.3; 201)	−2.2 (3.7; 201)	91.2 (15.5; 196)	−0.8 (3.2; 196)	−1.4 (−2.1 to −0.8; 397)	<0.001
12 months	89.4 (17.5; 187)	−2.2 (4.9; 187)	90.5 (15.8; 183)	−1.5 (4.3; 183)	−0.8 (−1.7 to 0.2; 370)	0.12
Waist circumference (cm)						
Baseline	107.8 (13.0; 243)	–	108.0 (11.9; 237)	–	–	–
6 months	106.3 (13.2; 189)	−1.5 (5.0; 187)	107.5 (12.0; 192)	−0.7 (5.3; 184)	−0.7 (−1.8 to 0.3; 371)	0.18
12 months	106.8 (13.0; 174)	−0.6 (5.5; 169)	107.1 (12.6; 171)	−1.4 (6.1; 165)	0.8 (−0.4 to 2.1; 334)	0.20
Systolic blood pressure (mm Hg)						
Baseline	137 (17; 251)	–	138 (18; 248)	–	–	–
6 months	133 (17; 201)	−5 (16; 201)	135 (17; 195)	−2 (18; 195)	−2 (−6 to 1; 396)	0.17
12 months	136 (19; 180)	−1.2 (18.4; 180)	135 (16; 175)	−2 (18; 175)	1 (−3 to 4; 355)	0.77
Diastolic blood pressure (mm Hg)						
Baseline	78 (10; 251)	–	80 (10; 248)	–	–	–
6 months	78 (9; 201)	0 (10; 201)	78 (10; 195)	−1 (11; 195)	0 (−2 to 3; 396)	0.63
12 months	79 (10; 180)	1 (11; 180)	78 (10; 175)	−1 (11; 175)	2 (−1 to 4; 355)	0.14
HDL cholesterol (mg dl^−1^)						
Baseline	48 (14; 250)	–	47 (12; 248)	–	–	–
6 months	49 (14; 200)	1 (12; 199)	46 (12; 194)	0 (8; 194)	2 (0–4; 393)	0.12
12 months	49 (12; 179)	0 (10; 179)	46.9 (11.8; 175)	0.6 (6.6; 175)	−0.4 (−2.2 to 1.4; 354)	0.64
LDL cholesterol (mg dl^−1^)						
Baseline	91 (36; 250)	–	93 (32; 246)	–	–	–
6 months	84 (30; 200)	−5 (23; 199)	87 (34; 194)	−6 (26; 192)	1 (−4 to 6; 391)	0.69
12 months	78 (28; 179)	−10 (31; 179)	85.7 (34.6; 175)	−8.3 (29.9; 173)	−1.6 (−8.0 to 4.7; 352)	0.61
Triglycerides (mg dl^−1^)						
Baseline	177 (101; 249)	–	188 (98; 248)	–	–	–
6 months	158 (84; 200)	−22 (72; 198)	178 (109; 194)	−10 (79; 194)	−12 (−27 to 3; 392)	0.12
12 months	157 (85; 178)	−15 (74; 177)	176 (115; 174)	−11 (89; 174)	−4 (−21 to 14; 351)	0.68
SF-36 physical component score (QoL)^a^						
Baseline	43.9 (10.2; 202)	–	45.1 (9.3; 191)	–	–	–
6 months	46.5 (9.4; 157)	0.9 (8.3; 133)	46.9 (9.2; 163)	1.4 (6.2; 128)	−0.5 (−2.3 to 1.3; 261)	0.58
12 months	46.7 (9.3; 150)	2.0 (8.3; 123)	45.6 (10.2; 147)	0.7 (8.0; 121)	1.3 (−0.7 to 3.4; 244)	0.20
SF-36 mental component score (QoL)^a^						
Baseline	52.0 (9.3; 202)	–	52.5 (9.0; 191)	–	–	–
6 months	53.0 (8.6; 157)	1.0 (8.2; 133)	51.1 (9.3; 163)	−1.6 (7.3; 128)	2.7 (0.8–4.6; 261)	0.006
12 months	51.4 (10.4; 150)	−0.6 (8.7; 123)	51.3 (10.0; 147)	−1.9 (8.8; 121)	1.3 (−0.9 to 3.5; 244)	0.23
HLS-EU-Q16—health literacy^b^						
Baseline	12.2 (3.6; 232)	–	12.6 (3.0; 227)	–	–	–
6 months	12.5 (3.5; 181)	0.5 (3.1; 171)	12.5 (3.0; 177)	−0.1 (2.6; 166)	0.6 (0.0–1.2; 337)	0.04
12 months	12.6 (3.2; 167)	0.5 (3.1; 158)	12.7 (3.2; 156)	0.0 (2.8; 147)	0.5 (−0.2 to 1.2; 305)	0.14
TFEQ—cognitive restraint of eating^c^						
Baseline	7.6 (4.1; 232)	–	7.8 (3.7; 225)	–	–	–
6 months	8.9 (4.4; 185)	1.2 (3.3; 175)	7.6 (3.7; 176)	0.2 (3.5; 161)	1.1 (0.3–1.8; 336)	0.005
12 months	8.4 (4.2; 169)	0.9 (3.4; 159)	7.8 (3.9; 159)	0.2 (3.1; 147)	0.7 (0.0–1.4; 306)	0.06
TFEQ—disinhibition^d^						
Baseline	4.4 (2.9; 234)	–	4.7 (3.0; 228)	–	–	–
6 months	4.2 (2.7; 188)	−0.4 (1.9; 178)	4.5 (2.9; 178)	−0.1 (2.0; 166)	−0.2 (−0.6 to 0.2; 344)	0.32
12 months	4.0 (2.7; 169)	−0.5 (1.9; 159)	4.1 (2.7; 161)	−0.5 (2.1; 151)	−0.1 (−0.5 to 0.4; 310)	0.81
TFEQ—hunger^e^						
Baseline	3.5 (2.9; 235)	–	3.9 (3.0; 228)	–	–	–
6 months	3.2 (2.7; 189)	−0.5 (2.0; 180)	3.5 (3.0; 179)	−0.6 (2.0; 166)	0.1 (−0.3 to 0.5; 346)	0.61
12 months	3.2 (2.7; 169)	−0.6 (2.1; 160)	3.2 (2.9; 161)	−0.6 (2.1; 151)	0.1 (−0.4 to 0.5; 311)	0.80
IPAQ—MET min						
Baseline	3,922 (2,964; 109)	–	3,173 (2,869; 119)	–	–	–
6 months	5,364 (3,592; 98)	1,447 (2,997; 56)	4,240 (3,568; 100)	1,129 (3,652; 56)	317 (−934 to 1,568; 112)	0.62
12 months	5,092 (3,899; 98)	808 (4,060; 54)	3,906 (3,048; 97)	925 (2,781; 54)	−117 (−1,445 to 1,211; 108)	0.86
Daily steps						
Baseline	5,617 (3,195; 179)	–	5,761 (3,131; 151)	–	–	–
6 months	6,112 (3,320; 151)	270 (2,726; 136)	5,833 (3,668; 144)	−49 (2,861; 119)	319 (−370 to 1,009; 255)	0.36
12 months	5,987 (3,298; 105)	−132 (2,971; 92)	5,540 (3,581; 118)	−241 (3,003; 95)	109 (−753 to 971; 187)	0.80

All outcomes presented in this table were evaluated with two-sided *t* tests for independent samples and were not adjusted for multiple comparisons. The exact *P* value for change in body weight at 6 months was 3.779 × 10^−5^.

^a^Higher scores indicate better QoL (score range = 0–100).

^b^Higher scores indicate better health literacy (score range = 0–16).

^c^Higher scores indicate better cognitive restraint (score range = 0–21).

^d^Higher scores indicate worse control (score range = 0–16).

^e^Higher scores indicate higher susceptibility for internal and external hunger signs (score range = 0–14).

MET, metabolic equivalent of task.

### Secondary outcomes

At 6 months, weight loss was significantly higher following lifestyle intervention compared with usual care (difference in mean changes = −1.4 kg (−2.1 to −0.8), *P* < 0.001). Furthermore, changes in the QoL mental component score (2.7 (0.8–4.6), *P* = 0.006), the eating behavior score for ‘cognitive restraint of eating’ (1.1 (0.3–1.8), *P* = 0.005) and health literacy (0.6 (0.0–1.2), *P* = 0.04) were significantly different between groups (Table 2). No significant group differences were observed for waist circumference, high-density lipoprotein (HDL) cholesterol, low-density lipoprotein (LDL) cholesterol, triglycerides, systolic blood pressure, diastolic blood pressure, the QoL physical component score, the eating behavior scores for disinhibition and hunger, average steps per day or the International Physical Activity Questionnaire (IPAQ) during the first 6 months (all *P* > 0.05; Table 2). After discontinuation of feedback and health literacy training, no significant differences between groups were observed for any of the investigated endpoints at 12 months (Table 2).

### Safety

During the complete study period, 31 patients (6.2%) had a 4P-MACE (lifestyle intervention, *n* = 20 (8.0%); usual care, *n* = 11 (4.4%); *P* = 0.15), of which 14 (lifestyle intervention, *n* = 8; usual care, *n* = 6; *P* = 0.80) had their event until 6-month follow-up. None of the patients had more than one 4P-MACE. The most frequent MACE was hospitalization due to angina or revascularization (lifestyle intervention, 15 events; usual care, 8 events; Extended Data Table 1). From the 20 patients with a 4P-MACE who were randomized to the lifestyle intervention group, seven (35.0%) were adherent to the exercise intervention with a median (IQR) exercise duration of 146 min per week (101–169), while the remaining 13 recorded a median (IQR) of 10 min per week (1–48) between baseline and the time of the event. Moreover, 14 (70.0%) of these patients fulfilled the adherence criteria for the nutritional intervention and lost an average of −1.8 ± 2.4 kg body weight between baseline and the last record before the event. Episodes of hypoglycemia were reported in three patients (lifestyle intervention, *n* = 1; usual care, *n* = 2). During the study, five patients died due to the progression of CHD (lifestyle intervention), multiorgan failure with decompensated heart failure (lifestyle intervention), acute coronary syndrome (usual care), bronchial carcinoma (usual care) or polytrauma after a traffic accident (usual care). Moreover, five patients were diagnosed with COVID-19 (lifestyle intervention, *n* = 2; usual care, *n* = 3), two of whom were hospitalized. No reported serious adverse event occurred during or within 2 h after exercise training. However, three events that resulted in a hospitalization were categorized as potentially related to the intervention. These included a decompensation of pre-existing heart failure (the patient performed exercise training during a gastrointestinal infection 4 weeks before the decompensation), a vertebral disc prolapse and an inguinal hernia.

### Adherence

**Extended Data Fig. 2:**
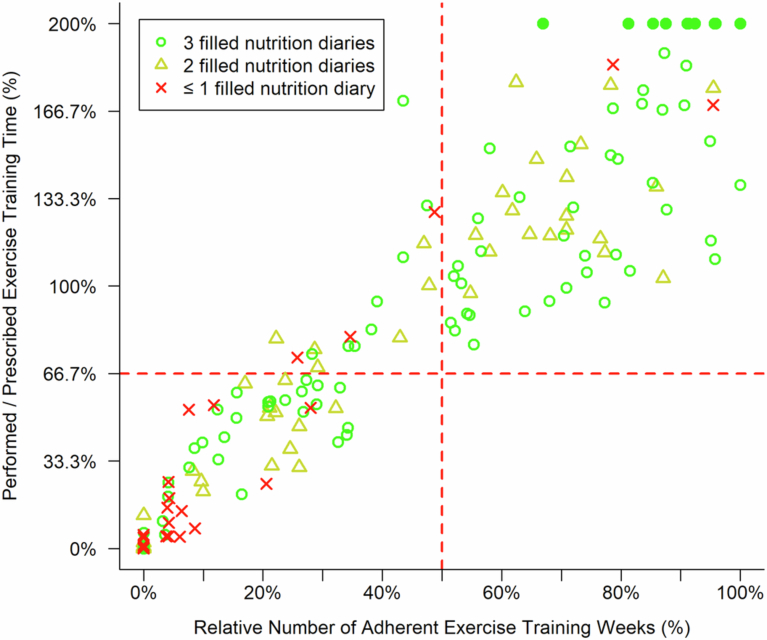
Adherence to the lifestyle intervention between baseline and 6-month follow-up. Adherence to nutritional counseling was defined as returning at least 2 of 3 nutrition diaries. Adherence to exercise training was defined as having performed at least 66.7% of prescribed training duration and reaching target duration in at least 50% of weeks. Green circles and yellow triangles (adherent to the diet intervention) in the upper right quadrant (adherent to the exercise training) show the lifestyle intervention patients who were adherent and included in the original per-protocol analysis (n = 76 patients (38%)). Patients who performed more than 200% of the prescribed training time are placed at 200% with solid symbols. Original values were at 201%, 203%, 207%, 216%, 259%, 286%, 298%, 301%, 309%, 310%, 323%, 441%, 498% and 667%.

During the first 6 months, patients randomized to lifestyle intervention (and completed evaluation at 6 months) recorded a median (IQR) exercise duration of 68 min per week (1–141). Of these, 81 patients (41%) fulfilled the adherence criteria for the exercise intervention, performing 172 min per week (126–218), whereas 59 patients did not reach the prescribed exercise duration in any week (equal to 49% of nonadherent patients that were not lost until the 6-month follow-up) and 49 patients recorded a total of <30 min of exercise until the 6-month follow-up. In contrast, 144 patients (72%) fulfilled the adherence criteria for the nutritional intervention. In combination, 76 patients (38%) were adherent to both exercise training and nutritional intervention and were included in the prespecified per-protocol analysis at 6 months (Extended Data Fig. 2). Between 6 and 12 months, the recorded overall exercise duration dropped to a median (IQR) of 41 min per week (0–141), whereas those patients who were adherent to the exercise program until the 6-month follow-up continued with 144 min per week (69–219).

### Sensitivity analyses

**Fig. 2:**
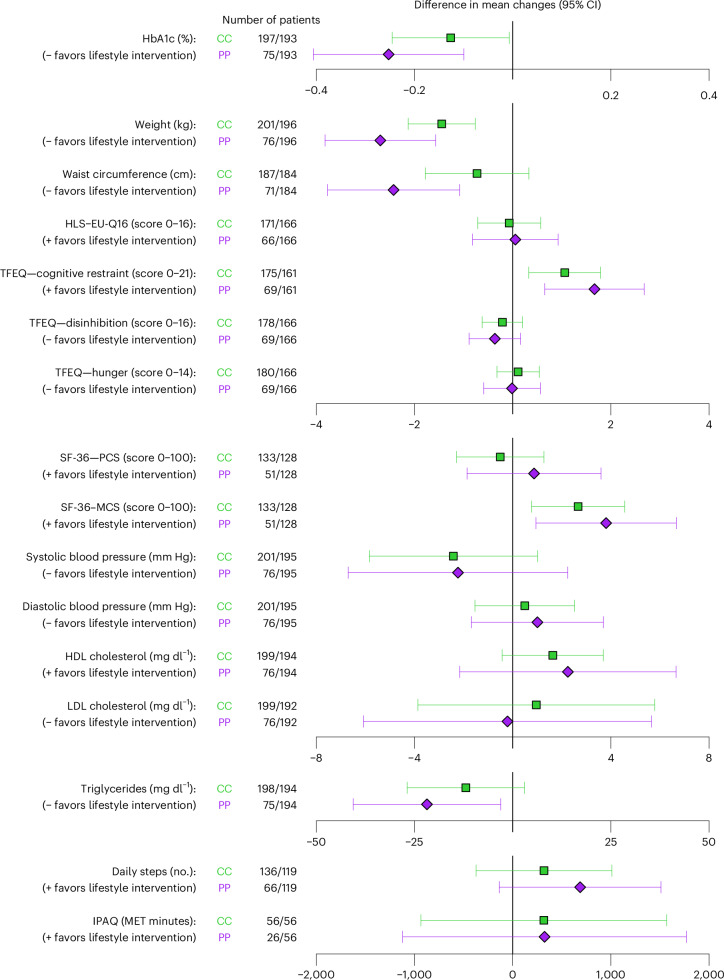
Results of the CC and PP analysis at 6 months. The CC analysis included all patients with available data at baseline and 6 months for the respective endpoint, whereas the PP analysis included all patients randomized to usual care and those patients randomized to the lifestyle intervention group who performed at least 66.7% of prescribed training duration, reached target duration in at least 50% of weeks and filled at least two of three nutrition diaries. Results indicate that for most of the investigated endpoints, favoring of lifestyle intervention over usual care is more pronounced in the PP analysis compared with the CC analysis. This highlights the potential of lifestyle interventions in patients with long-term T2DM and concomitant CHD. The number of patients refers to ‘*n* (lifestyle intervention)/*n* (usual care)’ for each endpoint and analysis set. CC, complete-case; MCS, mental component score; PCS, physical component score; PP, per-protocol.

In the per-protocol analysis at 6 months (Fig. 2 and Supplementary Table 2), there were significant differences between groups for HbA1c (difference in mean changes = −0.25% (95% CI, −0.41 to −0.10%), *P* = 0.002), body weight (−2.7 kg (−3.5 to −1.7), *P* < 0.001), waist circumference (−2.4 cm (−3.8 to −1.0), *P* < 0.001), triglycerides (−22 mg dl^−1^ (−42 to −2), *P* = 0.03), the QoL mental component score (3.8 (1.2–6.4), *P* = 0.004), the eating behavior score for ‘cognitive restraint of eating’ (1.7 (0.7–2.7), *P* = 0.001) and health literacy (1.0 (0.3–1.8), *P* = 0.007). After multiple imputation of missing values for the primary endpoint, change in HbA1c was not significantly different between groups (−0.12 ± 0.61% in the lifestyle intervention group and −0.06 ± 0.70% in the usual care group; mean difference = −0.06% (95% CI, −0.19 to 0.07), *P* = 0.35).

### Post hoc analyses

**Extended Data Fig. 3:**
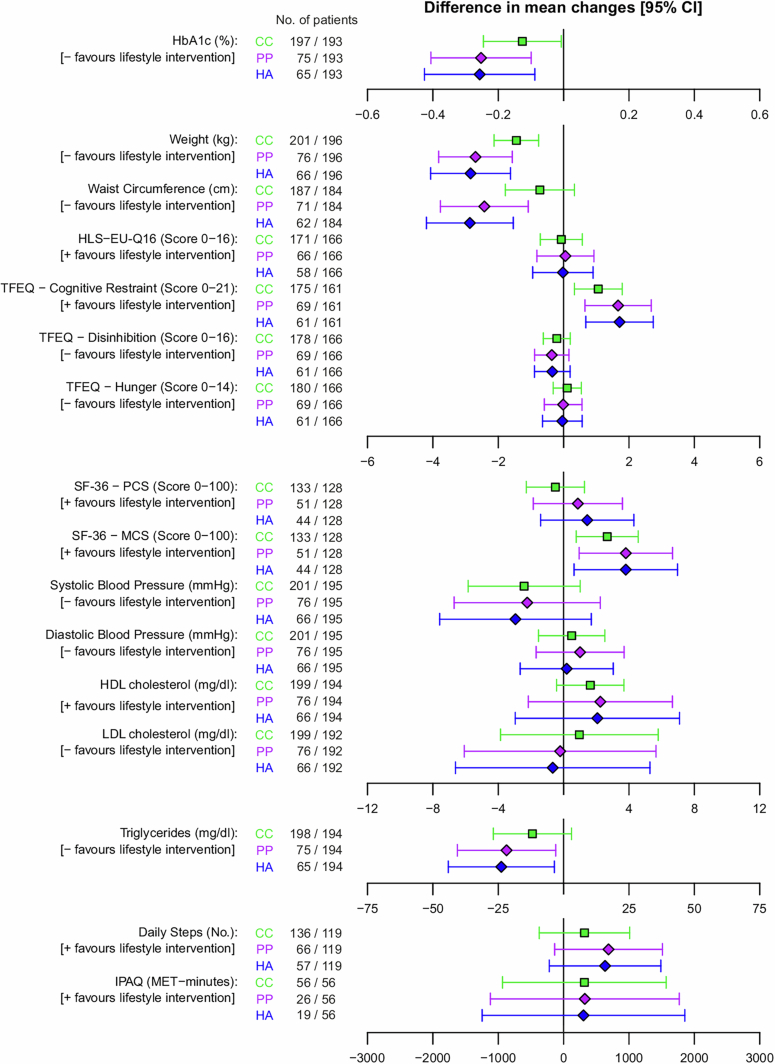
Comparison of the results from the complete-case (CC) analysis, the pre-specified per-protocol (PP) analysis and a modified per-protocol analysis with a higher adherence (HA) cutoff for exercise training. Adherence criteria for the original per-protocol (PP) analysis were performing ≥66.7% of the prescribed exercise training time, reaching the target duration in ≥50% of weeks and filling ≥2 of 3 nutrition diaries. In the first modified per-protocol analysis (high adherence, HA), the criterion of performed/prescribed exercise training time was increased to ≥100%. Number of patients refers to ‘n (lifestyle intervention)/n (usual care)’ for each endpoint and analysis set. CC, complete-case; HA, high adherence; HbA1c, glycated hemoglobin; HDL, high-density lipoprotein; HLS-EU-Q16, Health Literacy Survey European Questionnaire; IPAQ, International Physical Activity Questionnaire; LDL, low-density lipoprotein; MCS, mental component score; MET, metabolic equivalent of task; PCS, physical component score; PP, per-protocol; QoL, quality of life; SF-36, short form 36; TFEQ, Three-Factor Eating Questionnaire.

**Extended Data Fig. 4:**
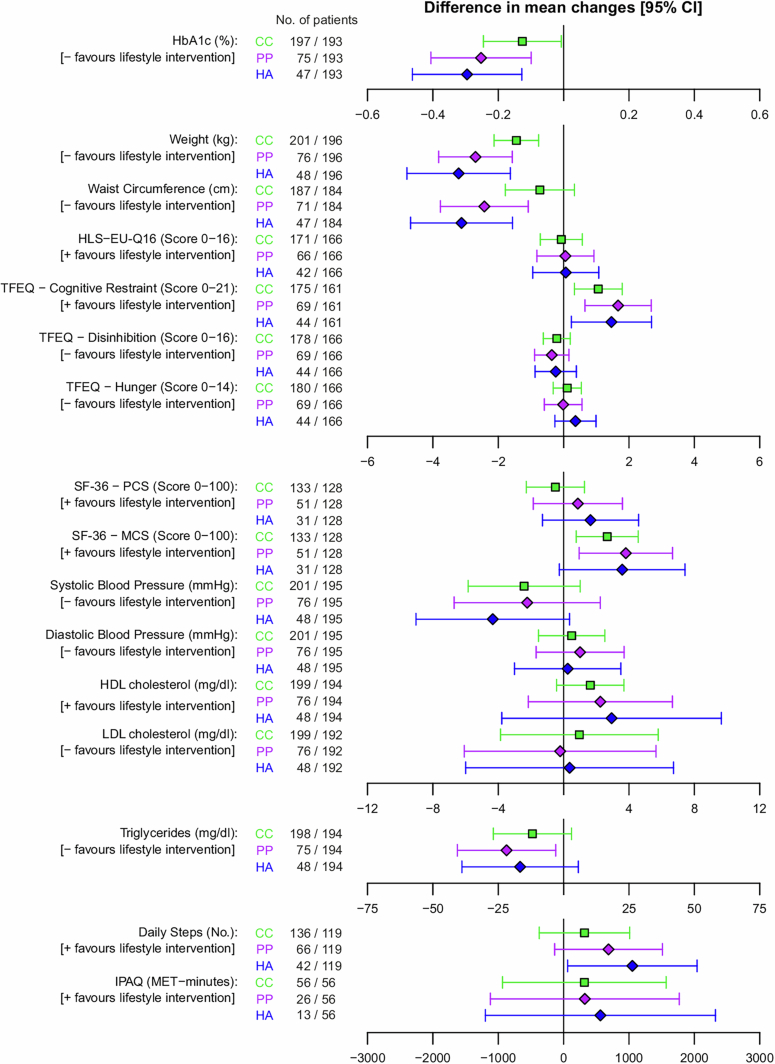
Comparison of the results from the complete-case (CC) analysis, the pre-specified per-protocol (PP) analysis and a modified per-protocol analysis with higher adherence (HA) cutoffs for the exercise and nutritional interventions. Adherence criteria for the original per-protocol (PP) analysis were performing ≥66.7% of the prescribed exercise training time, reaching the target duration in ≥50% of weeks and filling ≥2 of 3 nutrition diaries. In the second modified per-protocol analysis (high adherence, HA), the criteria for performed/prescribed exercise training time and filled nutrition diaries were increased to ≥100% and 3 of 3, respectively. CC, complete-case; HA, high adherence; HbA1c, glycated hemoglobin; HDL, high-density lipoprotein; HLS-EU-Q16, Health Literacy Survey European Questionnaire; IPAQ, International Physical Activity Questionnaire; LDL, low-density lipoprotein; MCS, mental component score; MET, metabolic equivalent of task; PCS, physical component score; PP, per-protocol; QoL, quality of life; SF-36, short form 36; TFEQ, Three-Factor Eating Questionnaire.

Based on more stringent adherence cutoffs for the exercise training intervention (patients who performed ≥100% of the prescribed exercise training time) or for the exercise training and the nutritional intervention (patients who performed ≥100% of the prescribed exercise training time and filled three of three nutrition diaries), 66 and 48 patients from the lifestyle intervention group were included in the post hoc modified per-protocol analyses, respectively. These analyses showed comparable improvements in the lifestyle intervention group for the primary and secondary endpoints compared to the original per-protocol analysis with a mean difference in change in HbA1c between groups of up to −0.30% (95% CI, −0.46 to −0.13; Extended Data Figs. 3 and 4).

## Discussion

In older patients with CHD and long-term T2DM, 6 months of telemedicine-supported, home-based, personalized exercise training combined with individual nutritional counseling and health literacy education led to a modest but statistically significant improvement in HbA1c. Patients who adhered closest to the intervention during the first 6 months had an average reduction in HbA1c of ~0.3% compared to usual care, a result that is generally considered clinically meaningful^21^ and was found to be consistent based on additional post hoc sensitivity analyses with modified adherence criteria. In addition, body weight also significantly decreased within 6 months, but no significant effects could be achieved on daily physical activity measured by the IPAQ or daily step counts. Moreover, no significant changes in other cardiovascular risk factors such as systolic or diastolic blood pressure, LDL and HDL cholesterol were observed. However, patients in the telemedicine-supported lifestyle intervention group displayed significant improvements regarding their QoL mental component score, the eating behavior score for ‘cognitive restraint of eating’ and health literacy. None of these changes were sustained over the subsequent 6 months, in which individualized feedback and health literacy training were discontinued while other tools for the telemedicine intervention (that is, smartphone applications, heart rate sensors and pedometers) were maintained.

In the complete sample, the effect on blood glucose control was not consistent (no significant between-group difference after multiple imputations of missing values) and less than observed in previous T2DM trials. Previous meta-analyses have shown that HbA1c can be significantly reduced by ‘traditional’ in-person or home-based exercise training or nutritional interventions without telemedicine (approximately −0.5% to −0.7%)^9,10,22^, but also telemedicine-supported interventions (approximately −0.3% to −0.5%)^23–26^. However, none of the previously conducted telemedicine trials in T2DM have used a comparable intervention, and particularly for patients with combined T2DM and CHD, the evidence is scarce. In a small study that included 23 patients with T2DM and CHD, an intensive lifestyle intervention with daily exercise sessions over 6 months, a hypocaloric heart-healthy diet of 1,500 kcal d^−1^ and optimized medication did not significantly reduce HbA1c compared to usual care after 6 months^14^. In another trial (*n* = 137 patients with CHD and T2DM; mean age of 63 years; mean HbA1c of 7.4%), 12 months of endurance and resistance training (2× supervised and 1× home-based per week) also did not significantly reduce HbA1c compared to usual care^15^. Similar observations were made in a nonrandomized trial (*n* = 127; mean age of 62 years), in which 2 years of home-based endurance and strength training (four to six sessions per week) did not induce significant improvements of HbA1c compared to usual care^16^. In contrast, a health literacy intervention using mobile text messaging (six messages per week on glucose monitoring, blood pressure control, medication adherence, physical activity and lifestyle) in 502 patients with CHD and T2DM has shown a significant reduction in HbA1c by −0.3% compared to a control group^27^.

The mean change in HbA1c observed in our LeIKD study at 6 months was not superior to these trials, although we applied an intensive telemedicine-supported intervention with individualized exercise training prescriptions based on a combination of ventilatory thresholds and peak oxygen consumption^19^, a ‘shared decision-making’ approach incorporating patient preferences and additional nutritional counseling and health literacy education, all strategies that are viewed as key factors when prescribing successful lifestyle interventions^5,28^. Factors that may have had an influence on the change in HbA1c during the trial include the long duration of T2DM and the well-controlled HbA1c values of participants in the LeIKD trial. With an average of 12 years before randomization, the diabetes duration of participants was longer than in most previous lifestyle intervention trials in T2DM. In a subanalysis of the Look AHEAD trial, a longer history of T2DM was significantly associated with lower diabetes remission rates^29^. Moreover, despite the long duration of T2DM, patients participating in the LeIKD trial were already well treated with a mean baseline HbA1c of 6.9% (refs. ^30,31^), which may have limited the potential for further HbA1c reductions. Additionally, patients with more severe symptoms and lower fitness (who could potentially benefit the most from a lifestyle intervention trial) were more likely to dropout from the study. In fact, patients who were lost to follow-up at 6 months had stronger symptoms of angina and lower peak V̇O_2_ than those who continued the trial.

While telemedicine has been proposed as a promising strategy to incorporate the benefits of home-based and supervised interventions and increase adherence to lifestyle interventions^32^, this could not be confirmed in the present trial as the effects of the complete-case analysis were of minor clinical relevance for the primary and all secondary endpoints. Despite a multimodal and highly intensive telemedical approach involving several staff members in the core telemedicine facility, overall adherence to exercise was poor in this population of elderly patients with CHD and T2DM. Only 41% fulfilled the previously defined adherence criteria for the exercise intervention within the first 6 months. Almost half of the nonadherent patients did not achieve the prescribed exercise duration even in one single week of the intervention. Adherence to the nutritional intervention seemed to be higher (72% filled ≥2/3 nutrition diaries at 6 months) than for exercise training, which may have been the reason for a significant mean reduction of body weight of −2.2 kg after 6 months.

Questionnaire data have revealed that >2/3 of patients found it ‘rather difficult’ to handle technical devices. Therefore, numerous patients may have been overwhelmed by the demands of initiating lifestyle changes at home using multiple telemedical devices, despite repeated and continuous individual instructions and feedback. It has to be questioned whether an even more intensively supported home-based phase may overcome these initial barriers of exercise initiation and smartphone use. Our data rather indicate that in an older patient population with CHD and a long history of T2DM, an exclusively home-based lifestyle intervention with telemedical support seems to have clear limitations and may in this setting not be a practical, clinically effective or cost-effective approach for general application. However, as the per-protocol analyses showed more favorable improvements for the lifestyle intervention group, increasing adherence to lifestyle intervention trials should be a major focus of future research. An alternative approach with a preceding on-site supervised phase may allow one to overcome initial barriers to exercise participation (for example, uncertainty, fear of adverse events and low motivation) before gradually transitioning to the home-based intervention. As the positive effects observed at 6 months diminished after a subsequent 6 months, when individualized feedback and health literacy training were discontinued (while other telemedicine tools such as the smartphone application, heart rate monitor and pedometer were maintained), this supports our previous finding that even in telemedical lifestyle interventions, personal contact (face-to-face or via telephone) is a central feature^33^.

Although we observed a numerically higher number of 4P-MACE in the lifestyle intervention group (20 versus 11 events), it is unlikely that this difference was causally related to the exercise intervention, because most events occurred in patients who were nonadherent to the exercise intervention. Moreover, despite concurrent T2DM, the overall number of events was substantially lower compared with other exercise trials in CHD^8^. In contrast, 70% of lifestyle intervention patients with a 4P-MACE fulfilled the adherence criteria for the nutritional intervention and had an average body weight loss of −1.8 kg between baseline and the last record before the event. Similar results have been observed in the Look AHEAD trial. Patients with T2DM and cardiovascular disease at baseline who were randomized to the weight loss program also had a numerically higher rate of 4P-MACE during a median follow-up of 9.6 years^11^. Therefore, clinical events should be closely monitored in future lifestyle or weight loss intervention trials in patients with T2DM and concurrent cardiovascular disease. In addition, special attention should be paid to preventing the loss of skeletal muscle mass, as this may be the key factor for the cardiovascular benefits of weight loss in patients with T2DM^34^.

The present study has several limitations. Foremost, the COVID-19 pandemic had a significant influence on the original study design impairing patient recruitment, forcing intermediate closures of study sites and sports facilities and other legal restrictions. This led to reduced recruitment, deviations from the original visit plan, an increased dropout rate and likely also reduced adherence to the intervention. Moreover, the pandemic influenced the patients´ general mobility and daily physical activity. The number of included patients was lower than initially planned (502 versus 750 for the primary endpoint), which reduced the statistical power to detect a difference in change in HbA1c between groups (Extended Data Table 2). Although we were able to detect a significant difference between groups for the primary endpoint (in the complete-case analysis), the reduced sample size may partly explain why the observed difference in HbA1c was not consistent (that is, no significant difference in the imputation set). Moreover, it also reduced the power to detect differences in the subgroup analyses.

Another limitation was that the study design did not allow differentiation of the effects associated with the different components of the intervention and the telemedicine features. Regarding the patient population, we had expected to include more patients with poorly controlled diabetes at baseline, but ~2/3 of patients had baseline HbA1c levels <7.0%, limiting the potential for improvement with lifestyle intervention. Female participation was low, which is comparable to the only two other randomized controlled trials on exercise training in patients with CHD and T2DM (16% and 22% women)^14,15^ and confirms the overall significant underrepresentation of women in clinical trials including patients with CHD^35^. Moreover, 98% of the participants were white, which also limits the generalizability of our findings.

It could be argued that the provision of pedometers and glucose monitors to the usual care group may be considered an intervention in itself. However, as we were primarily interested in the effects of the exercise training, nutrition and health literacy recommendations, providing these wearables to both groups allowed the elimination of the potential effects associated with the provision itself and also allowed the evaluation of changes in daily physical activity. Finally, patients and investigators were not blinded to treatment group assignment; however, this is a general limitation in lifestyle intervention trials.

In conclusion, an exclusively home-based lifestyle intervention program with telemedicine support and individualized remote counseling is difficult to implement and has minor beneficial clinical effects in older patients with CHD and a long history of T2DM. When individualized feedback is discontinued, the effects of a telemedicine-supported lifestyle intervention are not superior to usual care.

## Methods

### Study design and participants

LeIKD (ClinicalTrials.gov registration: NCT03835923) is a multicenter, randomized, clinical trial with two parallel groups conducted at 11 study sites in Germany (Munich, two sites in Berlin, Aachen, Magdeburg, Dresden, Leipzig, Kassel, Greifswald, Freiburg and Villingen-Schwenningen). All study participants were insured by one health insurance fund (Techniker Krankenkasse), which selected patients from their database according to the following criteria:

- Diagnosis of CHD (International Classification of Diseases, Tenth Revision (ICD-10) code: I20–I25) and T2DM (ICD-10 code: E11; ICD-10 codes must have been reported at least two times to reduce the likelihood of false positive coding).
- Enrollment in a disease management program for CHD and/or T2DM (including regular medical visits and offers for educational programs financed by the health insurance fund).
- Living within 50 km of a participating study site.
- Age ≥18 years.

Moreover, patients must have fulfilled none of the following criteria:

- Mental and behavioral disorders (ICD-10 codes: F00, F01, F02, F11, F12, F13, F14, F15, F16, F18, F20, F21, F22, F23, F24, F25, F28, F29, F44, F72, F73, F17 and F84).
- Heart failure New York Heart Association (NYHA) class IV (ICD-10 code: I50.14).
- Malignant neoplasm (ICD-10 codes: C25, C34, C56, C72, C73, C78, C79 and C97).
- Parkinson’s disease (ICD-10 code: G20).
- Alzheimer’s disease (ICD-10 code: G30).
- Infantile cerebral palsy (ICD-10 code: G80).
- Chronic kidney disease (ICD-10 codes: N18.4 and N18.5).
- Trisomy 21 (ICD-10 code: Q90).
- Blindness/visual impairment (ICD-10 codes: H54.0, H54.2 and H54.3).
- Hearing loss (ICD-10 codes: H90.0, H90.3, H90.5, H90.6 and H90.8).
- Care level 1–5.
- Insured abroad.

Patients who fulfilled the aforementioned criteria were contacted via telephone by an employee of the health insurance fund and informed about the possibility of getting screened for participation in the present trial. Contact details of interested patients were then forwarded to the study core lab in Munich and from there to the respective local study site to schedule the on-site screenings. During the on-site screening, patients were informed about all details of the study, provided their written informed consent and had to fulfill the following additional inclusion criteria:

- HbA1c ≥ 6.5% or antidiabetic medication at the time of the on-site screening.
- Permission to perform exercise training by the study physician.

Moreover, patients must have fulfilled none of the following criteria during on-site screening:

- Inability to do physical exercises or conditions that may interfere with exercise intervention.
- No optimal cardiac treatment within the last 4 weeks.
- Not clinically stable within the last 4 weeks.
- Participation in another clinical trial.

### Randomization

Eligible patients were randomly assigned (1:1) to lifestyle intervention or usual care using a web-based system (secuTrial, interActive Systems GmbH) stratified by study site with block sizes of four. Patients and staff were not blinded to treatment group assignments.

### Ethics approval

The original study protocol was approved by the ethics committee of the Technical University of Munich on 7 May 2018 (reference: 144/18 S) and the local ethics committees at the Universities of Berlin, Aachen, Magdeburg, Dresden, Leipzig, Greifswald and Freiburg, and the ethics committees of the Medical Associations of Hesse and Baden-Württemberg. A revised trial protocol (including a refinement of the inclusion criterion for the diagnosis of T2DM, that is, ‘HbA1c ≥ 6.5% or antidiabetic medication at the time of on-site screening’ in addition to ‘ICD-10: E11’) had also been approved by the ethics committees of the Technical University of Munich (reference: 144/18 S-AS) and the participating sites. All participants provided written informed consent. Detailed descriptions of the study design^37^ and baseline characteristics of the participants^31^ have been published previously and the study protocols, and statistical analysis plan can be found in the Supplementary lnformation.

### Interventions

Each patient who was randomized to the lifestyle intervention group received an individualized exercise schedule including endurance (primarily walking and/or cycling, depending on patient preference) and strength training with the aim to progressively increase training duration to ≥150 min per week^37^. Endurance training intensities were based on ventilatory thresholds and peak oxygen consumption derived from cardiopulmonary exercise testing (CPET) and varied between low and moderate-to-vigorous intensities, including continuous and interval sessions (excluding high-intensity interval training). Strength training consisted of several exercises targeting different muscle groups, and each patient received a training booklet with figures, detailed instructions and multiple possible variations of each exercise, as well as access to a website with videos for each exercise. The individualized exercise training plan was provided via the LeIKD smartphone application (IDS Diagnostic Systems AG), which was also used to record training duration and intensity using a heart rate sensor (Polar H7, Polar Electro GmbH). These recordings provided the basis for regular individualized telephone feedback sessions. Patients were contacted in the first training week (third week after randomization), 2 weeks thereafter and then every 4 weeks to discuss their progress and potential adaptations to the exercise training plan. After 6 months, training intensities were adjusted based on the results of the repeated CPET, and patients received a final call to discuss their exercise training plan for the upcoming 6 months without further feedback.

The nutritional recommendations were based on 7-day nutrition diaries (including food and beverages), which patients had to complete in weeks 1, 5 and 14 after randomization, as well in week 1 after their 6-month follow-up. The diaries were evaluated based on the ‘energy density principle’, and each patient received individualized written feedback, including recommendations on how to improve eating patterns and the composition of their diet. Moreover, to increase health literacy and motivation, lifestyle intervention participants received e-mails twice weekly for 6 months with tips and tricks on how to increase daily physical activity and information on a well-balanced diet, including recipe recommendations.

The entire lifestyle intervention was centrally provided and communicated by the core study center in Munich. This included analysis of all CPET raw data, exercise recordings and nutrition diaries, sending e-mail newsletters and providing individualized telephone and written feedback on the exercise and diet intervention and adaptations.

In contrast, patients randomized to usual care did not receive active intervention but received one-time written standard recommendations on nutrition and physical activity according to current guidelines^37^. However, irrespective of group allocation, all patients received a pedometer (Beurer GmbH, AS80/AS87) and a blood glucose meter (Beurer GmbH, GL50evo) to monitor daily physical activity and blood glucose levels. These wearables were connected to a smartphone application (Beurer Health Manager; Beurer GmbH). If necessary, patients in either group received a smartphone for the duration of the study.

### Conceptual framework of the intervention

The initial idea for the LeIKD trial stems from a healthcare program that is carried out in cooperation between the TUM University Hospital and the largest German health insurance fund, ‘Techniker Krankenkasse’ (Supplementary Table 1). Since 2011, more than 600 patients with CHD and/or T2DM have been included in this ongoing clinical program, which consists of a combination of supervised and home-based endurance and resistance training, nutritional counseling and coping and behavioral change strategies over 6 months. An analysis from a subsample of these patients has been published previously^38^.

The rationale of the LeIKD trial was to extend and transform this healthcare program to be broadly applicable. Given the funding by the Federal Joint Committee that decides which medical services are reimbursed by the statutory health insurance funds, the intervention was designed to be transferable into standard German healthcare. As on-site programs are neither sustainable nor cost-efficient in the long term, the program was designed as a home-based intervention with telemedical support (including individualized feedback from experts). The LeIKD smartphone application was adapted from an existing commercial application (mysportsapp; IDS Diagnostics Systems AG) that had been developed in cooperation with patients and experts at the TUM University Hospital and had already been applied in routine care for patients with cardiovascular diseases.

The intervention concept of the present study was further developed based on experiences from previously conducted large-scale exercise training trials in heart failure^39–41^, including one with telemedical support for the exercise training intervention^40^, as well as extensive institutional experience in prescribing exercise training and providing nutritional counseling for patients with metabolic and/or cardiovascular diseases in the routine care setting.

The exercise training program of the present trial started with a relatively high exercise training frequency (up to six times per week) and low session duration (as low as 10 min per session) to integrate regular exercise into everyday life. In adherence to the general exercise training principles of individualization and overload (including the secondary principles of variation and progression)^42^, the volume has then been gradually increased over time to reach at least 150 min per week. From the beginning, patients were regularly involved in the decision-making process on how the exercise plan could be adapted to better meet their individual needs. The high exercise frequency, the regular feedback, the shared decision-making and the additional health literacy training were also intended to improve self-efficacy, self-concordance, implementation intentions and volitional strategies to protect the behavior change against external and internal barriers, which are important elements in behavior change theories^43^.

The rationale of the nutritional counseling focusing on body weight loss in obese patients and overall dietary improvements was based on the energy density principle. This method has been shown to successfully reduce body weight with an individually tailored change of eating habits, has low barriers (as it does not prohibit any food) and is easily applicable and sustainable for patients with T2DM^44^. The energy density principle has also been frequently applied in the routine care of the coordinating study site and our aforementioned healthcare project in patients with CHD and/or T2DM^38^. Moreover, self-monitoring of dietary behavior and receiving regular feedback are considered behavior change techniques that are frequently applied in digital behavior change interventions^45,46^. Similar to the exercise intervention, the nutritional intervention has also been supported by additional health literacy information and recipe suggestions to promote a healthy and well-balanced diet.

### Examinations and endpoints

All participants were assessed at baseline, 6 months after randomization and another 6 months after the first follow-up (12 months after baseline). Examinations included medical history, physical examination, anthropometry, electrocardiography, blood draw with local laboratory analysis, CPET and questionnaires to assess health literacy (European Health Literacy Questionnaire, HLS-EU-Q16), QoL (Short Form Health Survey, SF-36), daily physical activity (IPAQ) and eating behavior (Three-Factor Eating Questionnaire (TEFQ)).

Adverse events were documented by the local investigators and forwarded to the study core lab in Munich. Each adverse event form was reviewed by one physician blinded to treatment group assignment. If necessary, additional information (for example, hospital discharge letters) was requested from the local study sites. A serious adverse event was defined as any adverse event that resulted in death, was life-threatening, required hospitalization or prolongation of an existing hospitalization, resulted in persistent or significant disability or incapacity or required medical or surgical intervention to prevent a life-threatening condition, persistent or significant disability or incapacity. Major adverse cardiovascular events (4P-MACE) were defined as hospitalization due to angina/revascularization, nonfatal myocardial infarction, nonfatal stroke or cardiovascular death.

Primary endpoint was the change in HbA1c (%) after 6 months (analyzed in the local laboratories). Secondary endpoints included the following cardiovascular risk factors, QoL, health literacy, eating behavior, daily physical activity and safety parameters:

- Change in HbA1c after 12 months (analyzed in the local laboratories).
- Change in HDL cholesterol after 6 and 12 months (analyzed in the local laboratories).
- Change in LDL cholesterol after 6 and 12 months (analyzed in the local laboratories).
- Change in triglycerides after 6 and 12 months (analyzed in the local laboratories).
- Change in body weight after 6 and 12 months (with validated weight standing scales).
- Change in waist circumference after 6 and 12 months (with measuring tape).
- Change in systolic blood pressure after 6 and 12 months (with validated blood pressure monitors, on the left upper arm after 5 min of rest).
- Change in diastolic blood pressure after 6 and 12 months (with validated blood pressure monitors, on the left upper arm after 5 min of rest).
- Change in health literacy after 6 and 12 months (HLS-EU-Q16 questionnaire; score range = 0–16 (higher scores reflect better health literacy)).
- Change in QoL (physical component score) after 6 and 12 months (SF-36 questionnaire; score range = 0–100 (higher scores reflect better QoL)).
- Change in QoL (mental component score) after 6 and 12 months (SF-36 questionnaire; score range = 0–100 (higher scores reflect better QoL)).
- Change in eating behavior (cognitive restraint of eating) after 6 and 12 months (TFEQ; score range = 0–21 (higher scores reflect better cognitive restraint)).
- Change in eating behavior (disinhibition) after 6 and 12 months (TEFQ; score range = 0–16 (higher scores reflect worse control)).
- Change in eating behavior (hunger) after 6 and 12 months (TEFQ; score range = 0–14 (higher scores reflect higher susceptibility for internal and external hunger signs)).
- Change in daily physical activity after 6 and 12 months (IPAQ).
- Change in average steps per day over 7 days after 6 and 12 months (with pedometers).
- Number of patients with 4P-MACE (including cardiovascular death, nonfatal stroke, nonfatal myocardial infarction and hospitalization due to angina or revascularization) after 6 and 12 months.

Adherence to the exercise intervention was measured using the exercise training recordings via the smartphone application and cross-validated by using the documentation of the regular feedback sessions. Adherence to the exercise intervention is reported as both median (IQR) minutes per week and the ratio between documented and prescribed exercise training time. Adherence to the nutritional intervention was measured by the ratio of completed to prescribed nutrition diaries. The additional secondary endpoints of changes in medical care expenses from baseline to 6 and 12 months are not reported in this manuscript and will be published separately. Likewise, the prespecified cost-effectiveness analysis will be published together with the medical care expenses.

### Sample size calculation

For initial sample size calculation, a between-group difference in HbA1c of 0.4% with an s.d. of 1.8% (effect size *d* = 0.222) was assumed based on the results of the ‘Enhancing Adherence in Type 2 Diabetes (ENHANCE)’ trial^47^. With a power of 80%, a two-sided significance level of *α* = 0.05 and an estimated dropout rate of 15%, a total of 750 patients would have been required to detect a significant difference in the primary endpoint^37^. However, administrative delays, insufficient recruitment rates at several study sites and a fixed project duration required adjustments to the sample size calculation during the conduct of the study^31^. Based on a higher effect size of *d* = 0.305 with an estimated dropout rate of 30%, we aimed to include at least 486 patients^31^.

### Treatment of missing data and loss to follow-up

To account for missing values of the primary endpoint, a multiple imputation approach has been applied. Imputation was performed using predictive mean matching (implemented in the R package ‘mice’)^48^ under consideration of the baseline parameters age, sex, BMI, HbA1c, insulin (yes/no), other antidiabetic medication (yes/no), CHD classification (≤1 vessel disease versus ≥2 vessel disease), education level (low/medium versus high), usage of mobile applications (daily versus less than daily) and randomization group. Ten datasets with imputed values were generated and pooled to test the null hypothesis of equal change in HbA1c for both groups.

### Statistical analysis

All endpoints except for the number of patients with 4P-MACE were evaluated with *t* tests for independent samples, whereas the number of patients with 4P-MACE was evaluated with the *χ*^2^ test. All patients were analyzed in the group they were randomized to. For the main analyses, only patients with complete paired baseline and follow-up measurements were included. However, the analysis of the primary endpoint (change in HbA1c at 6 months) was repeated after multiple imputations of missing values as specified in the statistical analysis plan and described above. For the prespecified per-protocol analysis, patients of the lifestyle intervention group were excluded from the analysis when they had completed <66.7% of the planned overall exercise duration or achieved the planned weekly exercise duration in <50% of weeks or completed <2/3 of the nutrition diaries until the 6-month follow-up. To evaluate the robustness of the results from the per-protocol analysis, two additional post hoc analyses were performed. First, the criterion for performed/prescribed exercise training time was increased to ≥100%. Second, the criteria for performed/prescribed exercise training time and filled nutrition diaries were increased to ≥100% and three of three nutrition diaries. To confirm the robustness of the findings from the per-protocol analysis, the point estimates for the primary and secondary endpoints of these post hoc analyses should be comparable to or more favorable than in the original per-protocol analysis. Moreover, the analysis of the primary endpoint was repeated within prespecified subgroups by fitting linear regression models with tests for interaction between these variables and study groups to the data. All statistical tests were performed using R Statistical Software (v.4.0.2, R Foundation for Statistical Computing) with two-sided significance levels of *α* = 0.05. As CIs have not been adjusted for multiple testing, analyses of secondary endpoints should be interpreted as exploratory.

### Reporting summary

Further information on research design is available in the Nature Portfolio Reporting Summary linked to this article.

## Online content

Any methods, additional references, Nature Portfolio reporting summaries, source data, extended data, supplementary information, acknowledgements, peer review information; details of author contributions and competing interests; and statements of data and code availability are available at 10.1038/s41591-025-03498-w.

## Supplementary information

Supplementary Information Supplementary Tables 1 and 2, Supplementary Fig. 1 and Supplementary Data (study protocol—trial protocol versions 1.0 and 1.1 and statistical analysis plan).

Reporting Summary

## Figures and Tables

**Extended Data Table 1:**
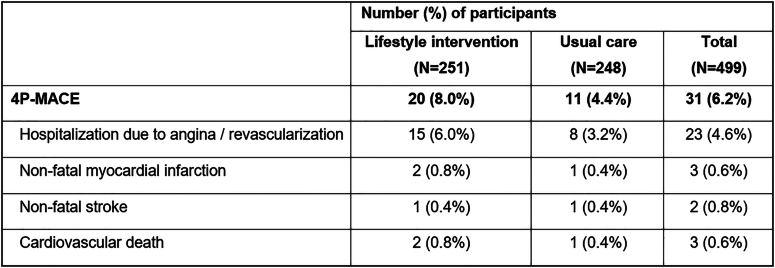
Number of participants experiencing a major adverse cardiovascular event (4P-MACE) between baseline and 12-month follow-up

**Extended Data Table 2:**
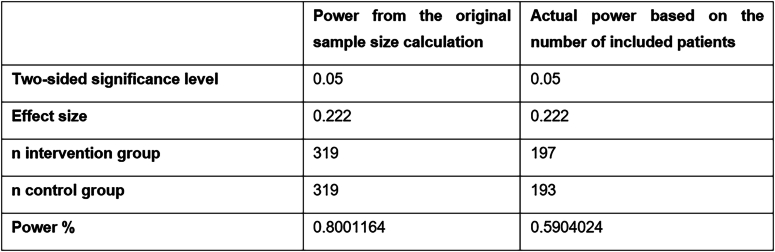
Comparison between power based on the assumptions applied in the initial sample size calculation and the number of patients included in the analysis of the primary endpoint

The calculated power is based on a two-sided significance level of 0.05, the initially assumed effect size of *d* = 0.222 and the numbers of patients per group. For the initial power (left column), this is the required number to be included in the analysis of the primary endpoint (neglecting the assumed rate of missing values). For the actual power (right column), this is the number of patients that were actually included in the analysis of the primary endpoint.

## Data Availability

As patients have not explicitly consented to sharing pseudonymized data, we are not allowed to share the individual participant data for legal reasons. However, upon request to the corresponding author (M.H.), aggregated data (data on demographics, clinical history, pharmacological treatment, electrocardiography, laboratory parameters, questionnaire data and CPET data) that does not allow identification of individual patients may be shared after consultation with the data protection officers and legal representatives of the participating institutions and after signing a data sharing agreement. A response to requests for data access can be expected within 4 weeks.
