# Supplementary material for: Telemedicine-supported lifestyle intervention for glycemic control in patients with CHD and T2DM: multicenter, randomized controlled trial
Source: Nat Med. 2025 Feb 7;31(4):1203–13. doi: 10.1038/s41591-025-03498-w (PMC12003154; doi:10.1038/s41591-025-03498-w)
Supplement: Supplementary file 1 — Supplementary Tables 1 and 2, Supplementary Fig. 1 and Supplementary Data (study protocol—trial protocol versions 1.0 and 1.1 and statistical analysis plan). [file 41591_2025_3498_MOESM1_ESM.pdf]

nature medicine

Supplementary information https://doi.org/10.1038/s41591-025-03498-w

Telemedicine-supported lifestyle intervention for glycemic control in patients with CHD and T2DM: multicenter, randomized controlled trial

In the format provided by the authors and unedited

Supplementary Information

Table of Contents

Supplementary Table 1: Comparison of baseline characteristics between patients who continued until 6-month follow-up (primary endpoint) and those who were lost to follow-up 2

Supplementary Table 2: Results of the primary and secondary endpoints within the per-protocol analysis after 6 months..4

Supplementary Figure 1: Conceptual framework of the LeIKD intervention 6

Trial Protocol Version 1.0 7

Trial Protocol Version 1.1 28

Statistical Analysis Plan 49

Supplementary Table 1: Comparison of baseline characteristics between patients who continued until 6-month follow-up (primary endpoint) and those who were lost to follow-up

	**No Dropouts until 6-month follow-up** N = 408	**Dropouts until 6-month follow-up** N = 91	**p-value**
Sex			0.184
Male	346 (84.8%)	72 (79.1%)	
Female	62 (15.2%)	19 (20.9%)	
Age at inclusion, years	68.2 (7.7)	68.9 (7.8)	0.394
Level of education [a]			0.007
Low	11 (2.9%)	1 (1.2%)	
Medium	166 (43.2%)	52 (61.9%)	
High	207 (53.9%)	31 (36.9%)	
Body mass index, kg/m^2	30.1 (4.9)	30.1 (4.5)	0.858
Resting heart rate, beats/min	69.2 (11.2)	71.0 (11.1)	0.120
Blood pressure, mmHg			
Systolic	137.3 (17.3)	138.1 (17.6)	0.678
Diastolic	78.6 (9.9)	79.8 (10.1)	0.183
**Coronary heart disease and type 2 diabetes**			
Duration of CHD, years	8.5 (7.1)	9.9 (8.2)	0.205
CHD classification			0.508
No relevant stenosis (< 50%)	73 (17.9%)	14 (15.4%)	
1-vessel disease	92 (22.5%)	23 (25.3%)	
2-vessel disease	74 (18.1%)	10 (11.0%)	
3-vessel disease	108 (26.5%)	30 (33.0%)	
Left main coronary disease	7 (1.7%)	2 (2.2%)	
Unknown	53 (13.2%)	12 (13.2%)	
CCS-score			0.039
Grade 0	337 (82.8%)	67 (73.6%)	
Grade I	57 (14.0%)	16 (17.6%)	
Grade II	11 (2.7%)	8 (8.8%)	
Grade III-IV	2 (0.5%)	0 (0.0%)	
Previous myocardial infarction	133 (32.6%)	38 (41.8%)	0.087
Coronary revascularization	219 (53.7%)	55 (60.4%)	0.241
Coronary artery bypass graft	63 (15.4%)	19 (20.9%)	0.206
Duration of type 2 diabetes, years	12.3 (8.4)	11.9 (8.4)	0.664
HbA1c			0.327
%	6.8 (0.9)	7.1 (1.2)	
mmol/mol	50.9 (9.3)	53.6 (13.3)	
Number of oral anti-diabetic agents			0.519
0	64 (15.7%)	21 (23.1%)	
1	170 (41.7%)	35 (38.5%)	
2	131 (32.1%)	26 (28.6%)	
≥ 3	43 (10.5%)	9 (9.9%)	
Patients treated with insulin	105 (25.7%)	32 (35.2%)	0.059
**Other cardiovascular risk factors and diseases**			
Hypertension	374 (91.7%)	87 (95.6%)	0.246
Hyperlipidemia	349 (85.5%)	80 (87.9%)	0.525
Smoking			0.182
No (never smoked)	154 (37.7%)	31 (34.1%)	
Ex-smoker	214 (52.5%)	45 (49.5%)	
Current	40 (9.8%)	15 (16.5%)	
Heart Failure	100 (24.5%)	20 (22.0%)	0.661

	**No Dropouts until 6-month follow-up** N = 408	**Dropouts until 6-month follow-up** N = 91	**p-value**
Atrial Fibrillation	78 (19.1%)	18 (19.8%)	0.746
Peak Oxygen Consumption			
mL/kg/min	19.0 (4.6)	16.8 (4.2)	<0.001
% predicted norm values [b]	83.3 (16.6)	75.6 (16.5)	<0.001
Having an own mobile device	335 (87.9%)	65 (77.4%)	0.012
Usage of mobile applications			0.092
Daily	246 (64.6%)	48 (57.1%)	
Weekly	37 (9.7%)	5 (6.0%)	
Less than weekly / never	98 (25.7%)	31 (36.9%)	
Handling of technical devices			0.075
Rather easy	56 (14.9%)	21 (25.0%)	
Rather difficult	285 (75.6%)	55 (65.5%)	
Don't know	36 (9.5%)	8 (9.5%)	

Data are mean (SD) or n (%). P-values are based on two-sided Pearson's Chi-squared test, two-sided Wilcoxon rank sum test or two-sided Fisher's exact test as appropriate. P-values were not adjusted for multiple comparisons. P-values for difference in peak oxygen consumption (mL/kg/min and % predicted norm values) were $<2.2e^{-16}$. Abbreviations: CCS = Canadian Cardiovascular Society. HbA_{1c} = glycated hemoglobin.

[a] Level of education was based on the International Standard Classification of Education 2011 according to the highest self-reported graduation and/or professional training; [b] based on the normative data from the SHIP (Study of Health in Pomerania) study

Supplementary Table 2: Results of the primary and secondary endpoints within the per-protocol analysis after 6 months

	**Mean (SD) [N]**					
	**Lifestyle Intervention**		**Usual Care**		**Change from Baseline to 6 months**	
	**Visit**	**Change from Baseline**	**Visit**	**Change from Baseline**	**Mean Difference (95% CI) [N]**	**P-value**
**HbA_{1c} (%)**						
Baseline	6.80 (0.91) [75]	..	6.92 (0.97) [248]	..		
6 months	6.55 (0.73) [76]	-0.26 (0.56) [75]	6.78 (0.78) [193]	0.00 (0.590) [193]	-0.25 (-0.41 to -0.10) [268]	P=0.002
**Body Weight (kg)**						
Baseline	94.0 (17.6) [76]	..	91.4 (15.9) [248]	..		
6 months	90.6 (17.3) [76]	-3.5 (4.5) [76]	91.2 (15.5) [196]	-0.8 (3.2) [196]	-2.7 (-3.5 to -1.7) [272]	P<0.001
**Waist circumference (cm)**						
Baseline	109.0 (13.1) [72]	..	108.0 (11.9) [237]	..		
6 months	106.0 (13.2) [72]	-3.2 (4.7) [71]	107.5 (12.0) [192]	-0.7 (5.3) [184]	-2.4 (-3.8 to -1.0) [255]	P<0.001
**Systolic blood pressure (mmHg)**						
Baseline	138 (18) [76]	..	138 (18) [248]	..		
6 months	133 (16) [76]	-4 (16) [76]	135 (17) [195]	-2 (18) [195]	-2 (-7 to 3) [271]	P=0.36
**Diastolic blood pressure (mmHg)**						
Baseline	78 (9) [76]	..	80 (10) [248]	..		
6 months	78 (9) [76]	0 (10) [76]	78 (10) [195]	-1 (11) [195]	1 (-2 to 4) [271]	P=0.48
**HDL cholesterol (mg/dL)**						
Baseline	48 (16) [76]	..	47 (12) [248]	..		
6 months	50 (16) [76]	2 (19) [76]	46 (12) [194]	0 (8) [194]	2 (-1 to 5) [270]	P=0.17
**LDL cholesterol (mg/dL)**						
Baseline	87 (32) [76]	..	93 (32) [246]	..		
6 months	81 (30) [76]	-6 (20) [76]	87 (34) [194]	-6 (26) [192]	0 (-7 to 6) [268]	P=0.95
**Triglycerides (mg/dL)**						
Baseline	179 (91) [75]	..	188 (98) [248]	..		
6 months	147 (70) [76]	-32 (66) [75]	178 (109) [194]	-10 (79) [194]	-22 (-42 to -2) [269]	P=0.03

**Daily steps (no.)**						
Baseline	5990 (3170) [67]	..	5761 (3131) [151]	..		
6 months	6680 (3480) [71]	638 (2630) [66]	5833 (3668) [144]	-49 (2861) [119]	687 (-155 to 1530) [185]	P=0.11
**International Physical Activity Questionnaire (IPAQ) (MET minutes)**						
Baseline	3819 (2976) [34]	..	3173 (2869) [119]	..		
6 months	5613 (3550) [38]	1453 (2492) [26]	4240 (3568) [100]	1129 (3652) [56]	325 (-1365 to 2014) [78]	P=0.70
**Short Form 36 (SF-36) questionnaire – Physical Component Score [a]**						
Baseline	45.4 (10.9) [62]	..	45.1 (9.3) [191]	..		
6 months	48.6 (8.7) [59]	2.3 (9.0) [51]	46.9 (9.2) [163]	1.4 (6.2) [128]	0.9 (-1.4 to 3.2) [179]	P=0.46
**Short Form 36 (SF-36) questionnaire – Mental Component Score [a]**						
Baseline	52.7 (8.8) [62]	..	52.5 (9.0) [191]	..		
6 months	53.6 (7.2) [59]	2.2 (9.2) [51]	51.1 (9.3) [163]	-1.6 (7.3) [128]	3.8 (1.2 to 6.4) [179]	P=0.004
**European Health Literacy Survey Questionnaire (HLS-EU-Q16) [b]**						
Baseline	11.7 (3.5) [72]	..	12.6 (3.0) [227]	..		
6 months	12.6 (3.3) [68]	0.9 (2.8) [66]	12.5 (3.0) [177]	-0.1 (2.6) [166]	1.0 (0.3 to 1.8) [232]	P=0.007
**Three-Factor-Eating-Questionnaire (TFEQ) – Cognitive restraint [c]**						
Baseline	8.4 (4.5) [72]	..	7.8 (3.7) [225]	..		
6 months	10.0 (4.3) [71]	1.8 (3.6) [69]	7.6 (3.7) [176]	0.2 (3.5) [161]	1.7 (0.7 to 2.7) [230]	P=0.001
**Three-Factor-Eating-Questionnaire (TFEQ) – Disinhibition [d]**						
Baseline	4.7 (2.9) [72]	..	4.7 (3.0) [228]	..		
6 months	4.1 (2.6) [71]	-0.5 (1.8) [69]	4.5 (2.9) [178]	-0.1 (2.0) [166]	-0.4 (-0.9 to 0.2) [235]	P=0.19
**Three-Factor-Eating-Questionnaire (TFEQ) – Hunger [e]**						
Baseline	3.4 (2.7) [72]	..	3.9 (3.0) [228]	..		
6 months	2.9 (2.4) [71]	-0.6 (2.0) [69]	3.5 (3.0) [179]	-0.6 (2.0) [166]	0.0 (-0.6 to 0.6) [235]	P=0.96

All outcomes presented in this table were evaluated with two-sided t-tests for independent samples and were not adjusted for multiple comparisons. The exact p-values for the differences in change in weight and waist circumference were $6.576e^{-6}$ and 0.000489, respectively. Abbreviations: HbA_{1c} = glycated hemoglobin. HDL = high-density lipoprotein. LDL = low-density lipoprotein. MET = Metabolic equivalent of task.

[a] higher scores indicate better quality of life (score range, 0-100); [b] higher scores indicate better health literacy (score range, 0-16); [c] higher scores indicate better cognitive restraint (score, range 0-21); [d] higher scores indicate worse control (score, range 0-16); [e] higher scores indicate higher susceptibility for internal and external hunger signs (score, range 0-14)

Supplementary Figure 1: Conceptual framework of the LeIKD intervention

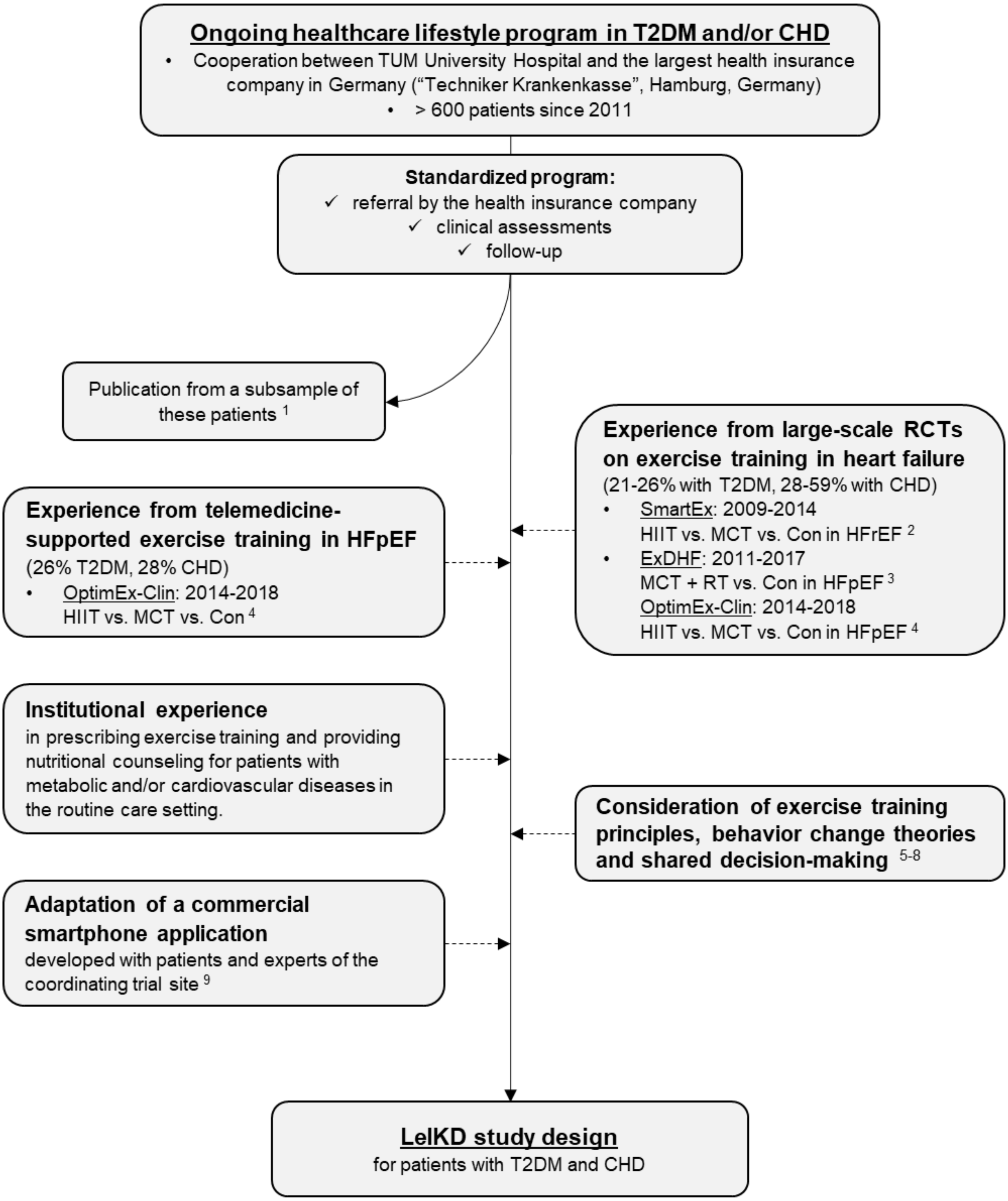

[1] J Cardiopulm Rehabil Prev. 2020; 40(1): 17-23.
[2] Circulation. 2017; 135(9): 839-849.
[3] Nat Med. 2025; 31(1): 306-314.
[4] JAMA. 2021; 325(6): 542-551.
[5] J Phys Act Health. 2011; 8(6): 794-803.
[6] Ann Behav Med. 2013, 46(1): 81-95.
[7] Front Digit Health. 2021, 3: 620383.
[8] ACSM's guidelines for exercise testing and prescription. Wolters Kluwer/Lippincott Williams & Wilkins; 2009.
[9] mysportsapp, IDS Diagnostics Systems AG, Ettlingen, GER.

Abbreviations: CHD = coronary heart disease; Con = control group; HIIT = high-intensity interval training; HFpEF = heart failure with preserved ejection fraction; HFrEF = heart failure with reduced ejection fraction; MCT = moderate continuous training; RCT = randomized controlled trial; TUM = Technical University of Munich; T2DM = Type 2 diabetes mellitus

Study protocol

Translation of the German Study Protocol

Version 1.0 (28.03.2018)

„Lifestyle Intervention in Chronic Ischemic Heart Disease and Diabetes”

(LeIKD)

Content

1. Project summary 3
2. Responsibilities 3
2.1. Principal investigator 3
2.2. Project leadership 4
2.3. Project partners 4
2.4. Funding 4
3. Scientific background 4
3.1. Exercise training as therapy 7
4. Study objectives 8
5. Outcome measures 10
6. Study design 10
7. Study population 11
7.1. Inclusion and exclusion criteria 11
8. Study procedures 12
9. Risk-benefit analysis 16
10. Biometrics 16
10.1. Descriptive analysis 16
10.2. Interferential statistical data analysis 16
10.3. Health economic analysis 17
11. Written informed consent 17
12. Data management and data protection 18
13. Biomaterial handling 19
14. Ethics committee 19
15. Legal basis 20
16. Subject insurance 20
17. Literature 20

1. Project summary

The majority of patients with chronic ischemic heart disease (CIHD) and diabetes mellitus type 2 (T2DM) is physically inactive and overweight. However, the successful implementation of an exercise and nutrition program in this population is not trivial as the experience from the past decades and previous projects has shown (e.g. "Sport als Therapie der Techniker Krankenkasse", a former lifestyle intervention project): expert knowledge is highly required to design individual tailored exercise and nutrition recommendations to achieve lifestyle changes in patients.

The “Lifestyle Intervention in Chronic Ischemic Heart Disease and Diabetes” (German title: **Le**bensstil- **I**ntervention bei **K**oronarer Herzkrankheit und **D**iabetes (LeIKD)) study addresses this point: The aim of the randomized controlled trial is to reduce cardiovascular risk factors and to increase health literacy in CIHD and T2DM patients by structured lifestyle intervention. The implementation of a home-based program with individual exercise and nutrition recommendations and supporting motivational strategies will take place in different regional areas in Germany. In addition to existing local medical care services, new telemedical approaches will also be applied like the developed LeIKD-exercise-training smartphone app. Therefore, a total of 1,500 patients with CIHD and TDM2 insured at the health insurance “Techniker Krankenkasse” (TK) will be included in the study. The primary endpoint is the reduction in glycated haemoglobin (HbA1c), secondary endpoints include changes in health literacy, quality of life, parameters of exercise capacity and dietary behaviour, and hospitalization rates.

The project is funded by the Innovation Fund and involies the following collaboration partners: Health insurance company “Techniker Krankenkasse” (Hamburg, Germany), Department of Prevention and Sports Medicine, University Hospital Klinikum rechts der Isar, Technical University of Munich (Munich, Germany), App-developer company IDS Diagnostic Systems AG (Karlsruhe, Germany) and the Institute for Applied Health Services Research inav (Berlin) as project evaluator.

2. Responsibilities

2.1. Principal investigator

Dr. med. A. Duvinage, Präventive und Rehabilitative Sportmedizin, Klinikum rechts der Isar, Technische Universität München. Ismaninger Str. 22 Bau 523, 81675 München.

2.2. Project leadership

Techniker Krankenkasse. M. Schultz. Bramfelder Straße 140. 22305 Hamburg

2.3. Project partners

Department of Prevention and Sports Medicine, University Hospital Klinikum rechts der Isar, Technical University of Munich, Univ.-Prof. Dr. med. M. Halle, Georg-Brauchle-Ring 56/58. 80992 Munich

Center for Prevention and Sports Medicine, University Hospital Klinikum rechts der Isar. Technical University Munich. Ismaninger Straße 22, Building 523. 81675 Munich

IDS Diagnostic Systems AG. Dr. med. B. Hackenberg. Karlsburgstraße 2. 76227 Karlsruhe

Privates Institut für angewandte Versorgungsforschung GmbH (inav). Univ.-Prof. Dr. V. Amelung. Schiffbauerdamm 12. 10117 Berlin.

2.4. Funding

Full funding is provided by the Federal Joint Committee (G-BA) (Germany); funding reference 01NVF17015.

3. Scientific background

CIHD and DM2 are among the most common causes of increased morbidity and mortality in Germany. Especially the combination of both diseases increases the risk exponentially [1]. The combined end point of death and myocardial infarction increased up to 30% after four years [2]. According to estimates by the International Diabetes Federation, six million people in Germany currently have diabetes, and the number is expected to rise to about eight million by 2030. The European-wide surveys EUROASPIRE I-IV in patients with CIHD showed that most cardiovascular risk factors are not optimally treated [3]: Only one out of five patients achieved the target LDL-c values of < 70 mg/dl, and only the half have an adequate controlled blood pressure. A similar picture emerged regarding lifestyle factors: 16% of CIHD patients smoke, only one third of patients reported regular exercise, and about 60% have an increased abdominal waist circumference.

These numbers are alarming, especially regarding the fact that a lifestyle intervention including exercise training and dietary changes can reduce mortality by 20-30% and is therefore recommended in all current guidelines as a class I recommendation [4]. And successful lifestyle changes demonstrate positive long-term effects: patients with prediabetes still had a 27% lower incidence of diabetes 15 years after completing a lifestyle intervention [5]. Furthermore, there were many positive effects observed like weight reduction, improved

physical fitness, increased cognitive function, increased diabetes remission, and a significantly lower overall hospitalization rate [6].

Nevertheless, the implementation of such lifestyle interventions on a large scale in the German healthcare is still insufficient. In this context, the support of statutory health insurance funds has a central role and patients need to understand that they can influence their health and how to apply this self-empowerment [7].

Exercise and chronic ischemic heart disease

CIHD is characterized by atherosclerotic changes in the epicardial coronary arteries leading to regional underperfusion of myocardial segments of the affected coronary artery. The consequence is a local imbalance between oxygen demand and supply, leading to regional myocardial ischemia. The resulting clinical manifestations range from asymptomatic ischemia to stable angina and acute coronary syndrome.

Regular physical activity reduces cardiovascular morbidity and mortality [8] by positively influencing hypertension, dyslipidemia, abdominal obesity and (pre-) diabetes. Physical activity also improves exercise capacity and quality of life in patients [9].

A multimodal therapy management, such as increasing physical activity, the reduction of modifiable risk factors, and a change in diet shows the largest positive effect on the progression of CIHD.

Physical inactivity increases the risk of cardiovascular disease, with a doubling risk of premature cardiac death in inactive individuals. Compared to healthy individuals, patients with CIHD again have a higher mortality risk, which is also related to physical activity. And again, CIHD patients with low activity levels (<5 MET (metabolic equivalent task) [10]) have a worse prognosis of survival than patients who are physically active (>8 MET per week) (see Fig.1).

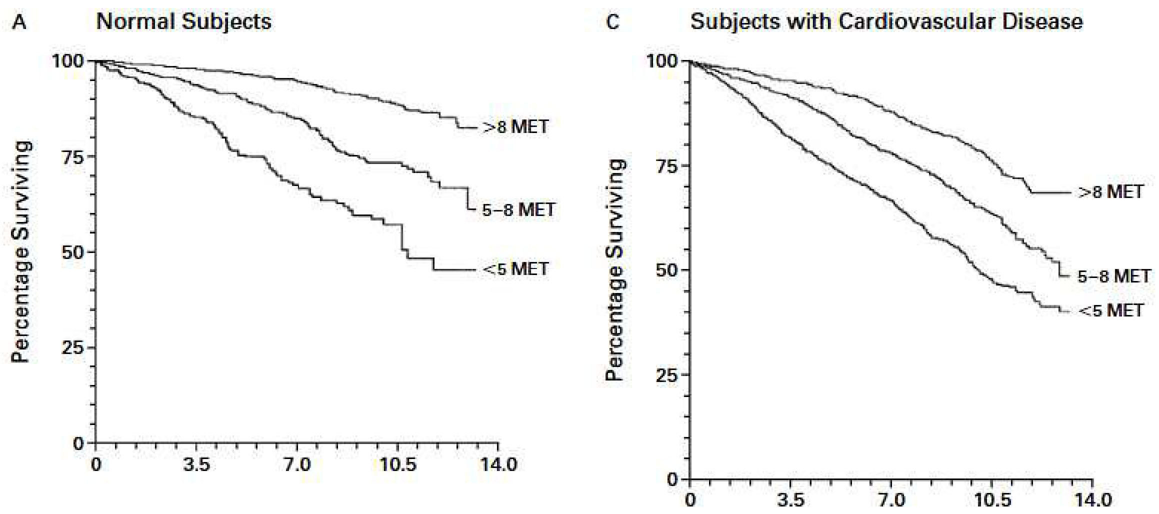

Figure 1 *Survival rate of healthy individuals (A) compared with patients with chronic ischemic heart disease (C) with different activity levels during one year*

Current national guidelines for secondary prevention and therapy of cardiovascular diseases recommend regular physical activity (endurance and resistance training) 3-5 weekly sessions, but more preferably physical activity on a daily base [9]. According to World Health Organization (WHO) recommendations, endurance sessions should include a minimum of 150 min moderate or 75 min vigorous physical activity per week. Moreover, resistance training should be added [4], and an increase in the amount of endurance and resistance training further improves the health benefit.

Exercise and diabetes mellitus type II

T2DM is a metabolic disease with an impaired glucose tolerance and the development of an insulin resistance leading to persistent elevated blood glucose levels (T2DM is diagnosed by an HbA_{1c} >6.5% (48 mmol/l) [11]).

In the western society, the prevalence of developing a T2DM during lifetime is 7.2% (♂ 7.0%, ♀ 7.4%, observed population aged 18-74 years) [12]. An impaired glucose tolerance increases with age [11].

T2DM increases the risk of cardiovascular disease and events by microvascular and macrovascular alterations, resulting in an increased mortality rate [11, 13]. Especially women with T2DM have an increased risk of cardiovascular disease [11, 13]. And diabetics have a worse recovery prognosis after acute cardiovascular events than non-diabetics (see Fig. 2) [14].

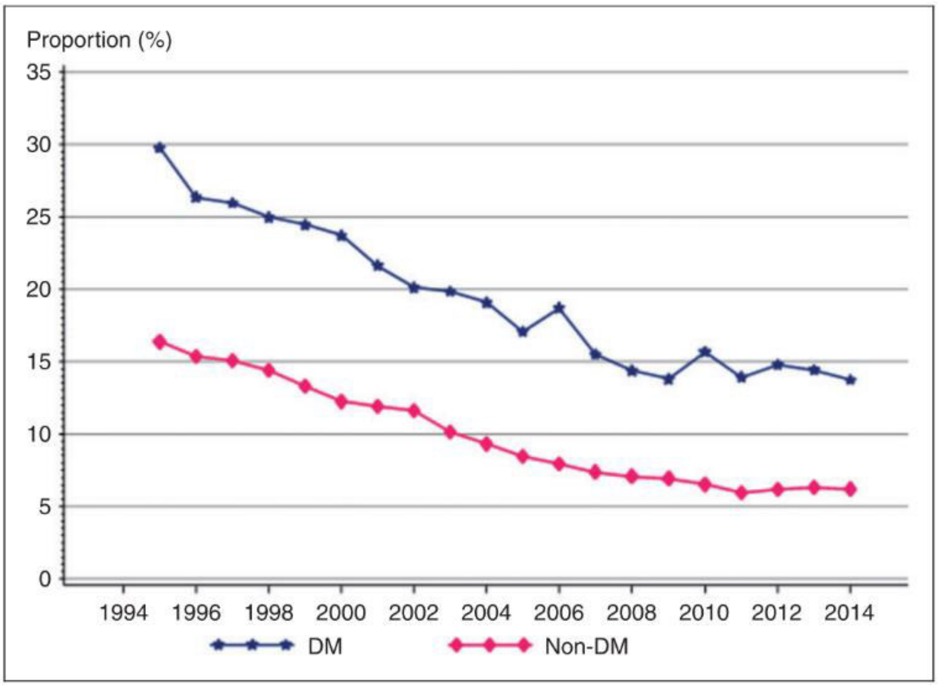

Figure 2 Difference in one-year survival probability between diabetic (DM) and nondiabetic (non-DM) patients after myocardial infarction [14].

In current guidelines, lifestyle changes are recommended as a level I, class A recommendation supported by evidence from several randomized controlled trials [11]. In this context, healthy dietary patterns, regular physical exercise, smoking cessation and weight reduction or stabilization are highlighted as the most important factors [11]. A previous lifestyle intervention of only 12 months already showed significant improvements in weight, HbA1c as well as other risk factors in diabetic patients [15].

Combined endurance and strength training has shown to lower HbA1c, blood pressure and blood lipids in T2DM patients, thus leading to a reduced cardiovascular risk. However, these effects can only be observed with regular, long-term physical activity [11]. To enhance this positive effect, an additional change in dietary habits and nutritional pattern should also be adopted.

3.1. Exercise training as therapy

First, before participating in the LeIKD programme, all patients undergo a medical cardiac examination to exclude existing contraindications for a safe (re-) start of regular physical exercise training. Therefore, a comprehensive examination will be performed. Based on these examinations, the intervention will be individually tailored according to the patient's physical condition.

In addition to medical history, physical examination and laboratory analyses, the first baseline study visit (time point t0) includes cardiopulmonary exercise testing (CPET) on a bicycle

ergometer. The latter enables the evaluation of medical risks, the determination of the maximum oxygen uptake as a parameter of exercise capacity and prognosis, and provides the relevant data to define the individual training intensity. Exercise training sessions are monitored via the LeIKD app which provides defined heart rate ranges and immediate feedback about the current training intensity. Thus, the patient can control and monitor the training sessions regarding intensity, volume and type according to the individual feedback via the LeIKD app.

The foundation of evidence-based physical activity is aerobic endurance training [9, 11]. The participation in exercise programs is relevant for all patients equally, since even patients with a pronounced exercise intolerance are likely to benefit from an increase in physical activity. An additional structured exercise training with a focus on strength-endurance, balance and mobility helps to achieve a multimodal training stimulus.

In LeiKD, patients perform the exercise training according to individual recommendations with consideration of their own exercise preferences provided by the LeIKD app. During the intervention period, training duration, frequency and intensity are gradually adjusted in accordance with the responsible sports scientists and physicians.

The individual perception of exertion and the training performance are evaluated via the LeIKD app and regular telephone feedback sessions with the patients. The overall goal is to achieve a home-based exercise training program independently performed by the patient and monitored via a telemedical approach.

4. Study objectives

Primary endpoint:

1. Change in HbA1c [Time Frame: 6 months]

   Measured in percent (%)

Secondary endpoints:

2. Change in HbA1c [Time Frame: 12 months]

   Measured in percent (%)

3. Change in health literacy [Time Frame: 6 and 12 months]

   European Health Literacy Survey Questionnaire (HLS-EU-Q16)

4. Change in daily physical activity [Time Frame: 6 and 12 months]

   International Physical Activity Questionnaire (IPAQ)

5. Change in average steps per day [Time Frame: 6 and 12 months]

   7-day average of steps/day measured by pedometers

6. Change in eating behaviour [Time Frame: 6 and 12 months]

   „Fragebogen zum Essverhalten“ (FEV; German questionnaire on eating behavior)

7. Change in quality of life [Time Frame: 6 and 12 months]

   Short form health survey (SF-36)

8. Change of medical care expenses [Time Frame: 6 and 12 months]

   Routine data of Health Insurance Company

9. Change in weight [Time Frame: 6 and 12 months]

   Measured in kilograms (kg)

10. Change in waist circumference [Time Frame: 6 and 12 months]

    Measured in centimetres (cm)

11. Change in LDL-cholesterol concentrations [Time Frame: 6 and 12 months]

    Measured in milligram/decilitre (mg/dL)

12. Change in HDL-cholesterol concentrations [Time Frame: 6 and 12 months]

    Measured in milligram/decilitre (mg/dL)

13. Change in triglyceride concentrations [Time Frame: 6 and 12 months]

    Measured in milligram/decilitre (mg/dL)

14. Change in systolic blood pressure [Time Frame: 6 and 12 months]

    Measured in millimetres of mercury (mmHg)

15. Change in diastolic blood pressure [Time Frame: 6 and 12 months]

Measured in millimetres of mercury (mmHg)

16. Number of the combined endpoint "4P-MACE" [Time Frame: 6 and 12 months]

    Cardiovascular deaths, non-fatal stroke, non-fatal myocardial infarction, hospitalization due to angina pectoris

5. Outcome measures

The endpoints will be operationalized using the following outcome parameters:

HbA1c value is used to determine whether a patient's diabetes is well controlled. It indicates the blood concentration of haemoglobin loaded with glucose and thus allows conclusions about blood glucose control of the last eight to twelve weeks.

Validated questionnaires are be used to measure **quality of life**, **health literacy**, **physical activity** and **eating behaviour**: Quality of life is assessed by the Short-Form 36 (SF-36) questionnaire, health literacy by the European Health Literacy Survey (HLS-EU- Q16) questionnaire, physical activity behaviour by the International Physical Activity Questionnaire - Short-Form (IPAQ) questionnaire and eating behaviour by the Eating Behaviour Questionnaire (FEV).

Among others, cardiovascular risk factors can be determined by measuring blood lipids. These include triglycerides, LDL and HDL cholesterol, as marker for an increased risk of atherosclerosis and lipid values are therefore collected as study outcome measures. Hypertension represents another relevant cardiovascular risk factor and therefore an outcome measure.

Changes in the demand for medical services are reflected in overall healthcare costs recorded by the statutory health insurance fund. In addition to overall health care costs, the differentiation between outpatient care costs, hospital care costs, and pharmaceutical costs is also a relevant outcome measure.

6. Study design

LeIKD is a multicentre randomized controlled lifestyle intervention trial in high-risk patients diagnosed with ischemic heart disease according to ICD-10 I20-I25 and diabetes mellitus type II (ICD-10 E11). The study design is shown in figure 3.

Participating centres:

1. centres in the urban regions: Berlin, Hamburg, Bochum and Munich
2. Centres in cities with a more rural area: Aachen, Bremen, Freiburg, Kassel, Göttingen, Greifswald, Magdeburg, Tübingen, Villingen-Schwenningen

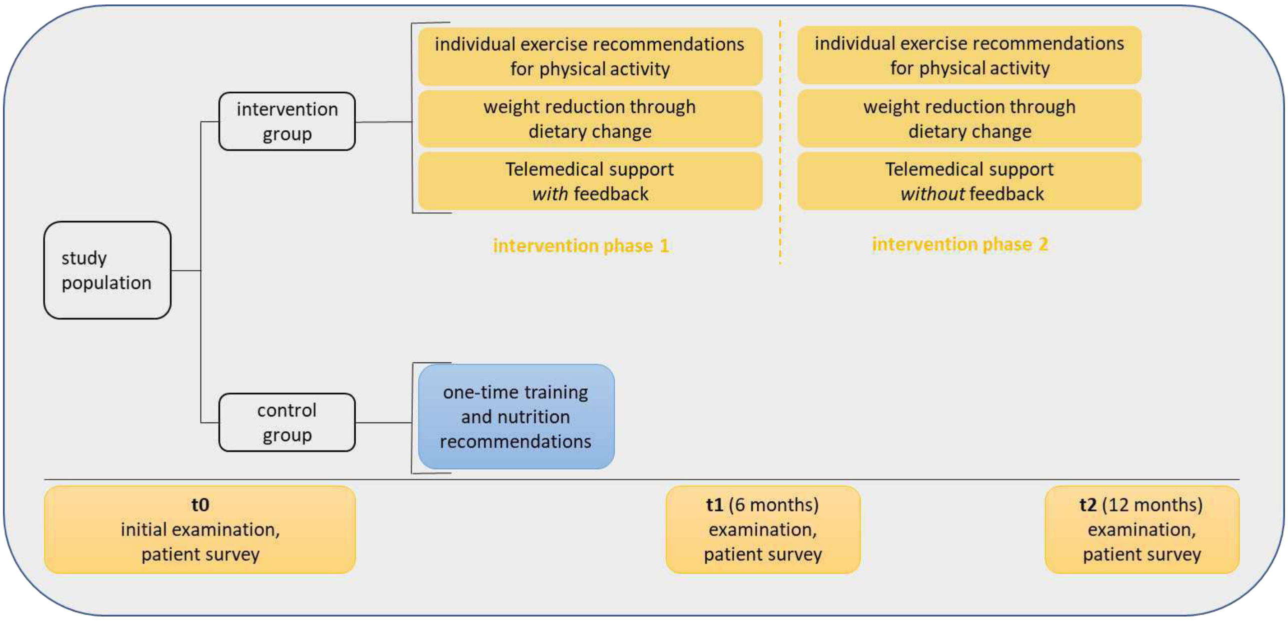

Figure 3 *LeIKD study design*

7. Study population

The target group of the lifestyle intervention are patients suffering DM II and CIHD in Germany. Eligible participants will be randomized into either intervention (IG) or control group (CG).

7.1. Inclusion and exclusion criteria

Inclusion and exclusion criteria for study participants and centres are presented in Table 1.

Table 1 *Inclusion and exclusion criteria for study patients and study centres*

Target group	Inclusion criteria	Exclusion criteria
CIHD and T2DM patients insured by TK health insurance	ischemic heart disease (ICD-10: I20-I25), Diabetes mellitus (ICD-10: E11), insured at participating health insurance, permission to perform exercise training by study physician, written informed consent	Mental and behavioural disorders (ICD-10: F0-F99), Heart failure NYHA IV (ICD-10: I50.14), Malignant neoplasm (ICD-10: C25, C34, C56, C72, C73, C78, C79, C97), Parkinson's disease (ICD-10: G20), Alzheimer's disease (ICD-10: G30), infantile

		cerebral palsy (ICD-10: G80), chronic kidney disease (ICD-10: N18.4 & N18.5), Trisomy 21 (ICD-10: Q90), Blindness / visual impairment (ICD-10: H54.0, H54.2, H54.3), Hearing loss (ICD-10: H90.0, H90.3, H90.5, H90.6, H90.8), Care level 1-5, Assured in a foreign country, Inability to exercise or conditions that may interfere with exercise intervention, No optimal medical treatment within the last 4 weeks, not clinically stable within the last 4 weeks, participation in another clinical trial
Study centres	Qualifications in internal medicine or sports medicine/cardiology	Not meeting the requirements

8. Study procedures

After initial contact by telephone, interested patients with CIHD and T2DM are invited to a screening visit at the corresponding local study centre, where they are again informed about the study project. After signing written informed consent, patients receive a detailed medical examination, including cardiopulmonary exercise testing. Patients were surveyed via questionnaires and subsequently randomized into IG and CG (time point t0). Cardiopulmonary exercise testing results will be evaluated for all participants at the Center for Prevention and Sports Medicine to define individual training recommendations, the evaluation of the exercise ECG will be performed at the local center. The EDC-system (secuTrial®) is used for randomization as well as for data collection after medical examinations and questionnaires. Randomization will be performed at the level of each local study center under the responsibility of the study investigator.

After baseline examination (t0), the IG receives individualized training recommendations, which are available via the LeIKD App. Patients in the CG receive general nutrition and exercise recommendations according to latest guideline recommendations. Moreover, all participants are provided with a pedometer to track their own daily physical activity. For

subsequent visits, both IG and CG patients will be re-examined at their study centre after six months (t1) and twelve months after the beginning of the intervention (t2).

The main goal in LeIKD is to encourage patients to be physically active and change their dietary patters to achieve and improve a more healthy behaviour in the participating T2DM and CIHD patients. For this purpose, patients will exercise home-based on their own empowered by the support of the LeIKD App.

At baseline (t0) inclusion and exclusion criteria will be checked, medical examinations and questionnaire surveys will take place at baseline (t0), after six months (t1) and after 12 months (t2). All examinations are shown in table 2. During the 12-month intervention period, patients' exercise frequency, type, volume, and intensity will be recorded via the LeIKD app and transferred to a database. In addition, the CG and IG will measure daily physical activity by pedometer.

***Table 2** LeIKD examinations*

	**t0**	**t1**	**t2**
**Time point (month)**	**0**	**6**	**12**
**Study inclusion**			
Written informed consent	X		
Inclusion/exclusion criteria	X		
Randomization	X		
**Assessments**			
Medication	X	X	X
Physical examination	X	X	X
ECG	X	X	X
Blood pressure	X	X	X
Anthropometry	X	X	X
Laboratory parameters[#]	X	X	X
Pedometer	X	X	X
Food protocol	X	X	X
Questionnaires	X	X	X
Adverse events		X	X
**Exercise performance**			
Stress-ECG	X	X	X
Cardiopulmonary-exercise testing	X	X	

Blood count, total cholesterol, triglycerides, HDL, LDL, urea, creatinine, potassium, sodium, GGT, GOT, HbA1c, glucose, NT-ProBNP

The questionnaires used during the LeIKD study are shown in table 3.

Table 3 Questionnaires during the LeIKD study

	t0	t1	t2
**Sociodemographics and usage behavior of mobile devices**	X		
**Quality of life (SF-36)**	X	X	X
**Health literacy (HLS-EU-Q16)**	X	X	X
**Smoking behaviour**	X	X	X
**Eating behaviour (FEV)**	X	X	X
**Physical activity (IPAQ)**	X	X	X

For the evaluation of changes in health care costs, the following data will be provided by the TK health insurance:

- Master data
- Medication data
- Work disability data
- Outpatient treatment data
- Inpatient treatment data
- Cost data

The study is divided into two study phases (Figure 3). In intervention phase I, after undergoing a comprehensive physical examination, the patient is introduced to exercise and dietary changes under supervision. Patients should be encouraged to engage in self-directed physical activity and adapted dietary behaviour during the course of the intervention.

In intervention phase II, the regular, independent implementation of the recommendations is strengthened, and feedback is given at extended intervals. In months 7 to 12, the sustainable consolidation of the recommendations will take place without additional feedback.

The individual sections set different priorities as follows:

<u>Exercise intervention</u>

Baseline visit t0 – week 3:

The first two weeks after the baseline examination t0 serve as a reference phase for daily activity (Pedometer) and for the IG additionally as a familiarization phase with the use of the LeIKD app. Patients in the IG do not yet receive training recommendations at t0, but are instructed for the use of the telemedical devices handed out by their study centre. They will be

encouraged to put on the chest strap at least once each week and start a "free workout" via the LeIKD app for a short time to become familiar with using the app and the devices.

Week 3 – 6-month visit (t1):

Individual feedback via telephone calls will be held at weeks 3, 5, 9, 13, 17, and 21 and will be used for training adjustments. In the first feedback call in week 3, it will be ensured that the patient is able to handle the technical devices (e.g. connect chest belt with the LeIKD app). At this point, the first training recommendations will be discussed. These recommendations are individually tailored, based on a combination of cardiopulmonary exercise testing results and the patient's own preferences.

For the definition of exercise intensities the program uses predefined intensity modules (regenerative, moderate and intensive continuous training and intensive interval training - the latter only in individual cases and after several weeks of stable and safe training). Furthermore, suggestions are made for daily activity based on step counts during the first two weeks. At the subsequent feedback calls at week 5, 9, 13, 17, and 21, training recommendations will be adjusted based on progress, adherence, and preferences of the participant.

6-month visit (t1) – week 29:

After the 6-month examination at study visit t1, the patient continues the training with the last recent training recommendations. The next adjustment will be made after the telephone feedback call in week 29.

Week 29 – 12-month Follow-Up-visit (t2):

In the 7th feedback call at week 29, the training recommendations are adjusted based on the exercise performance at study visit t1, as well as the previous exercise plan, previous adherence to the training and the preferences of the participant.

Dietary Intervention

Participants are asked to record their food intake at t0, week 5, week 14, t1 and t2. The food diaries will be reviewed based on energy density principles. In order to support the patients in their dietary changes, they will receive a feedback and nutritional recommendations at t0, week 5, week 14 and after t1.

9. Risk-benefit analysis

The main purpose of LeIKD is to introduce patients to lifestyle recommendations according to the current guidelines supported by the LeIKD app resulting in increased physical activity and improved diet. Therefore, it is assumed that the health risk is low. The patients are medically examined and monitored throughout the entire course of the study. The health benefits have already been described in detail in section *3. Scientific background.*

10. Biometrics

Both primary and secondary data will be used for evaluation. The statistical analyses will be performed considering a global level of significance of α=5 %.

The analyses and the reporting of results will be performed for IG and CG. The primary endpoint as well as all secondary endpoints will be considered in the analyses (if data basis is sufficient). Assuming sufficient participant numbers, the endpoints will also be examined at the level of the two subgroups ("urban areas," "cities with more rural areas") and, where appropriate, at the level of the study centres. A detailed description of the analyses will be presented in a separate statistical analysis plan.

10.1. Descriptive analysis

All endpoints will be analysed both a) between IG and CG (cross-sectional analysis) and b) over time (longitudinal analysis). First, the obtained data will be analysed descriptively. This includes absolute and relative frequency distributions.

In the course of analyzing metric variables - this primarily concerns resource consumption valued in monetary and non-monetary terms - measures of location (arithmetic mean, median and quartile) and measures of dispersion (variance, standard deviation) will be used. Furthermore, correlation analyses will be performed. Multivariate analysis methods will be used to determine dependencies between the variables.

10.2. Interferential statistical data analysis

In dependence on the variables considered, different statistical testing and multivariate procedures will be performed to test the defined hypotheses within the inferential statistical analysis.

In general, the inferential data analyses will focus on differences between IG and KG and differences between t0, t1, and t2. To analyse the course of the obtained values over the different time points, t-tests will be performed for IG and CG. Comparative analyses between

the two groups (while accounting for the effect over time) will be performed using corresponding statistical tests (e.g., repeated measures analysis of variance (ANOVA)).

The choice of tests depends on the distribution of the analysed variables in the population. In addition, multivariate analysis methods (especially regression analyses) will be used in isolated cases to examine the correlation between commonly occurring characteristics and to describe the correlation in more detail. Multivariate methods will be used to analyse factors influencing the primary and secondary endpoints.

10.3. Health economic analysis

The cost-effectiveness in LeIKD will be examined as part of the health economic evaluation in order to analyse the effect on the utilization of services and on service expenditures. This involves a quantitative assessment of resource consumption and a monetary evaluation of service utilization (statutory health insurance costs). In addition, the cost data will be merged with the benefit-related primary outcome variable in order to draw conclusions about the cost-benefit ratio of LeIKD. As the primary endpoint is the change in HbA1c, a cost-effectiveness analysis is performed considering the healthcare costs based on this endpoint.

11. Written informed consent

Before study enrolment, each study participant will be informed that participation in the study is voluntary and that he/she may withdraw from participation at any time without any reasons and without disadvantage afterwards. Written informed consent will be obtained from the patient prior to the start of the study. By signing the consent form, the patient declares his voluntary participation in the study and his intention to comply with the requirements of the study and the instructions of the investigator during the study.

The written informed consent will be available in two copies. One copy remains with the investigator, the other copy will be provided to the patient. The consent form is only valid after it has been signed and the patient is eligible to be included in the study, if he/she meets the inclusion and none of the exclusion criteria. With the written informed consent, the patient declares that he/she agrees to the recording of medical data within the study and to the forwarding of the data to the responsible supervisory authority or the responsible higher federal authority.

12. Data management and data protection

Collection, saving (type, location, duration) and transfer of data, guarantee of data security

The personal (case-related, medical and identifying) information (special protection requirement) of the test persons will be transferred between non-public bodies for the purpose of data security as well as control and monitoring of the exercise training (process data app). The legal foundation of the data transfer is the signed informed consent and patient consent form. The study data will be saved locally separated from other data (backend study server at University Hospital Klinikum rechts der Isar). The data will be collected directly from the patient. The automated collection of data without the involvement of the patient is only permitted if the direct collection would represent a non-reasonable effort (like data associated to the exercise training).

Both, the documentation of the medical examinations by the study physician and the patient surveys at each study site will be web-based in electronic form (electronic Case Report Forms) using the system secuTrial® of interActive Systems GmbH (Glogauer Straße 19, 10999 Berlin, http://www.secutrial.com). The compliance of interActive Systems GmbH with all data protection relevant requirements in handling personal health data will be specifically monitored by the evaluating institute (inav GmbH, Schiffbauerdamm 12, 10117 Berlin, 030 24 63 12 22) in the context of contract data processing.

While using the the LeIKD app (Developer: IDS Diagnostic Systems AG, Karlsburgstraße 2, 76227 Karlsruhe, Germany), heart rate data will be collected. The results of these measurements can be transmitted to a smartphone or tablet PC via Bluetooth wireless technology. From this device, the heart rate data will be forwarded to a server and a web platform of the University Hospital Klinikum rechts der Isar. Access to the data will only be granted by special access rights of authorized study personnel. The transfer of data via the smartphone or tablet PC's Internet connection is encoded. Private data (name, address, date of birth) are not transmitted. The transferred user data will be assigned to the corresponding user by a user ID on the web platform, enabling the responsible staff at the Klinikum rechts der Isar to perform an individual evaluation of the data related to each user. Data will be saved for 10 years. The data will not be forwarded to third parties.

Personal data ("master data", e.g. gender, year of birth, insurance status and start/end of insurance as well as information on early retirement status), information on hospital stays, if any (e.g. information on the type of stays, admission and discharge data as well as discharge diagnoses), information on diabetes medication (e.g. the categories, dosage sizes as well as

quantities of prescribed medication), expenses as well as information on cases of work incapacity, if any (e.g. the start and end of work incapacity) will be provided by the health insurance company. TK health insurance specifically supervises the adherence to all data protection-relevant specifications in the handling of the health insurance data. TK health insurance will not receive individual information on the results of any medical examinations.

Anonymized/ pseudonymized

All data will be saved pseudonymously in the database (backend server MRI).

Withdrawal, Data erasure

Each patient has the right to withdraw his or her study consent at any time. Subjects can request information about their personal data. The saved data will be reviewed at regular intervals during the course of the study and at the end of the study.

After withdrawal of consent to study participation by the patient and explicit demand, all personal data will be deleted. After study completion, the personal data collected during the study will be maintained for 10 years and then deleted.

"As far as the data is anonymized, the data protection regulations are not applicable and the further processing of the data is not subject to any legal restrictions." (except from data prtection authorithy: datenschutz-bayern.de). In general, study-related data must be preserved for a specific purpose (at least 10 years).

13. Biomaterial handling

In LeIKD, no further biomaterials are collected besides the local laboratory analysis at the study visits t0, t1 and t2.

14. Ethics committee

Before the study start, the study protocol and the informed consent form were submitted to the responsible ethics committee (Klinikum rechts der Isar, Chairman: Prof. Dr. Georg Schmidt) for approval.

The Ethics Committee will be notified immediately of any changes in the protocol that are not of a merely administrative nature, as well as of any serious adverse events that occur during

the study and could affect the participants' safety or the conduct of the study. Any recommendations and guidance from the Ethics Committee will be incorporated into the protocol as appropriate.

15. Legal basis

The legal framework for the healthcare concept is laid down in the "Selektiv-Vertrag" in accordance with §140a SGB V. In addition, structured treatment programs for coronary heart disease and diabetes, among others, have already been described since 2002 in accordance with §137f SGB V.

16. Subject insurance

All subjects will be insured within LeIKD via the local study center. There will be no separate study-specific transport or accident insurance.

The study will be conducted in compliance with applicable legal requirements and in accordance with the principles of the Declaration of Helsinki (1964 and revisions 1975, 1983, 1989, 1996, 2000, 2008), as well as in compliance with the requirements of the Federal Data Protection Act (as amended in 2009). The study will be reviewed by the Ethics Committee of the Klinikum rechts der Isar prior to the beginning of the study.

17. Literature

1. Emerging Risk Factors, C., et al., *Diabetes mellitus, fasting blood glucose concentration, and risk of vascular disease: a collaborative meta-analysis of 102 prospective studies.* Lancet, 2010. **375**(9733): p. 2215-22.
2. Thukkani, A.K., et al., *Long-Term Outcomes in Patients With Diabetes Mellitus Related to Prolonging Clopidogrel More Than 12 Months After Coronary Stenting.* J Am Coll Cardiol, 2015. **66**(10): p. 1091-101.
3. Kotseva, K., et al., *EUROASPIRE IV: A European Society of Cardiology survey on the lifestyle, risk factor and therapeutic management of coronary patients from 24 European countries.* Eur J Prev Cardiol, 2016. **23**(6): p. 636-48.
4. Piepoli, M.F., et al., *2016 European Guidelines on cardiovascular disease prevention in clinical practice: The Sixth Joint Task Force of the European Society of Cardiology and Other Societies on Cardiovascular Disease Prevention in Clinical Practice (constituted by representatives of 10 societies and by invited experts)Developed with the special contribution of the European Association for Cardiovascular Prevention & Rehabilitation (EACPR).* Eur Heart J, 2016. **37**(29): p. 2315-81.

5. Diabetes Prevention Program Research, G., *Long-term effects of lifestyle intervention or metformin on diabetes development and microvascular complications over 15-year follow-up: the Diabetes Prevention Program Outcomes Study.* Lancet Diabetes Endocrinol, 2015. **3**(11): p. 866-75.
6. Espeland, M.A., et al., *Impact of an intensive lifestyle intervention on use and cost of medical services among overweight and obese adults with type 2 diabetes: the action for health in diabetes.* Diabetes Care, 2014. **37**(9): p. 2548-56.
7. Sorensen, K., et al., *Health literacy and public health: a systematic review and integration of definitions and models.* BMC Public Health, 2012. **12**: p. 80.
8. Vatten, L.J., et al., *Adiposity and physical activity as predictors of cardiovascular mortality.* Eur J Cardiovasc Prev Rehabil, 2006. **13**(6): p. 909-15.
9. Bjarnason-Wehrens, B., et al., *Leitlinie körperliche Aktivität zur Sekundärprävention und Therapie kardiovaskulärer Erkrankungen.* Clinical Research in Cardiology Supplements, 2009. **4**(3): p. 1-44.
10. Ainsworth, B.E., et al., *Compendium of physical activities: an update of activity codes and MET intensities.* Med Sci Sports Exerc, 2000. **32**(9 Suppl): p. S498-504.
11. Ryden, L., et al., *ESC Guidelines on diabetes, pre-diabetes, and cardiovascular diseases developed in collaboration with the EASD: the Task Force on diabetes, pre-diabetes, and cardiovascular diseases of the European Society of Cardiology (ESC) and developed in collaboration with the European Association for the Study of Diabetes (EASD).* Eur Heart J, 2013. **34**(39): p. 3035-87.
12. Robert Koch-Institut, *Prävalenz des Diabetes mellitus. Gesundheitsberichterstattung des Bundes*. 2016.
13. Kannel, W.B. and D.L. McGee, *Diabetes and cardiovascular disease: The framingham study.* JAMA, 1979. **241**(19): p. 2035-2038.
14. Norhammar, A., L. Mellbin, and F. Cosentino, *Diabetes: Prevalence, prognosis and management of a potent cardiovascular risk factor.* European Journal of Preventive Cardiology, 2017. **24**(3_suppl): p. 52-60.
15. Pi-Sunyer, X., et al., *Reduction in weight and cardiovascular disease risk factors in individuals with type 2 diabetes: one-year results of the look AHEAD trial.* Diabetes Care, 2007. **30**(6): p. 1374-83.

Study protocol

Translation of the German Study Protocol

Version 1.1 (03.09.2019)

Track Change (Changes highlighted in yellow)

„Lifestyle Intervention in Chronic Ischemic Heart Disease and Diabetes”

(LeIKD)

Content

1. Project summary ... 3
2. Responsibilities ... 3
2.1. Principal investigator ... 3
2.2. Project leadership ... 4
2.3. Project partners ... 4
2.4. Funding ... 4
3. Scientific background ... 4
3.1. Exercise training as therapy ... 7
4. Study objectives ... 8
5. Outcome measures ... 10
6. Study design ... 10
7. Study population ... 11
7.1. Inclusion and exclusion criteria ... 11
8. Study procedures ... 12
9. Risk-benefit analysis ... 16
10. Biometrics ... 16
10.1. Descriptive analysis ... 16
10.2. Interferential statistical data analysis ... 17
10.3. Health economic analysis ... 17
11. Written informed consent ... 17
12. Data management and data protection ... 18
13. Biomaterial handling ... 20
14. Ethics committee ... 20
15. Legal basis ... 20
16. Subject insurance ... 20
17. Literature ... 21

1. Project summary

The majority of patients with chronic ischemic heart disease (CIHD) and diabetes mellitus type 2 (T2DM) is physically inactive and overweight. However, the successful implementation of an exercise and nutrition program in this population is not trivial as the experience from the past decades and previous projects has shown (e.g. "Sport als Therapie der Techniker Krankenkasse", a former lifestyle intervention project): expert knowledge is highly required to design individual tailored exercise and nutrition recommendations to achieve lifestyle changes in patients.

The “Lifestyle Intervention in Chronic Ischemic Heart Disease and Diabetes” (German title: **Le**bensstil- **I**ntervention bei **K**oronarer Herzkrankheit und **D**iabetes (LeIKD)) study addresses this point: The aim of the randomized controlled trial is to reduce cardiovascular risk factors and to increase health literacy in CIHD and T2DM patients by structured lifestyle intervention. The implementation of a home-based program with individual exercise and nutrition recommendations and supporting motivational strategies will take place in different regional areas in Germany. In addition to existing local medical care services, new telemedical approaches will also be applied like the developed LeIKD-exercise-training smartphone app. Therefore, a total of 1,500 patients with CIHD and TDM2 insured at the health insurance “Techniker Krankenkasse” (TK) will be included in the study. The primary endpoint is the reduction in glycated haemoglobin (HbA1c), secondary endpoints include changes in health literacy, quality of life, parameters of exercise capacity and dietary behaviour, and hospitalization rates.

The project is funded by the Innovation Fund and involies the following collaboration partners: Health insurance company “Techniker Krankenkasse” (Hamburg, Germany), Department of Prevention and Sports Medicine, University Hospital Klinikum rechts der Isar, Technical University of Munich (Munich, Germany), App-developer company IDS Diagnostic Systems AG (Karlsruhe, Germany) and the Institute for Applied Health Services Research inav (Berlin) as project evaluator.

2. Responsibilities

2.1. Principal investigator

Univ.-Prof. Dr. med. Martin Halle

Department of Prevention and Sports Medicine, University Hospital Klinikum rechts der Isar, Technical University Munich, Ismaninger Str. 22 Building 523, 81675 Munich

Study Coordination:

Stephan Müller (M.A.)

Department of Prevention and Sports Medicine, University Hospital Klinikum rechts der Isar, Technical University Munich, Ismaninger Str. 22 Building 523, 81675 Munich

2.2. Project leadership

Techniker Krankenkasse. M. Schultz. Bramfelder Straße 140. 22305 Hamburg

2.3. Project partners

Department of Prevention and Sports Medicine, University Hospital Klinikum rechts der Isar, Technical University of Munich, Univ.-Prof. Dr. med. M. Halle, Georg-Brauchle-Ring 56/58. 80992 Munich

Center for Prevention and Sports Medicine, University Hospital Klinikum rechts der Isar. Technical University Munich. Ismaninger Straße 22, Building 523. 81675 Munich

IDS Diagnostic Systems AG. Dr. med. B. Hackenberg. Karlsburgstraße 2. 76227 Karlsruhe

Privates Institut für angewandte Versorgungsforschung GmbH (inav). Univ.-Prof. Dr. V. Amelung. Schiffbauerdamm 12. 10117 Berlin.

2.4. Funding

Full funding is provided by the Federal Joint Committee (G-BA) (Germany); funding reference 01NVF17015.

3. Scientific background

CIHD and DM2 are among the most common causes of increased morbidity and mortality in Germany. Especially the combination of both diseases increases the risk exponentially [1]. The combined end point of death and myocardial infarction increased up to 30% after four years [2]. According to estimates by the International Diabetes Federation, six million people in Germany currently have diabetes, and the number is expected to rise to about eight million by 2030. The European-wide surveys EUROASPIRE I-IV in patients with CIHD showed that most cardiovascular risk factors are not optimally treated [3]: Only one out of five patients achieved the target LDL-c values of < 70 mg/dl, and only the half have an adequate controlled blood pressure. A similar picture emerged regarding lifestyle factors: 16% of CIHD patients smoke, only one third of patients reported regular exercise, and about 60% have an increased abdominal waist circumference.

These numbers are alarming, especially regarding the fact that a lifestyle intervention including exercise training and dietary changes can reduce mortality by 20-30% and is therefore recommended in all current guidelines as a class I recommendation [4]. And successful lifestyle changes demonstrate positive long-term effects: patients with prediabetes still had a 27% lower incidence of diabetes 15 years after completing a lifestyle intervention [5]. Furthermore, there were many positive effects observed like weight reduction, improved physical fitness, increased cognitive function, increased diabetes remission, and a significantly lower overall hospitalization rate [6].

Nevertheless, the implementation of such lifestyle interventions on a large scale in the German healthcare is still insufficient. In this context, the support of statutory health insurance funds has a central role and patients need to understand that they can influence their health and how to apply this self-empowerment [7].

Exercise and chronic ischemic heart disease

CIHD is characterized by atherosclerotic changes in the epicardial coronary arteries leading to regional underperfusion of myocardial segments of the affected coronary artery. The consequence is a local imbalance between oxygen demand and supply, leading to regional myocardial ischemia. The resulting clinical manifestations range from asymptomatic ischemia to stable angina and acute coronary syndrome.

Regular physical activity reduces cardiovascular morbidity and mortality [8] by positively influencing hypertension, dyslipidemia, abdominal obesity and (pre-) diabetes. Physical activity also improves exercise capacity and quality of life in patients [9].

A multimodal therapy management, such as increasing physical activity, the reduction of modifiable risk factors, and a change in diet shows the largest positive effect on the progression of CIHD.

Physical inactivity increases the risk of cardiovascular disease, with a doubling risk of premature cardiac death in inactive individuals. Compared to healthy individuals, patients with CIHD again have a higher mortality risk, which is also related to physical activity. And again, CIHD patients with low activity levels (<5 MET (metabolic equivalent task) [10]) have a worse prognosis of survival than patients who are physically active (>8 MET per week) (see Fig.1).

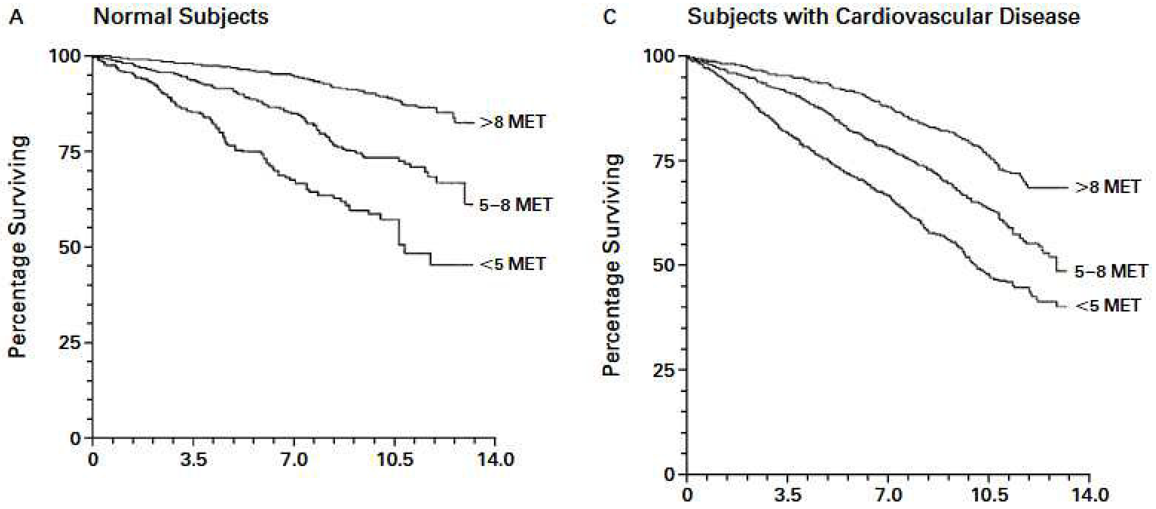

Figure 1 Survival rate of healthy individuals (A) compared with patients with chronic ischemic heart disease (C) with different activity levels during one year

Current national guidelines for secondary prevention and therapy of cardiovascular diseases recommend regular physical activity (endurance and resistance training) 3-5 weekly sessions, but more preferably physical activity on a daily base [9]. According to World Health Organization (WHO) recommendations, endurance sessions should include a minimum of 150 min moderate or 75 min vigorous physical activity per week. Moreover, resistance training should be added [4], and an increase in the amount of endurance and resistance training further improves the health benefit.

Exercise and diabetes mellitus type II

T2DM is a metabolic disease with an impaired glucose tolerance and the development of an insulin resistance leading to persistent elevated blood glucose levels (T2DM is diagnosed by an HbA_{1c} >6.5% (48 mmol/l) [11]).

In the western society, the prevalence of developing a T2DM during lifetime is 7.2% (♂ 7.0%, ♀ 7.4%, observed population aged 18-74 years) [12]. An impaired glucose tolerance increases with age [11].

T2DM increases the risk of cardiovascular disease and events by microvascular and macrovascular alterations, resulting in an increased mortality rate [11, 13]. Especially women with T2DM have an increased risk of cardiovascular disease [11, 13]. And diabetics have a worse recovery prognosis after acute cardiovascular events than non-diabetics (see Fig. 2) [14].

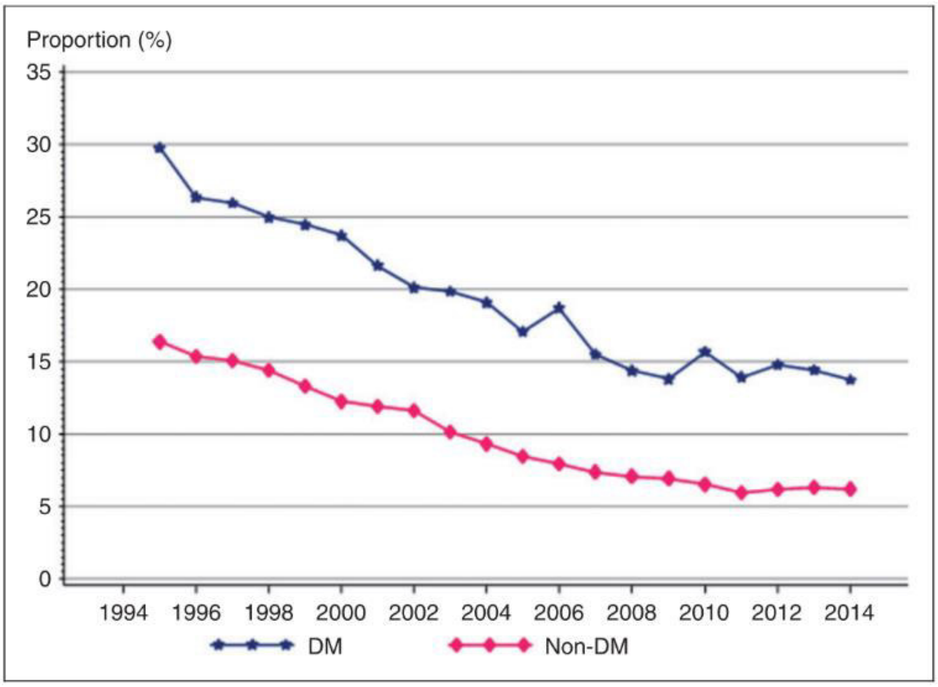

Figure 2 Difference in one-year survival probability between diabetic (DM) and nondiabetic (non-DM) patients after myocardial infarction [14].

In current guidelines, lifestyle changes are recommended as a level I, class A recommendation supported by evidence from several randomized controlled trials [11]. In this context, healthy dietary patterns, regular physical exercise, smoking cessation and weight reduction or stabilization are highlighted as the most important factors [11]. A previous lifestyle intervention of only 12 months already showed significant improvements in weight, HbA1c as well as other risk factors in diabetic patients [15].

Combined endurance and strength training has shown to lower HbA1c, blood pressure and blood lipids in T2DM patients, thus leading to a reduced cardiovascular risk. However, these effects can only be observed with regular, long-term physical activity [11]. To enhance this positive effect, an additional change in dietary habits and nutritional pattern should also be adopted.

3.1. Exercise training as therapy

First, before participating in the LeIKD programme, all patients undergo a medical cardiac examination to exclude existing contraindications for a safe (re-) start of regular physical exercise training. Therefore, a comprehensive examination will be performed. Based on these examinations, the intervention will be individually tailored according to the patient's physical condition.

In addition to medical history, physical examination and laboratory analyses, the first baseline study visit (time point t0) includes cardiopulmonary exercise testing (CPET) on a bicycle

ergometer. The latter enables the evaluation of medical risks, the determination of the maximum oxygen uptake as a parameter of exercise capacity and prognosis, and provides the relevant data to define the individual training intensity. Exercise training sessions are monitored via the LeIKD app which provides defined heart rate ranges and immediate feedback about the current training intensity. Thus, the patient can control and monitor the training sessions regarding intensity, volume and type according to the individual feedback via the LeIKD app.

The foundation of evidence-based physical activity is aerobic endurance training [9, 11]. The participation in exercise programs is relevant for all patients equally, since even patients with a pronounced exercise intolerance are likely to benefit from an increase in physical activity. An additional structured exercise training with a focus on strength-endurance, balance and mobility helps to achieve a multimodal training stimulus.

In LeiKD, patients perform the exercise training according to individual recommendations with consideration of their own exercise preferences provided by the LeIKD app. During the intervention period, training duration, frequency and intensity are gradually adjusted in accordance with the responsible sports scientists and physicians.

The individual perception of exertion and the training performance are evaluated via the LeIKD app and regular telephone feedback sessions with the patients. The overall goal is to achieve a home-based exercise training program independently performed by the patient and monitored via a telemedical approach.

4. Study objectives

Primary endpoint:

1. Change in HbA1c [Time Frame: 6 months]

   Measured in percent (%)

Secondary endpoints:

2. Change in HbA1c [Time Frame: 12 months]

   Measured in percent (%)

3. Change in health literacy [Time Frame: 6 and 12 months]

   European Health Literacy Survey Questionnaire (HLS-EU-Q16)

4. Change in daily physical activity [Time Frame: 6 and 12 months]

   International Physical Activity Questionnaire (IPAQ)

5. Change in average steps per day [Time Frame: 6 and 12 months]

   7-day average of steps/day measured by pedometers

6. Change in eating behaviour [Time Frame: 6 and 12 months]

   „Fragebogen zum Essverhalten“ (FEV; German questionnaire on eating behavior)

7. Change in quality of life [Time Frame: 6 and 12 months]

   Short form health survey (SF-36)

8. Change of medical care expenses [Time Frame: 6 and 12 months]

   Routine data of Health Insurance Company

9. Change in weight [Time Frame: 6 and 12 months]

   Measured in kilograms (kg)

10. Change in waist circumference [Time Frame: 6 and 12 months]

    Measured in centimetres (cm)

11. Change in LDL-cholesterol concentrations [Time Frame: 6 and 12 months]

    Measured in milligram/decilitre (mg/dL)

12. Change in HDL-cholesterol concentrations [Time Frame: 6 and 12 months]

    Measured in milligram/decilitre (mg/dL)

13. Change in triglyceride concentrations [Time Frame: 6 and 12 months]

    Measured in milligram/decilitre (mg/dL)

14. Change in systolic blood pressure [Time Frame: 6 and 12 months]

    Measured in millimetres of mercury (mmHg)

15. Change in diastolic blood pressure [Time Frame: 6 and 12 months]

Measured in millimetres of mercury (mmHg)

16. Number of the combined endpoint "4P-MACE" [Time Frame: 6 and 12 months]

    Cardiovascular deaths, non-fatal stroke, non-fatal myocardial infarction, hospitalization due to angina pectoris

5. Outcome measures

The endpoints will be operationalized using the following outcome parameters:

HbA1c value is used to determine whether a patient's diabetes is well controlled. It indicates the blood concentration of haemoglobin loaded with glucose and thus allows conclusions about blood glucose control of the last eight to twelve weeks.

Validated questionnaires are be used to measure **quality of life**, **health literacy**, **physical activity** and **eating behaviour**: Quality of life is assessed by the Short-Form 36 (SF-36) questionnaire, health literacy by the European Health Literacy Survey (HLS-EU- Q16) questionnaire, physical activity behaviour by the International Physical Activity Questionnaire - Short-Form (IPAQ) questionnaire and eating behaviour by the Eating Behaviour Questionnaire (FEV).

Among others, cardiovascular risk factors can be determined by measuring blood lipids. These include triglycerides, LDL and HDL cholesterol, as marker for an increased risk of atherosclerosis and lipid values are therefore collected as study outcome measures. Hypertension represents another relevant cardiovascular risk factor and therefore an outcome measure.

Changes in the demand for medical services are reflected in overall healthcare costs recorded by the statutory health insurance fund. In addition to overall health care costs, the differentiation between outpatient care costs, hospital care costs, and pharmaceutical costs is also a relevant outcome measure.

6. Study design

LeIKD is a multicentre randomized controlled lifestyle intervention trial in high-risk patients diagnosed with ischemic heart disease according to ICD-10 I20-I25 and diabetes mellitus type II (ICD-10 E11). The study design is shown in figure 3.

Participating centres:

1. centres in the urban regions: Berlin, Hamburg, Bochum and Munich
2. Centres in cities with a more rural area: Aachen, Bremen, Freiburg, Kassel, Göttingen, Greifswald, Magdeburg, Tübingen, Villingen-Schwenningen

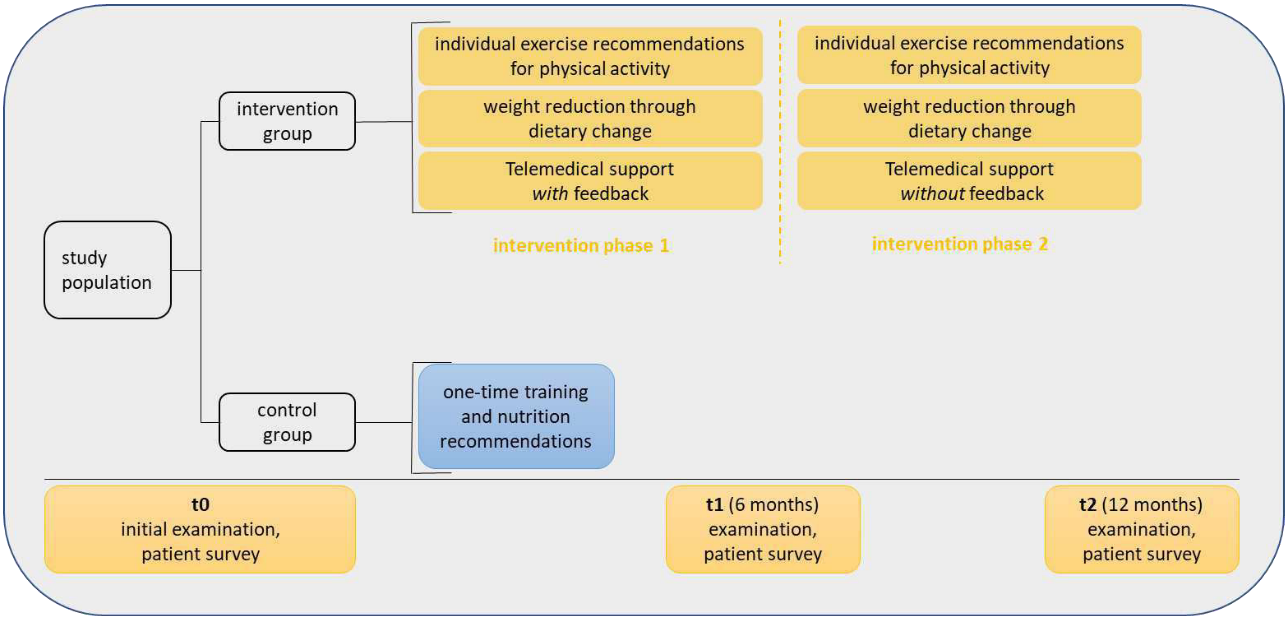

Figure 3 *LeIKD study design*

7. Study population

The target group of the lifestyle intervention are patients suffering DM II and CIHD in Germany. Eligible participants will be randomized into either intervention (IG) or control group (CG).

7.1. Inclusion and exclusion criteria

Inclusion and exclusion criteria for study participants and centres are presented in Table 1.

Table 1 *Inclusion and exclusion criteria for study patients and study centres*

Target group	Inclusion criteria	Exclusion criteria
CIHD and T2DM patients insured by TK health insurance	ischemic heart disease (ICD-10: I20-I25), Diabetes mellitus (ICD-10: E11) and HbA_{1c} ≥ 6.5 or anti-diabetic medication at the time of screening, insured at participating health insurance, permission to perform exercise training by study	Mental and behavioural disorders (ICD-10: F0-F99), Heart failure NYHA IV (ICD-10: I50.14), Malignant neoplasm (ICD-10: C25, C34, C56, C72, C73, C78, C79, C97), Parkinson's disease (ICD-10: G20), Alzheimer's disease (ICD-10: G30), infantile

	physician, written informed consent	cerebral palsy (ICD-10: G80), chronic kidney disease (ICD-10: N18.4 & N18.5), Trisomy 21 (ICD-10: Q90), Blindness / visual impairment (ICD-10: H54.0, H54.2, H54.3), Hearing loss (ICD-10: H90.0, H90.3, H90.5, H90.6, H90.8), Care level 1-5, Assured in a foreign country, Inability to exercise or conditions that may interfere with exercise intervention, No optimal medical treatment within the last 4 weeks, not clinically stable within the last 4 weeks, participation in another clinical trial
Study centres	Qualifications in internal medicine or sports medicine/cardiology	Not meeting the requirements

8. Study procedures

After initial contact by telephone, interested patients with CIHD and T2DM are invited to a screening visit at the corresponding local study centre, where they are again informed about the study project. After signing written informed consent, patients receive a detailed medical examination, including cardiopulmonary exercise testing. Patients were surveyed via questionnaires and subsequently randomized into IG and CG (time point t0). Cardiopulmonary exercise testing results will be evaluated for all participants at the Center for Prevention and Sports Medicine to define individual training recommendations, the evaluation of the exercise ECG will be performed at the local center. The EDC-system (secuTrial®) is used for randomization as well as for data collection after medical examinations and questionnaires. Randomization will be performed at the level of each local study center under the responsibility of the study investigator.

After baseline examination (t0), the IG receives individualized training recommendations, which are available via the LeIKD App. Patients in the CG receive general nutrition and exercise recommendations according to latest guideline recommendations. Moreover, all participants are provided with a pedometer to track their own daily physical activity. For

subsequent visits, both IG and CG patients will be re-examined at their study centre after six months (t1) and twelve months after the beginning of the intervention (t2).

The main goal in LeIKD is to encourage patients to be physically active and change their dietary patters to achieve and improve a more healthy behaviour in the participating T2DM and CIHD patients. For this purpose, patients will exercise home-based on their own empowered by the support of the LeIKD App.

At baseline (t0) inclusion and exclusion criteria will be checked, medical examinations and questionnaire surveys will take place at baseline (t0), after six months (t1) and after 12 months (t2). All examinations are shown in table 2. During the 12-month intervention period, patients' exercise frequency, type, volume, and intensity will be recorded via the LeIKD app and transferred to a database. In addition, the CG and IG will measure daily physical activity by pedometer.

***Table 2** LeIKD examinations*

	**t0**	**t1**	**t2**
**Time point (month)**	**0**	**6**	**12**
**Study inclusion**			
Written informed consent	X		
Inclusion/exclusion criteria	X		
Randomization	X		
**Assessments**			
Medication	X	X	X
Physical examination	X	X	X
ECG	X	X	X
Blood pressure	X	X	X
Anthropometry	X	X	X
Laboratory parameters#	X	X	X
Pedometer	X	X	X
Food protocol	X	X	X
Questionnaires	X	X	X
Adverse events		X	X
**Exercise performance**			
Stress-ECG	X	X	X
Cardiopulmonary-exercise testing	X	X	

Blood count, total cholesterol, triglycerides, HDL, LDL, urea, creatinine, potassium, sodium, GGT, GOT, HbA1c, glucose, NT-ProBNP

The questionnaires used during the LeIKD study are shown in table 3. In order to elaborate the experiences as well as the degree of contentment with the individual components of the lifestyle intervention from a patient's perspective in an explorative way, qualitative individual interviews and a focus group survey will be conducted. Therefore, patients from the IG are invited at the end of the 1st intervention phase (t1) and at the beginning of the 2nd intervention phase (t2). Both, the focus group and the individual interviews with IG participants will focus on subjective intervention experiences, barriers, and obstacles; in addition, they will highlight topic areas such as technology acceptance and adoption, as well as perceived impact on the area of health literacy from the patient's perspective. The qualitative analysis will be used to create a questionnaire to evaluate the project and provide relevant insights for a possible transfer of the lifestyle intervention LeIKD into routine care. This questionnaire will subsequently be used as part of the patient survey in the IG at survey time t2.

Table 3 Questionnaires during the LeIKD study

	t0	t1	t2
**Sociodemographics and usage behavior of mobile devices**	X		
**Quality of life (SF-36)**	X	X	X
**Health literacy (HLS-EU-Q16)**	X	X	X
**Smoking behaviour**	X	X	X
**Eating behaviour (FEV)**	X	X	X
**Physical activity (IPAQ)**	X	X	X
**Qualitative survey on contentment, hurdles and challenges**		X	X*

* Questionnaire is developed on the basis of a qualitative survey at time point t1

For the evaluation of changes in health care costs, the following data will be provided by the TK health insurance:

- Master data
- Medication data
- Work disability data
- Outpatient treatment data
- Inpatient treatment data
- Cost data

The study is divides into two study phases (Figure 3). In intervention phase I, after undergoing a comprehensive physical examination, the patient is introduced to exercise and dietary changes under supervision. Patients should be encouraged to engage in self-directed physical activity and adapted dietary behaviour during the course of the intervention.

In intervention phase II, the regular, independent implementation of the recommendations is strengthened, and feedback is given at extended intervals. In months 7 to 12, the sustainable consolidation of the recommendations will take place without additional feedback.

The individual sections set different priorities as follows:

Exercise intervention

Baseline visit t0 – week 3:

The first two weeks after the baseline examination t0 serve as a reference phase for daily activity (Pedometer) and for the IG additionally as a familiarization phase with the use of the LeIKD app. Patients in the IG do not yet receive training recommendations at t0, but are instructed for the use of the telemedical devices handed out by their study centre. They will be encouraged to put on the chest strap at least once each week and start a "free workout" via the LeIKD app for a short time to become familiar with using the app and the devices.

Week 3 – 6-month visit (t1):

Individual feedback via telephone calls will be held at weeks 3, 5, 9, 13, 17, and 21 and will be used for training adjustments. In the first feedback call in week 3, it will be ensured that the patient is able to handle the technical devices (e.g. connect chest belt with the LeIKD app). At this point, the first training recommendations will be discussed. These recommendations are individually tailored, based on a combination of cardiopulmonary exercise testing results and the patient's own preferences.

For the definition of exercise intensities the program uses predefined intensity modules (regenerative, moderate and intensive continuous training and intensive interval training - the latter only in individual cases and after several weeks of stable and safe training). Furthermore, suggestions are made for daily activity based on step counts during the first two weeks. At the subsequent feedback calls at week 5, 9, 13, 17, and 21, training recommendations will be adjusted based on progress, adherence, and preferences of the participant.

6-month visit (t1) – week 29:

After the 6-month examination at study visit t1, the patient continues the training with the last recent training recommendations. The next adjustment will be made after the telephone feedback call in week 29.

Week 29 – 12-month Follow-Up-visit (t2):

In the 7th feedback call at week 29, the training recommendations are adjusted based on the exercise performance at study visit t1, as well as the previous exercise plan, previous adherence to the training and the preferences of the participant.

Dietary Intervention

Participants are asked to record their food intake at t0, week 5, week 14, t1 and t2. The food diaries will be reviewed based on energy density principles. In order to support the patients in their dietary changes, they will receive a feedback and nutritional recommendations at t0, week 5, week 14 and after t1.

9. Risk-benefit analysis

The main purpose of LeIKD is to introduce patients to lifestyle recommendations according to the current guidelines supported by the LeIKD app resulting in increased physical activity and improved diet. Therefore, it is assumed that the health risk is low. The patients are medically examined and monitored throughout the entire course of the study. The health benefits have already been described in detail in section *3. Scientific background.*

10. Biometrics

Both primary and secondary data will be used for evaluation. The statistical analyses will be performed considering a global level of significance of α=5 %.

The analyses and the reporting of results will be performed for IG and CG. The primary endpoint as well as all secondary endpoints will be considered in the analyses (if data basis is sufficient). Assuming sufficient participant numbers, the endpoints will also be examined at the level of the two subgroups ("urban areas," "cities with more rural areas") and, where appropriate, at the level of the study centres. A detailed description of the analyses will be presented in a separate statistical analysis plan.

10.1. Descriptive analysis

All endpoints will be analysed both a) between IG and CG (cross-sectional analysis) and b) over time (longitudinal analysis). First, the obtained data will be analysed descriptively. This includes absolute and relative frequency distributions.

In the course of analyzing metric variables - this primarily concerns resource consumption valued in monetary and non-monetary terms - measures of location (arithmetic mean, median and quartile) and measures of dispersion (variance, standard deviation) will be used. Furthermore, correlation analyses will be performed. Multivariate analysis methods will be used to determine dependencies between the variables.

10.2. Interferential statistical data analysis

In dependence on the variables considered, different statistical testing and multivariate procedures will be performed to test the defined hypotheses within the inferential statistical analysis.

In general, the inferential data analyses will focus on differences between IG and KG and differences between t0, t1, and t2. To analyse the course of the obtained values over the different time points, t-tests will be performed for IG and CG. Comparative analyses between the two groups (while accounting for the effect over time) will be performed using corresponding statistical tests (e.g., repeated measures analysis of variance (ANOVA)).

The choice of tests depends on the distribution of the analysed variables in the population. In addition, multivariate analysis methods (especially regression analyses) will be used in isolated cases to examine the correlation between commonly occurring characteristics and to describe the correlation in more detail. Multivariate methods will be used to analyse factors influencing the primary and secondary endpoints.

10.3. Health economic analysis

The cost-effectiveness in LeIKD will be examined as part of the health economic evaluation in order to analyse the effect on the utilization of services and on service expenditures. This involves a quantitative assessment of resource consumption and a monetary evaluation of service utilization (statutory health insurance costs). In addition, the cost data will be merged with the benefit-related primary outcome variable in order to draw conclusions about the cost-benefit ratio of LeIKD. As the primary endpoint is the change in HbA1c, a cost-effectiveness analysis is performed considering the healthcare costs based on this endpoint.

11. Written informed consent

Before study enrolment, each study participant will be informed that participation in the study is voluntary and that he/she may withdraw from participation at any time without any reasons and without disadvantage afterwards. Written informed consent will be obtained from the patient prior to the start of the study. By signing the consent form, the patient declares his

voluntary participation in the study and his intention to comply with the requirements of the study and the instructions of the investigator during the study.

The written informed consent will be available in two copies. One copy remains with the investigator, the other copy will be provided to the patient. The consent form is only valid after it has been signed and the patient is eligible to be included in the study, if he/she meets the inclusion and none of the exclusion criteria. With the written informed consent, the patient declares that he/she agrees to the recording of medical data within the study and to the forwarding of the data to the responsible supervisory authority or the responsible higher federal authority.

12. Data management and data protection

Collection, saving (type, location, duration) and transfer of data, guarantee of data security

The personal (case-related, medical and identifying) information (special protection requirement) of the test persons will be transferred between non-public bodies for the purpose of data security as well as control and monitoring of the exercise training (process data app). The legal foundation of the data transfer is the signed informed consent and patient consent form. The study data will be saved locally separated from other data (backend study server at University Hospital Klinikum rechts der Isar). The data will be collected directly from the patient. The automated collection of data without the involvement of the patient is only permitted if the direct collection would represent a non-reasonable effort (like data associated to the exercise training).

Both, the documentation of the medical examinations by the study physician and the patient surveys at each study site will be web-based in electronic form (electronic Case Report Forms) using the system secuTrial® of interActive Systems GmbH (Glogauer Straße 19, 10999 Berlin, http://www.secutrial.com). The compliance of interActive Systems GmbH with all data protection relevant requirements in handling personal health data will be specifically monitored by the evaluating institute (inav GmbH, Schiffbauerdamm 12, 10117 Berlin, 030 24 63 12 22) in the context of contract data processing.

While using the the LeIKD app (Developer: IDS Diagnostic Systems AG, Karlsburgstraße 2, 76227 Karlsruhe, Germany), heart rate data will be collected. The results of these measurements can be transmitted to a smartphone or tablet PC via Bluetooth wireless technology. From this device, the heart rate data will be forwarded to a server and a web

platform of the University Hospital Klinikum rechts der Isar. Access to the data will only be granted by special access rights of authorized study personnel. The transfer of data via the smartphone or tablet PC's Internet connection is encoded. Private data (name, address, date of birth) are not transmitted. The transferred user data will be assigned to the corresponding user by a user ID on the web platform, enabling the responsible staff at the Klinikum rechts der Isar to perform an individual evaluation of the data related to each user. Data will be saved for 10 years. The data will not be forwarded to third parties.

Personal data ("master data", e.g. gender, year of birth, insurance status and start/end of insurance as well as information on early retirement status), information on hospital stays, if any (e.g. information on the type of stays, admission and discharge data as well as discharge diagnoses), information on diabetes medication (e.g. the categories, dosage sizes as well as quantities of prescribed medication), expenses as well as information on cases of work incapacity, if any (e.g. the start and end of work incapacity) will be provided by the health insurance company. TK health insurance specifically supervises the adherence to all data protection-relevant specifications in the handling of the health insurance data. TK health insurance will not receive individual information on the results of any medical examinations.

Anonymized/ pseudonymized

All data will be saved pseudonymously in the database (backend server MRI).

Withdrawal, Data erasure

Each patient has the right to withdraw his or her study consent at any time. Subjects can request information about their personal data. The saved data will be reviewed at regular intervals during the course of the study and at the end of the study.

After withdrawal of consent to study participation by the patient and explicit demand, all personal data will be deleted. After study completion, the personal data collected during the study will be maintained for 10 years and then deleted.

"As far as the data is anonymized, the data protection regulations are not applicable and the further processing of the data is not subject to any legal restrictions." (except from data prtection authorithy: datenschutz-bayern.de). In general, study-related data must be preserved for a specific purpose (at least 10 years).

13. Biomaterial handling

In LeIKD, no further biomaterials are collected besides the local laboratory analysis at the study visits t0, t1 and t2.

14. Ethics committee

Before the study start, the study protocol and the informed consent form were submitted to the responsible ethics committee (Klinikum rechts der Isar, Chairman: Prof. Dr. Georg Schmidt) for approval.

The Ethics Committee will be notified immediately of any changes in the protocol that are not of a merely administrative nature, as well as of any serious adverse events that occur during the study and could affect the participants' safety or the conduct of the study. Any recommendations and guidance from the Ethics Committee will be incorporated into the protocol as appropriate.

15. Legal basis

The legal framework for the healthcare concept is laid down in the “Selektiv-Vertrag” in accordance with §140a SGB V. In addition, structured treatment programs for coronary heart disease and diabetes, among others, have already been described since 2002 in accordance with §137f SGB V.

16. Subject insurance

All subjects will be insured within LeIKD via the local study center. There will be no separate study-specific transport or accident insurance.

The study will be conducted in compliance with applicable legal requirements and in accordance with the principles of the Declaration of Helsinki (1964 and revisions 1975, 1983, 1989, 1996, 2000, 2008), as well as in compliance with the requirements of the Federal Data Protection Act (as amended in 2009). The study will be reviewed by the Ethics Committee of the Klinikum rechts der Isar prior to the beginning of the study.

17. Literature

1. Emerging Risk Factors, C., et al., *Diabetes mellitus, fasting blood glucose concentration, and risk of vascular disease: a collaborative meta-analysis of 102 prospective studies.* Lancet, 2010. **375**(9733): p. 2215-22.
2. Thukkani, A.K., et al., *Long-Term Outcomes in Patients With Diabetes Mellitus Related to Prolonging Clopidogrel More Than 12 Months After Coronary Stenting.* J Am Coll Cardiol, 2015. **66**(10): p. 1091-101.
3. Kotseva, K., et al., *EUROASPIRE IV: A European Society of Cardiology survey on the lifestyle, risk factor and therapeutic management of coronary patients from 24 European countries.* Eur J Prev Cardiol, 2016. **23**(6): p. 636-48.
4. Piepoli, M.F., et al., *2016 European Guidelines on cardiovascular disease prevention in clinical practice: The Sixth Joint Task Force of the European Society of Cardiology and Other Societies on Cardiovascular Disease Prevention in Clinical Practice (constituted by representatives of 10 societies and by invited experts)Developed with the special contribution of the European Association for Cardiovascular Prevention & Rehabilitation (EACPR).* Eur Heart J, 2016. **37**(29): p. 2315-81.
5. Diabetes Prevention Program Research, G., *Long-term effects of lifestyle intervention or metformin on diabetes development and microvascular complications over 15-year follow-up: the Diabetes Prevention Program Outcomes Study.* Lancet Diabetes Endocrinol, 2015. **3**(11): p. 866-75.
6. Espeland, M.A., et al., *Impact of an intensive lifestyle intervention on use and cost of medical services among overweight and obese adults with type 2 diabetes: the action for health in diabetes.* Diabetes Care, 2014. **37**(9): p. 2548-56.
7. Sorensen, K., et al., *Health literacy and public health: a systematic review and integration of definitions and models.* BMC Public Health, 2012. **12**: p. 80.
8. Vatten, L.J., et al., *Adiposity and physical activity as predictors of cardiovascular mortality.* Eur J Cardiovasc Prev Rehabil, 2006. **13**(6): p. 909-15.
9. Bjarnason-Wehrens, B., et al., *Leitlinie körperliche Aktivität zur Sekundärprävention und Therapie kardiovaskulärer Erkrankungen.* Clinical Research in Cardiology Supplements, 2009. **4**(3): p. 1-44.
10. Ainsworth, B.E., et al., *Compendium of physical activities: an update of activity codes and MET intensities.* Med Sci Sports Exerc, 2000. **32**(9 Suppl): p. S498-504.
11. Ryden, L., et al., *ESC Guidelines on diabetes, pre-diabetes, and cardiovascular diseases developed in collaboration with the EASD: the Task Force on diabetes, pre-diabetes, and cardiovascular diseases of the European Society of Cardiology (ESC) and developed in collaboration with the European Association for the Study of Diabetes (EASD).* Eur Heart J, 2013. **34**(39): p. 3035-87.
12. Robert Koch-Institut, *Prävalenz des Diabetes mellitus. Gesundheitsberichterstattung des Bundes*. 2016.
13. Kannel, W.B. and D.L. McGee, *Diabetes and cardiovascular disease: The framingham study.* JAMA, 1979. **241**(19): p. 2035-2038.
14. Norhammar, A., L. Mellbin, and F. Cosentino, *Diabetes: Prevalence, prognosis and management of a potent cardiovascular risk factor.* European Journal of Preventive Cardiology, 2017. **24**(3_suppl): p. 52-60.
15. Pi-Sunyer, X., et al., *Reduction in weight and cardiovascular disease risk factors in individuals with type 2 diabetes: one-year results of the look AHEAD trial.* Diabetes Care, 2007. **30**(6): p. 1374-83.

Statistical Analysis Plan

LeIKD - Lifestyle intervention for chronic ischemic heart disease and type 2 diabetes

Confidential

Administrative information

Trial registration

Clinical trials.gov Identifier: NCT03835923

German registry for clinical studies (DRKS) Identifier: DRKS00015140

SAP Version

Version: 1.0

Date: 27.05.2021

Protocol version

Version: 1.1

Date: 30.09.2019

SAP Revision

Not applicable

Approved by:

**Prof. Dr. med. Martin Halle**	[signature]	28.05.2021
Principal investigator	Signature	Date
**Prof. Dr. Volker E. Amelung**	[signature]	27.05.2021
Evaluation project lead	Signature	Date

Prepared by:

**Janosch Krotz**	[signature]	27.05.2021
Statistician	Signature	Date

Content

1 Introduction 4
1.1 Background 4
1.2 Study design 4
1.3 Randomization 4
1.4 Trial population 4
1.4.1 Inclusion criteria 4
1.4.2 Exclusion criteria 5
1.5 Sample size calculation 5
1.6 Timing of study and outcome assessment 5
1.7 Statistical hypotheses and performance indicators 6
1.7.1 Health literacy (HLS-EU-Q16) 6
1.7.2 Physical activity (IPAQ) 6
1.7.3 Eating behaviour (FEV) 7
1.7.4 Health related quality of life (SF-36) 7
1.7.5 Physical activity (7-day average of steps/day) 8
1.7.6 Major adverse cardiac events (MACE) 8
1.7.7 Healthcare expenses (claims data) 8
1.8 Additional research questions 8
1.8.1 Patient satisfaction 8
1.8.2 Effects caused by Covid-19 9
2 STATISTICAL ANALYSIS 9
2.1 Statistical principals 9
2.2 Datasets for analysis 9
2.3 Treatment of missing data and lost to follow-up 9
2.4 Baseline characteristics 10
2.5 Assessment of primary endpoint 10
2.6 Assessment of secondary endpoints 10
2.7 Additional analysis 10
2.8 Covariables 11
2.9 Cost-effectiveness analysis 11
2.10 Subgroup analysis 13
2.11 Sensitivity analysis 13
2.12 Multiple testing 13
2.13 Interim analysis 13
2.14 Statistics software 13
2.15 Presentation of results 13
3 Abbrevations 13
4 References 14
5 Appendix: Baseline Characteristics 14

1 INTRODUCTION

Lifestyle intervention for chronic ischemic heart disease and type 2 diabetes (**Le**bensstil-**I**ntervention bei **K**oronarer Herzkrankheit und **D**iabetes; **LeIKD**) is an innovation funds project (reference number 01NVF17015) and conceptualised as a randomized controlled trail. The study has been approved by the ethics committee of the Technical University of Munich (registration number: 144/18-S) and at each study site.

The aim of this statistical analysis plan (SAP) is the description of statistical methods for the evaluation of LeIKD research hypothesis. Deviations to the SAP will be declared in the evaluation report. The SAP is based on the LeIKD study evaluation protocol from 8th of March 2018 and the updates from 29th of August 2019 and 25th of May 2020.

1.1 Background

The LeIKD program evaluates the effects of a combined exercise and nutrition intervention supported by telemedicine in patients suffering from chronic ischemic heart disease (CIHD) and type 2 diabetes mellitus (DM2). Primary endpoint is change in glycosylated haemoglobin (HbA1c in %) after 6 months in lifestyle intervention (LI) group compared to usual care (UC) group.

Following the baseline examination (t0) patients randomised to LI group will start LeIKD individualised exercise and nutrition intervention. Telemedical components include written (nutrition) and verbal (exercise) feedback from core laboratory and exercise training provided by LeIKD app (IDS Diagnostic Systems AG, Zehntwiesenstraße 35 b, 76275 Ettlingen, www.ids-ds.de). Patients randomised to usual care group will receive standardised exercise and dietary advice. For additional information about LeIKD intervention see von Korn et al. (2021).

1.2 Study design

LeIKD is conceptualised as non-blinded, multi-centric randomised controlled trail with two parallel groups (lifestyle intervention and usual care group).

1.3 Randomization

Block randomisation will be stratified for each study site. Allocation ratio is 1:1.

1.4 Trial population

The target population of LeIKD are adults (at least 18 years) in Germany diagnosed with CIHD and DM2. Potential participants of the LeIKD program will be selected and approached by Techniker Krankenkasse (TK) health insurance fund. Based on TK member data, patients diagnosed with CIHD (ICD-10 I20-I25) and DM2 (ICD-10 E11), living within 50 kilometres of study centres will be contacted by phone. Patients who indicate interest in program participation will be approached by study centres to make an appointment for baseline examination (t0). If physicians at study site conclude that eligibility criteria are met, patients may sign informed consent and participate in the program.

1.4.1 Inclusion criteria

- Ischemic heart disease (ICD-10: I20-I25)
- Diabetes mellitus (ICD-10: E11) and
- HbA1c ≥ 6.5 or anti-diabetic medication at the time of screening
- ≥18 years
- Insured at TK health insurance fund
- Permission to do physical exercises by study investigator

- Written informed consent

1.4.2 Exclusion criteria

- Mental and behavioral disorders (ICD-10: F00, F01, F02, F11, F12, F13, F14, F15, F16, F18, F20, F21, F22, F23, F24, F25, F28, F29, F44, F72, F73, F17, F84)
- Heart failure NYHA IV (ICD-10: I50.14)
- Malignant neoplasm (ICD-10: C25, C34, C56, C72, C73, C78, C79, C97)
- Parkinson's disease (ICD-10: G20)
- Alzheimer disease (ICD-10: G30)
- Infantile cerebral palsy (ICD-10: G80)
- Chronic kidney disease (ICD-10: N18.4 & N18.5)
- Trisomy 21 (ICD-10: Q90)
- Blindness/ visual impairment (ICD-10: H54.0, H54.2, H54.3)
- Hearing loss (ICD-10: H90.0, H90.3, H90.5, H90.6, H90.8)
- Care level 1-5
- Insured abroad
- Inability to do physical exercises or conditions that may interfere with exercise intervention
- No optimal medical treatment within the last 4 weeks
- Not clinically stable within the last 4 weeks
- Participation in another trial

1.5 Sample size calculation

Sample size calculation was based on the primary endpoint change in HbA1c after six months between groups. Assumptions for the initial sample size calculation were derived from the results of the ENHANCE trial (Sevick et al., 2012). A difference in mean between groups of 0.4 with a standard deviation of 1.8 was assumed. With a significance level of 5%, a statistical power of 80%, and an assumed dropout rate of 15%, a sample size of 750 was calculated. To compare effects of lifestyle intervention in rural and urban population, sample size was doubled to 1500 individuals to be included.

Due to insufficient recruitment and a fixed project duration, the aim to compare rural and urban population has been dropped and sample size calculation has been adjusted (updated in evaluation protocol from 29th of August 2019). Following the recommendations of Gignac and Szodorai (2016) an effect size of small to medium magnitude (0.305) was assumed. Based on a statistical power of 80%, a significance level of 5% and an increased dropout rate of 30%, a sample size of 486 patients was set as the minimum sample size to detect a relevant difference in the primary endpoint between LI and UC group.

1.6 Timing of study and outcome assessment

First patient was included at 18th of February 2019. Last patient out will be in April 2021 and database will be closed at 31st of May 2021.

Analyses will be performed by inav (privates Institut für angewandte Versorgungsforschung GmbH, Schiffbauerdamm 12, 10117 Berlin) until 30th of September 2021. Evaluation report will be written and handed in before 31st of March 2022.

Patients meeting eligibility criteria are randomised during initial examination (t0) to either LI or UC group (see figure 1). LI group will receive six months of lifestyle intervention (phase I). After phase I, a second examination (t1) for all participants will take place. After a further period of six months (phase II) the final examination (t2) will be conducted at study centres for both groups.

Figure 1 LeIKD study design

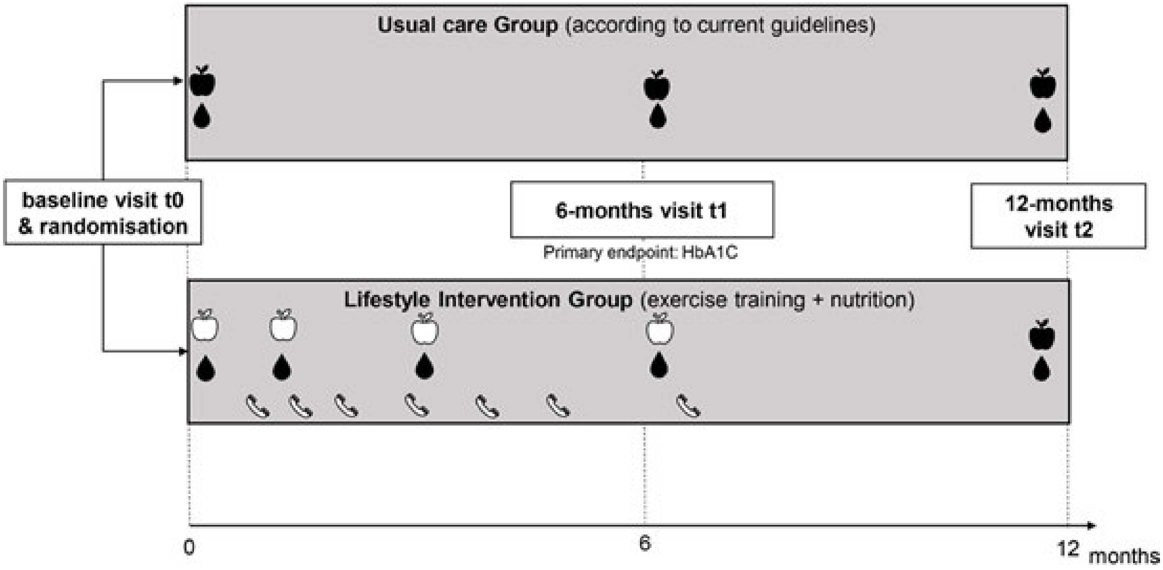

Apple black: food diary without feedback; apple white: food diary with feedback; blood drop: blood glucose profile without feedback; phone: feedback training from core laboratory. Source: von Korn et al. (2021).

1.7 Statistical hypotheses and performance indicators

Research questions, basic hypothesis and performance indicators have been formulated and published in LeIKD study evaluation protocol and von Korn et al. (2021). Endpoints are summarized in table 1.

Validated paper-based patient questionnaires filled in at study centres will be used for measurement of health literacy, physical activity, eating behaviour, and health related quality of life. Score building with data from standardised questionnaires will be summarized in sub-sections 1.7.1 to 1.7.4. Handling of data from pedometer, major adverse cardiovascular events (MACE) and claims data will be summarized in sub-sections 1.7.5 to 1.7.7. Additional information on measurement of clinical performance indicators is provided in the study protocol and von Korn et al. (2021).

1.7.1 Health literacy (HLS-EU-Q16)

Health literacy will be measured with the short version of the European Health Literacy Survey questionnaire HLS-EU-Q16 (HLS-EU Consortium, 2012; Pelikan, Röthlin, & Ganahl, 2013). Score creation follows the recommendations of scale developers. Missing values will be coded 0 and sum scores between 0 and 16 will be calculated. Cases will be excluded, if more than two items are missing.

1.7.2 Physical activity (IPAQ)

Physical activity will be captured with the “International Physical Activity Questionnaire (IPAQ)” (IPAQ Group, 2005). IPAQ-7 assesses physical activity over the last 7 days with a total of 7 items. IPAQ-Score will capture physical activity weighted by energy requirements expressed in MET-minutes. Individuals will be excluded in case of any missing values.

1.7.3 Eating behaviour (FEV)

Eating behaviour will be quantified with the "Fragebogen zum Essverhalten (FEV)" (Pudel & Westenhöfer, 1989). FEV is the German version of Three-Factor Eating Questionnaire (TFEQ) developed by Stunkard and Messick (1985). The validated German version comprises a total of 51 items and captures three factors: Cognitive restraint of eating (21 items) measures the extent participants can control their eating behaviour and restrict food intake to reduce or maintain weight. Disinhibition (16 items) expresses the extent respondents can be influenced in their eating behaviour by emotions or external stimuli. Hunger (14 items) captures the impact of hunger feelings on eating behaviour. For each subscale sum-scores between 0 and 21 ("Cognitive restraint"), 0 to 16 ("Disinhibition"), and 0 to 14 ("Hunger") will been formed. Each scale will be calculated if minimum 80% of items are available. Missing values will be replaced with individual mode imputation procedure.

Table 1 LeIKD Endpoints

Primary endpoint:
1a. Change in HbA1c (%) after 6 months between groups
**Secondary endpoints:**
1b. Change in HbA1c (%) after 12 months between groups
2. Change in health literacy after 6 and 12 months between groups, measured with HL-score (HLS-EU-Q16)
3. Change in daily physical activity after 6 and 12 months between groups, measured with<br>• MET-minutes (IPAQ)<br>• 7-day average of steps/day (by pedometers)
4. Change in eating behaviour after 6 and 12 months between groups, measured with<br>• Cognitive restraint score (FEV)<br>• Disinhibition score (FEV)<br>• Hunger score (FEV)
5. Change of quality of life after 6 and 12 months between groups, measured with<br>• Physical Component Score (PCS; SF-36)<br>• Mental Component Score (MCS; SF-36)
6. Change in cardiovascular risk factors after 6 and 12 months between groups measured with<br>• Body weight (kg)<br>• Waist circumference (cm)<br>• LDL-cholesterol concentrations (mg/dl)<br>• HDL-cholesterol concentrations (mg/dl)<br>• Triglyceride concentration (mg/dl)<br>• Systolic blood pressure, left arm (mmHg)<br>• Diastolic blood pressure, left arm (mmHg)<br>• Number of the combined endpoint '4-point MACE' (n/N, %)
7. Change in healthcare costs after 6 and 12 months between groups, measured with<br>• Total healthcare costs (€)<br>• Ambulatory healthcare costs (€)<br>• Stationary healthcare costs (€)<br>• Pharmaceutical healthcare costs (€)

FEV, Three-Factor Eating Questionnaire; HbA1C, glycosylated haemoglobin; HDL, high density cholesterol; HLS-EU-Q16, Health Literacy Survey Questionnaire; IPAQ, International Physical Activity Questionnaire; LDL, low density cholesterol; MACE, major cardiovascular events defined as cardiovascular death, non-fatal stroke, non-fatal myocardial infarction, hospitalisation due to angina pectoris; SF-36, Short Form-36 Questionnaire

1.7.4 Health related quality of life (SF-36)

Health related quality of life will be captured with the "Short Form 36 Health Survey (SF-36)" (Morfeld, Kirchberger, & Bullinger, 2011). The questionnaire includes 8 dimensions that are assigned to two subscales:

Physical and mental quality of life will be expressed as “Physical Component Score” (PCS) and “Mental Component Score” (MCS). For the evaluation of scales, regression weights based on US sample population will be used according to the questionnaire manual (Morfeld, Kirchberger & Bullinger, 2011). With item-specific weights scale items are transformed and standardized, resulting in scales with a mean of 50 and a standard deviation of 10 (norm-based scoring). The scores range from 0 to 100. Values smaller 50 indicate below average scores compared to standard population.

1.7.5 Physical activity (7-day average of steps/day)

7-day average of steps/day will be measured by pedometer AS80/AS87 (Beurer GmbH, Ulm, Germany). Both LI and UC group participants receive a pedometer and assistance to connect the tracking device with the smartphone at t0 examination. Patients who do not own a smartphone will receive a loaner device. The training program starts two weeks after t0 examination. Therefore, a 7-day average of steps/day will be calculated from the longest period of consecutive days with available data between days 4-14 (starting with days 4-10, 5-11, etc...) after t0 examination and will be considered as baseline value. For subsequent examinations (t1 and t2), the average from the longest period of consecutive days with available data between days 4-14 (starting with days 4-10, 5-11, etc...) before each examination will be used to calculate the 7-day average. Data will be considered missing, if less than 5 consecutive days of data are available.

1.7.6 Major adverse cardiac events (MACE)

Adverse events will be assessed by study centres and documented in electronic Case Report Form (eCRF) with the Electronic Data Capture Systems secuTrail® of the interactive Systems GmBH (Glogauer Starße 19, 10999 Berlin, www.secutrail.com). All adverse events will be assessed by a physician at Technical University of Munich, blinded to treatment arm assignment. Major adverse cardiovascular events (4-point MACE) will be defined as first occurrence of cardiovascular death, non-fatal stroke, non-fatal myocardial infarction, hospitalisation due to angina pectoris or coronary revascularization.

1.7.7 Healthcare expenses (claims data)

Use of medical services will be assessed with healthcare costs from claims data of German statutory health insurance system. Claims data will be provided by TK health insurance funds (Techniker Krankenkasse, Bramfelder Straße 140, 22305 Hamburg, www.tk.de) in pseudonymised form. Main indicators of interest are total healthcare costs of patients and the sub-components ambulatory, stationary, and pharmaceutical expenses.

Total healthcare expenses and sub-components will be provided by TK per calendar quarter. Ambulatory expenses will be considered at the day they occurred. Stationary expenses will be assigned to the day and respective quarter hospitalisation starts. Pharmaceutical expenses will be assigned to the quarter of dispensation. Healthcare expenses will be transformed to cover the respective phases of lifestyle intervention (phase I between t0 and t1 and phase II between t1 and t2). Expenses of a quarter will be proportionally assigned to the respective phase and expressed in expenses per six months. As processing of ambulatory claims data usually requires up to 9 months, ambulatory healthcare expenses for phase II might not be available for patients with t2 examination after 1st of October 2020.

1.8 Additional research questions

Complementing the endpoints for inference statistical analysis in table 1 two additional research questions are of interest and included in LeIKD study evaluation protocol.

1.8.1 Patient satisfaction

Patient satisfaction with LeIKD intervention is to be evaluated. A questionnaire for LI group will be designed based on a focus group discussion and exploratory qualitative guided interviews to explore satisfaction and

hurdles of LI group participants. Patient satisfaction questionnaire will be presented only to LI group during t2 examination. The satisfaction questionnaire will be analysed descriptively.

1.8.2 Effects caused by Covid-19

The appearance of SARS-CoV-2 in Germany has profound impact on public life since March 2020 and is expected to impact LeIKD lifestyle intervention. Therefore, an additional research questions has been added to study evaluation protocol dated 25th of May 2020: What effects on lifestyle intervention are caused by SARS-CoV-2?

All study sites had to be closed during March and April 2020 (individual sites were closed until June or during other periods). Covid-19 disease is of particular concern for the high aged multi-comorbid study population. Both DM2 and CHID are considered risk factors for Covid-19 complications. Therefore, study centre closures were necessary to protect patient's health.

Consequences on lifestyle intervention and data collection remain unclear. Additional subgroup analysis will be performed to evaluate effects of SARS-CoV2 on lifestyle intervention as well as interactions with other data. Participant specific factors are Covid-19 related risk factors (BMI, age), sociodemographic characteristics (living and employment situation), cluster specific effects (study centres) and the length and time of intervention phase I (see section 2.10).

2 STATISTICAL ANALYSIS

The aim of the statistical analysis is to compare changes in outcome over time (t0, t1 and t2) and study groups (LI and UC).

2.1 Statistical principals

In all analysis two-tailed p-values of 0.05 and/or 95%-confidence intervals will be used to declare statistical significance.

2.2 Datasets for analysis

Full analysis dataset (FAD) includes all randomised subjects who have not withdrawn informed consent. The statistical evaluation of the FAD will follow the intention-to-treat (ITT) principle and participants will be included as randomised.

Per-protocol dataset (PPD) includes all participants according to per-protocol (PP) principal. Protocol will be considered as violated if intervention group participants do not adhere to lifestyle intervention. Patients are defined as adherent if they accomplish a minimum of two-third of proposed training time and if they submit a minimum of two out of three food diaries during intervention phase I. Participants that are non-adherent will be excluded from PPD.

2.3 Treatment of missing data and lost to follow-up

To account for missing values of the primary endpoint in FAD, a multiple imputation approach will be applied as sensitivity analysis. Missing HbA1c values will be imputed using predictive mean matching procedure. Imputation will be performed under consideration of the baseline variables age, sex, body mass index, HbA1c, binary indicator for intake of insulin, binary indicator for intake of other anti-diabetic medication, binary indicator of CIHD classification (≤ 1-vessel coronary disease / unknown vs. ≥ 2-vessel coronary disease), binary indicator of education (low/medium vs. high), mobile user competencies (participant uses apps daily vs. participant uses apps less than daily) and treatment group. Ten datasets with imputed values will be generated and pooled to test the null hypothesis of no difference in change in HbA1c between groups (hypothesis test).

Comparisons will be performed on a significance level of 0.05 for each of the ten imputed datasets. Results will be aggregated, and 95%-confidence interval will be presented.

For all other analysis missing data of endpoints and covariables will be treated as such. To minimize missing data, the dataset will be assessed for missing values by statistician and study centres will be approached to enter missing values where possible.

A participant is considered lost to follow up, if the subject does not attend t2 follow-up examination. Trial completers are defined as subjects who attend t2 follow-up examination. Reasons for lost to follow-up will be summarized and include refusal to continue treatment or follow-up examination, moving away from study site, death, clinical reasons and so on. Data will be presented in CONSORT flow chart.

2.4 Baseline characteristics

Baseline characteristics of patients will be reported. Categorial variables will be presented with frequencies and percentages. Continuous variables will be summarized with arithmetic means and standard deviations. Frequencies of missing data will be displayed. Main baseline characteristics that will be summarized are listed in table 2 (see appendix).

2.5 Assessment of primary endpoint

The primary endpoint is the difference in change in HbA1c after 6 months between groups. Analyses will be performed with a significance level of 0.05. Change in HbA1c after 6 months will be calculated for patient i:

$$\Delta HbA1c_{i,1} = HbA1c_{i,t=1} - HbA1c_{i,t=0}$$

The null hypothesis states that no difference in change in HbA1c can be found between LI and UC groups.

$$H_0: \Delta HbA1c_1^{LI} = \Delta HbA1c_1^{UC}$$

The alternative hypothesis is that a difference in change in HbA1c can be found between groups.

$$H_A: \Delta HbA1c_1^{LI} \neq \Delta HbA1c_1^{UC}$$

Two-sided Student's t-test for independent samples will be used to compare changes in HbA1c after 6 months between groups (treatment effect).

2.6 Assessment of secondary endpoints

For all secondary endpoints, a significance level of 0.05 will be used to declare statistical significance and interpretation will be hypothesis generating. Change in outcome of endpoint x after 6 and 12 months between groups will be calculated for patient i and examination k ∈ {1,2}:

$$\Delta x_{i,k} = x_{i,t=k} - x_{i,t=0}$$

The null and alternative hypotheses can be expressed accordingly:

$$H_0^k: \Delta x_k^{LI} = \Delta x_k^{UC}$$

$$H_A^k: \Delta x_k^{LI} \neq \Delta x_k^{UC}$$

Two-sided Student's t-test for independent samples or Mann-Whitney test will be calculated to compare changes in outcome x between groups.

2.7 Adjusted analysis

For adjusted analysis multivariate regression will be performed to assess the relations between specific patient characteristics and change in endpoints (i.e. change in HbA1c). Regression models will include the respective baseline value (i.e. HbA1c at baseline), a dummy coded group variable (0 = UC, 1 = LI) and specified

covariables (see section 2.8) as fixed effects. The basic model will include age as continuous and gender as binary covariable. The model will be stepwise extended with the covariables comorbidity score and education. P-values of 0.05 will be used to declare statistical significance.

If model assumptions appear to be violated (mainly due to graphic analysis of residuals) transformation of variables or bootstrapped regression analysis will be considered. The chance of data containing outliers and influential cases due to incorrect values will be reduced by assessing the dataset for implausible values as defined by medical staff. Study centres will be asked to compare notified values with paper documentation and eventually enter corrections.

2.8 Covariables

Age

Age of participants at baseline expressed in years as documented by physicians' will be included as continuous covariable.

Gender

Gender of participants will be considered as categorical covariable. It will be coded as dummy variable for patient i:

$$Gender_i = \begin{cases} 1, participant\ is\ female \\ 0, participant\ is\ male \end{cases}$$

Comorbidity score

Elixhauser comorbidity score will be provided as part of claims data by TK health insurance funds. The comorbidity score is a method of categorising comorbidity of patients based on confirmed ICD-10 diagnosis from ambulatory, stationary and sick leave claims data at baseline (6 months before t0 examination). Data is weighted based on the association between comorbidity and death. The range of possible scores is between -19 (lesser comorbidity burden) and +89 (greater comorbidity burden).

Education

Education will be captured according to International Standard Classification of Education 1997 (ISCED-97) based on highest school leaving and professional qualifications. Other school leaving qualification will be coded as level 1 and other professional qualification will be included in level 3. Categories will be formed and included with two dummy variables:

$$Low\ Education_i = \begin{cases} 1, ISCED - 97\ level\ 1 - 2 \\ 0, otherwise \end{cases}$$

$$Medium\ Education_i = \begin{cases} 1, ISCED - 97\ level\ 3 - 4 \\ 0, otherwise \end{cases}$$

2.9 Cost-effectiveness analysis

Cost-effectiveness analysis (CEA) will be performed to compare the ratio of costs to effects for LI and UC group. Health care costs will reflect ambulatory, stationary and pharmaceutical expenses. Calculations for cost of LeIKD intervention per participant will be presented. An alternative cost value will be approximated assuming the implementation of LeIKD in standard care and used for sensitivity analysis.

Cost-effectiveness ratio (CER) for UC group will be calculated:

$$CER_{UC} = \frac{Average\ health\ cost\ of\ UC}{Average\ change\ in\ HbA1c\ of\ UC}$$

Cost-effectiveness ratio for LI group will be calculated:

$$CER_{LI} = \frac{Average\ health\ cost\ of\ LI}{Average\ change\ in\ HbA1c\ of\ LI}$$

The incremental cost effectiveness ratio (ICER) will be calculated, and sensitivity analysis will be performed for relevant parameters with uncertainty.

Table 3 Subgroups

Characteristic	Group	Categories
Age	LI and UC group	Age > median<br>Age ≤ median
Obesity	LI and UC group	BMI > 30<br>BMI ≤ 30
CIHD Classification	LI and UC group	≥ 2-vessel coronary disease<br>≤ 1-vessel coronary disease or unknown
HbA1c	LI and UC group	HbA1c > median<br>HbA1c ≤ median
VO2peak	LI and UC group	VO2peak > median<br>VO2peak ≤ median
Handling of technical devices	LI and UC group	Rather easy<br>Rather difficult or don’t know
Owns a mobile device	LI and UC group	Yes<br>No
Frequency use of apps	LI and UC group	Daily<br>Less than daily
Ever installed an app	LI and UC group	Yes<br>No or don’t know
PCS (SF-36)	LI and UC group	PCS ≤ median<br>PCS > median
MCS (SF-36)	LI and UC group	MCS ≤ median<br>MCS > median
Health literacy level (HLS-EU-Q16)	LI and UC group	Sufficient HL<br>Problematic or inadequate HL
Partnership status	LI and UC group	Yes<br>No
Federal State*	LI and UC group	BE<br>BW<br>BY<br>HE<br>MV<br>NW<br>SN<br>ST
Length of intervention phase I	LI and UC group	Phase I > 215 days<br>Phase I ≤ 215 days
Time of intervention phase I	LI and UC group	t1-excamination after 1st of April 2020<br>t1-excamination before 1st of April 2020

PCS: Physical Component Score; MCS: Mental Component Score; Health literacy level: Sufficient HL: HL-score > 12; problematic or inadequate HL: HL-score ≤ 12; BE: Berlin; BW: Baden-Württemberg; BY: Bayern; HE: Hessen; MV: Mecklenburg-Vorpommern; NW: Nordrhein-Westphalen; SN: Sachsen; ST: Sachsen-Anhalt; *Federal States with less than ten participants per group will be coded as one category.

2.10 Subgroup analysis

Characteristics assessed at baseline (t0) for subgroup analysis are listed in table 3. Aims of subgroup analysis include investigation of consistency of treatment effects across subgroups or exploration of treatment effects across subgroups in case the primary endpoint yields non-significant result. Each subgroup will be explored for differences in change in HbA1c between LI and UC group between subgroups. For that reason, the basic and the full regression model (see section 2.7) with change in HbA1c as independent variable will be extended with the subgroup category variable and the respective interaction term with the group variable. Statistical significance will be declared with a p-value < 0.05. Subgroup analysis will be interpreted exploratory.

2.11 Sensitivity analysis

To explore the robustness of the analysis of primary endpoints sensitivity analyses will be performed. The analysis strategy described in section 2.5 will be repeated with PP-Dataset (see section 2.2). Additional sensitivity analysis will be performed with imputed data as described in section 2.3.

2.12 Multiple testing

A significance level of 0.05 will declare statistical significance for all analyses. Secondary endpoints and subgroup analysis will be interpretated as exploratively.

2.13 Interim analysis

No interim analysis will be conducted.

2.14 Statistics software

Data analysis will be performed with statistical software R (R Development Core Team, R Foundation for Statistical Computing, Vienna, Austria).

2.15 Presentation of results

Values will be displayed with the same number of decimal places as collected. P-values will be reported as exact p-values, if equal or above 0.001. P-values below 0.001 will be displayed as "<.001".

3 ABBREVATIONS

BMI	Body Mass Index
CEA	Cost-effectiveness analysis
CER	Cost-effectiveness ratio
CIHD	Chronic ischemic heart disease
DM2	Type 2 diabetes mellitus
eCRF	electronic Case Report Form
FAD	Full analysis dataset
FEV	Fragebogen zum Essverhalten
HbA1c	Glycosylated haemoglobin
HLS-EU-Q16	European Health Literacy Survey
ICD	International Classification of Diseases
ICER	Incremental cost effectiveness ratio
IPAQ	International Physical Activity Questionnaire
ITT	Intention-to-Treat
LeIKD	Lifestyle intervention for chronic ischemic heart disease and type 2 diabetes (Lebensstil-Intervention bei Koronarer Herzkrankheit und Diabetes)

LI	Lifestyle Intervention
MACE	Major Adverse Cardiovascular Events
MCS	Mental Component Score of SF-36
NYHA	New York Heart Association
PCS	Physical Component Score of SF-36
PP	Per-protocol
PPD	Per-protocol dataset
RCT	Randomized Controlled Trial
SAP	Statistical Analysis Plan
SF-36	Short-Form 36 (Fragebogen zum Gesundheitszustand)
TFEQ	Three-Factor Eating Questionnaire
TK	Techniker Krankenkasse
UC	Usual Care

4 REFERENCES

Gignac, G. E., & Szodorai, E. T. (2016). Effect size guidelines for individual differences researchers. *Personality and Individual Differences, 102*, 74-78. doi:10.1016/j.paid.2016.06.069

HLS-EU Consortium. (2012). Comparative report of health literacy in eight EU member states. *The European health literacy survey HLS-EU.*

IPAQ Group. (2005). Guidelines for Data Processing and Analysis of the International Physical Activity Questionnaire (IPAG) - Short and Long Forms.

Morfeld, M., Kirchberger, I., & Bullinger, M. (2011). *SF-36: Fragebogen zum Gesundheitszustand*. Göttingen: Hogrefe.

Pelikan, J., Röthlin, F., & Ganahl, K. (2013). *Die Gesundheitskompetenz der österreichischen Bevölkerung– nach Bundesländern und im internationalen Vergleich. Abschlussbericht der Österreichischen Gesundheitskompetenz (Health Literacy)* Wien: Bundesländer-Studie LBIHPR Forschungsbericht.

Pudel, V., & Westenhöfer, J. (1989). *Fragebogen zum Ernährungsverhalten (FEV) [Questionnaire about eating behavior]*. Göttingen: Hogrefe.

Sevick, M. A., Korytkowski, M., Stone, R. A., Piraino, B., Ren, D., Sereika, S., . . . Burke, L. E. (2012). Biophysiologic outcomes of the Enhancing Adherence in Type 2 Diabetes (ENHANCE) trial. *J Acad Nutr Diet, 112*(8), 1147-1157. doi:10.1016/j.jand.2012.05.008

Stunkard, A. J., & Messick, S. (1985). The three-factor eating questionnaire to measure dietary restraint, disinhibition and hunger. *Journal of Psychosomatic Research, 29*(1), 71-83. doi:https://doi.org/10.1016/0022-3999(85)90010-8

von Korn, P., Sydow, H., Neubauer, S., Duvinage, A., Mocek, A., Dinges, S., . . . Halle, M. (2021). Lifestyle Intervention in Chronic Ischaemic Heart Disease and Type 2 Diabetes (the LeIKD study): study protocol of a prospective, multicentre, randomised, controlled trial. *BMJ Open, 11*(2), e042818. doi:10.1136/bmjopen-2020-042818

5 APPENDIX: BASELINE CHARACTERISTICS

Table 2 Baseline characteristics (selection)

Variable name		unit / categories
**Socio-demographic factors and lifestyle**		
Gender	n (%)	Female<br>Male<br>Divers
Age	M (SD)	Years
Highest school-leaving qualification	n (%)	No school-leaving qualification<br>Hauptschulabschluss<br>Realschulabschluss<br>Fachhochschulreife<br>Abitur<br>Other school-leaving qualification
Highest professional qualification	n (%)	No professional qualification

		Lehre<br>Fachschulabschluss<br>Fachhochschulabschluss<br>University degree<br>Other professional qualification
Education	n (%)	Low<br>Medium<br>High
Type of labor situation	n (%)	Not employed<br>Full-time employment<br>Part-time employment<br>In professional education<br>Irregular employment<br>Partial retirement<br>Other employment status
Partnership	n (%)	Yes<br>No
**Mobile devices**		
Handling of technical devices	n (%)	Rather easy<br>Rather difficult<br>Don't know
Owns a mobile device	n (%)	Yes<br>No
Ever installed an app	n (%)	Yes<br>No<br>Don't know
Frequency use of apps	n (%)	Daily<br>Weekly<br>Less<br>Never
**Lifestyle**		
Alcohol consumption	M (SD)	Alcoholic drinks per week
Smoking status	n (%)	Current<br>Never<br>Former
**Standardized Questionnaires**		
Health Literacy (HLS)		
HL-Score	M (SD)	Score
Eating Behavior (FEV)		
Cognitive restraint score	M (SD)	Score
Disinhibition score	M (SD)	Score
Hunger score	M (SD)	Score
Physical Activity (IPAQ)		
MET minutes	M (SD)	MET minutes
Health related Quality of life (SF-36)		
Physical health (PCS)	M (SD)	Score
Mental health (MCS)	M (SD)	Score
**Pedometer**		
Number of steps	M (SD)	steps/day
**Anamneses**		
Body weight	M (SD)	kg
Body height	M (SD)	cm
Waist circumference	M (SD)	cm
Body-Mass-Index (BMI)	M (SD)	kg/m^2
Obese (BMI > 30)	n (%)	Yes<br>No
Systolic blood pressure	M (SD)	mmHg
Diastolic blood pressure	M (SD)	mmHg
Chronic Ischemic Heart Disease Classification	n (%)	No relevant stenosis (< 50%)<br>1-vessel coronary disease<br>2-vessel coronary disease

		3-vessel coronary disease<br>Left main coronary disease<br>Unknown
Duration of known diagnosis of CIHD	M (SD)	Years
Duration of known diagnosis of DM2	M (SD)	Years
**Exercise Performance**		
Performance	M (SD)	Watt
VO2peak	M (SD)	ml/kg/min
**Laboratory Analysis**		
HbA1c	M (SD)	%
LDL-cholesterol concentrations	M (SD)	mg/dl
HDL-cholesterol concentrations	M (SD)	mg/dl
Triglyceride concentration	M (SD)	mg/dl
**Claims Data**		
Healthcare expenses		
Total health care expenses	M (SD)	€/6 months
Ambulatory expenses	M (SD)	€/6 months
Stationary expenses	M (SD)	€/6 months
Pharmaceutical expenses	M (SD)	€/6 months

M: mean; SD: standard deviation; FEV, Three-Factor Eating Questionnaire; HLS-EU-Q16, Health Literacy Survey Questionnaire; IPAQ, International Physical Activity Questionnaire; SF-36, Short Form-36 Questionnaire; N/A: not applicable
