# Supplementary material for: Telemedicine-supported lifestyle intervention for glycemic control in patients with CHD and T2DM: multicenter, randomized controlled trial
Source: Nat Med. 2025 Feb 7;31(4):1203–13. doi: 10.1038/s41591-025-03498-w (PMC12003154; doi:10.1038/s41591-025-03498-w)
Supplement: Supplementary file 2 — Reporting Summary [file 41591_2025_3498_MOESM2_ESM.pdf]

nature portfolio

Corresponding author(s): Martin Halle

Last updated by author(s): Dec 12, 2024

Reporting Summary

Nature Portfolio wishes to improve the reproducibility of the work that we publish. This form provides structure for consistency and transparency in reporting. For further information on Nature Portfolio policies, see our Editorial Policies and the Editorial Policy Checklist.

Statistics

For all statistical analyses, confirm that the following items are present in the figure legend, table legend, main text, or Methods section.

n/a	Confirmed	
☐	☒	The exact sample size (*n*) for each experimental group/condition, given as a discrete number and unit of measurement
☒	☐	A statement on whether measurements were taken from distinct samples or whether the same sample was measured repeatedly
☐	☒	The statistical test(s) used AND whether they are one- or two-sided<br>*Only common tests should be described solely by name; describe more complex techniques in the Methods section.*
☐	☒	A description of all covariates tested
☐	☒	A description of any assumptions or corrections, such as tests of normality and adjustment for multiple comparisons
☐	☒	A full description of the statistical parameters including central tendency (e.g. means) or other basic estimates (e.g. regression coefficient) AND variation (e.g. standard deviation) or associated estimates of uncertainty (e.g. confidence intervals)
☐	☒	For null hypothesis testing, the test statistic (e.g. *F*, *t*, *r*) with confidence intervals, effect sizes, degrees of freedom and *P* value noted<br>*Give P values as exact values whenever suitable.*
☒	☐	For Bayesian analysis, information on the choice of priors and Markov chain Monte Carlo settings
☒	☐	For hierarchical and complex designs, identification of the appropriate level for tests and full reporting of outcomes
☐	☒	Estimates of effect sizes (e.g. Cohen's *d*, Pearson's *r*), indicating how they were calculated

Our web collection on statistics for biologists contains articles on many of the points above.

Software and code

Policy information about availability of computer code

Data collection	We did not use computer code to collect the data in this study
Data analysis	Data analysis was performed using R Statistical Software (Version 4.0.2, Foundation for Statistical Computing), including the package 'mice'

For manuscripts utilizing custom algorithms or software that are central to the research but not yet described in published literature, software must be made available to editors and reviewers. We strongly encourage code deposition in a community repository (e.g. GitHub). See the Nature Portfolio guidelines for submitting code & software for further information.

Data

Policy information about availability of data

All manuscripts must include a data availability statement. This statement should provide the following information, where applicable:

- Accession codes, unique identifiers, or web links for publicly available datasets
- A description of any restrictions on data availability
- For clinical datasets or third party data, please ensure that the statement adheres to our policy

As patients have not explicitly consented to sharing pseudonymized data, we are not allowed to share the individual participant data for legal reasons. However, upon request to the corresponding author (martin.halle@mri.tum.de), aggregated data (data on demographics, clinical history, pharmacological treatment, electrocardiography, laboratory parameters, questionnaire data, cardiopulmonary exercise testing data) that does not allow identification of individual patients may

be shared after consultation with the data protection officers and legal representatives of the participating institutions and after signing a data sharing agreement. A response to requests for data access can be expected within four weeks.

Research involving human participants, their data, or biological material

Policy information about studies with human participants or human data. See also policy information about sex, gender (identity/presentation), and sexual orientation and race, ethnicity and racism.

Reporting on sex and gender	Self-reported sex is reported in the manuscript. Results of a pre-specified subgroup analysis including sex are shown in Extended Data Figure 1.
Reporting on race, ethnicity, or other socially relevant groupings	Self-reported ethnicity has been included under the heading "Patient Disposition" in the results section. Among the 499 evaluated patients, 98% were white.
Population characteristics	All patients had a documeted diagnosis of coronary heart disease and type 2 diabetes. Among the 499 analyzed patients, 84% were male. Mean age at randomization was 68 years. 92% had a history of arterial hypertension, 86% had hyperlipidemia, 23% had documented heart failure and 19% had atrial fibrillation. Additional baseline characteristics are shown in Table 1 of the manuscript.
Recruitment	Patients were recruited in a multi-stage process. All patients were insured at one health insurance fund (Techniker Krankenkasse) who initially contacted patients based on the recorded ICD-10 codes. If patients were interested to take part in the study, their contact details were referred to the corresponding local study sites to schedule an on-site screening visit. As the Techniker Krankenkasse is the largest health insurance fund in Germany, we believe that the study population can be described as a real-world population. However, there might be a natural self-selection of patients who are able and willing to participate in an exercise training study, which could lead to the fact that the average study patient may be less sick than the average patient in the entire population. That may explain the relatively low mean HbA1c at baseline, which may have limited the potential for further HbA1c reductions.
Ethics oversight	The study protocol had been approved by the ethics committee at Technical University of Munich and the local ethics committees at the Universities of Berlin, Aachen, Magdeburg, Dresden, Leipzig, Greifswald and Freiburg, and the ethics committees of the Medical Associations of Hesse and Baden-Württemberg.

Note that full information on the approval of the study protocol must also be provided in the manuscript.

Field-specific reporting

Please select the one below that is the best fit for your research. If you are not sure, read the appropriate sections before making your selection.

☒ Life sciences ☐ Behavioural & social sciences ☐ Ecological, evolutionary & environmental sciences

For a reference copy of the document with all sections, see nature.com/documents/nr-reporting-summary-flat.pdf

Life sciences study design

All studies must disclose on these points even when the disclosure is negative.

Sample size	For initial sample size calculation, a between-group difference in HbA1c of 0.4% with a standard deviation (SD) of 1.8% (effect size d=0.222) was assumed based on the results of the ENHANCE trial.31 With a power of 80%, a two-sided significance level of α=0.05 and an estimated drop-out rate of 15%, a total of 750 patients would have been required to detect a significant difference in the primary endpoint. However, administrative delays, insufficient recruitment rates at several study sites, and a fixed project duration required adjustments to the sample size calculation during the conduct of the study. Based on a higher effect size of d=0.305 with an estimated drop-out rate of 30%, we aimed to include at least 486 patients.
Data exclusions	Among the 502 randomized patients, 3 withdrew the consent to use their data. Therefore, the data from these patients has been removed from the dataset.
Replication	Analyses of the primary and secondary endpoints were repeated in sensitivity analyses (including a pre-scpecified per-protocol analysis and two post-hoc per-protocol analyses with altered per-protocol criteria for the primary and all secondary endpoints; and a sensitivity analysis with imputation of missing values for the primary endpoint)
Randomization	Eligible patients were randomly assigned (1:1) to the lifestyle intervention or usual care using a web-based system (secuTrial, interActive Systems GmbH, Berlin, Germany) stratified by study site with block sizes of four.
Blinding	Patients and staff were not blinded to treatment group assignments, which is a general limitation in lifestyle intervention trials

Reporting for specific materials, systems and methods

We require information from authors about some types of materials, experimental systems and methods used in many studies. Here, indicate whether each material, system or method listed is relevant to your study. If you are not sure if a list item applies to your research, read the appropriate section before selecting a response.

Materials & experimental systems

n/a	Involved in the study
☒	☐ Antibodies
☒	☐ Eukaryotic cell lines
☒	☐ Palaeontology and archaeology
☒	☐ Animals and other organisms
☐	☒ Clinical data
☒	☐ Dual use research of concern
☒	☐ Plants

Methods

n/a	Involved in the study
☒	☐ ChIP-seq
☒	☐ Flow cytometry
☒	☐ MRI-based neuroimaging

Clinical data

Policy information about clinical studies

All manuscripts should comply with the ICMJE guidelines for publication of clinical research and a completed CONSORT checklist must be included with all submissions.

Clinical trial registration	ClinicalTrials.gov: NCT03835923
Study protocol	The study protocol has been uploaded as supplementary material
Data collection	The data was collected in outpatient clinics or practices in Munich, two sites in Berlin, Aachen, Magdeburg, Dresden, Leipzig, Kassel, Greifswald, Freiburg, Villingen-Schwenningen (Germany) from February 12, 2019 (first-patient-first-visit) until April 16, 2021 (last-patient-last-visit).
Outcomes	The primary endpoint was the change in HbA1c after 6 months. Blood samples were drawn during study visits and analyzed by local laboratories. Secondary endpoints included change in HbA1c (%) after 12 months, as well as changes in HDL cholesterol, LDL cholesterol and triglycerides (analyzed by local laboratories), body weight (with validated weight standing scales), waist circumference (with measuring tape), systolic and diastolic blood pressure (with validated blood pressure monitors, on the left upper arm after 5 minutes of rest), health literacy (with the European Health Literacy questionnaire), quality of life physical and mental component scores (with the Short Form 36 questionnaire), eating behavior cognitive restraint of eating score, disinhibition and hunger scores (with the Three-Factor-Eating Questionnaire), daily physical acitivity (with the International Physical Activity Questionnaire), average steps per day (with pedometers) and major cardiovascular adverse events (documented by the local physicians and reviewed by a blinded phycisian at the study corelab) after 6 and 12 months.

Plants

Seed stocks	N/A
Novel plant genotypes	N/A
Authentication	N/A
